# Supplementary material for: Precise electrical gating of the single-molecule Mizoroki-Heck reaction
Source: Nat Commun. 2022 Aug 5;13:4552. doi: 10.1038/s41467-022-32351-8 (PMC9355990; doi:10.1038/s41467-022-32351-8)
Supplement: Supplementary file 1 — Supplementary Information [file 41467_2022_32351_MOESM1_ESM.pdf]

Supplementary Information

Precise electrical gating of the single-molecule Mizoroki-Heck reaction

Zhang et al.

Table of Contents

1. Molecular Synthesis and Macroscopic Experiments of the Mizoroki-Heck Cross-coupling Reaction 3
2. Single-molecule Connection Analysis 17
3. Machine Learning and Programs for Data Analysis 21
4. Experiments for the Attribution of Different Current Levels 25
5. Theoretical Calculation 30
6. Dynamic Analysis 36
7. Gate Tuning of the Single-molecule Mizoroki-Heck Reaction 43
8. Supplementary Movie 56
9. Atomic Coordinates 57
10. Supplementary References 112

1. Molecular Synthesis and Macroscopic Experiments of the Mizoroki-Heck Cross-coupling Reaction

Supplementary Note 1. Compound synthesis and characterisation

General information:

Unless otherwise noted, the chemicals and solvents were obtained from commercial sources and used without further purification. The reactions were monitored by thin-layer chromatography (TLC) with the products purified by column chromatography on silica gel (200-300 mesh, from Qingdao, China) if not noted otherwise. ^{1}H NMR spectra were recorded at 400 MHz while ^{13}C were recorded at 101 MHz with a Brucker ARX 400 spectrometer. The chemical shifts (δ) are reported in ppm while the coupling constants (J) are reported in hertz (Hz). Tetramethylsilane was used as the internal standard for the ^{1}H NMR spectra with a chemical shift at 0 ppm and $CDCl_3$ as the solvent. $CDCl_3$ was used as the internal standard for the ^{13}C NMR spectra with a chemical shift at 77 ppm. The following abbreviations are used to symbolise the multiplicities: s = singlet, d = doublet, t = triplet, q = quartet, dd = doublet of doublets, td = triplet of doublets, dt = doublet of triplets, ddd = doublet of doublet of doublets, and m = multiplet.

Synthetic Procedures:

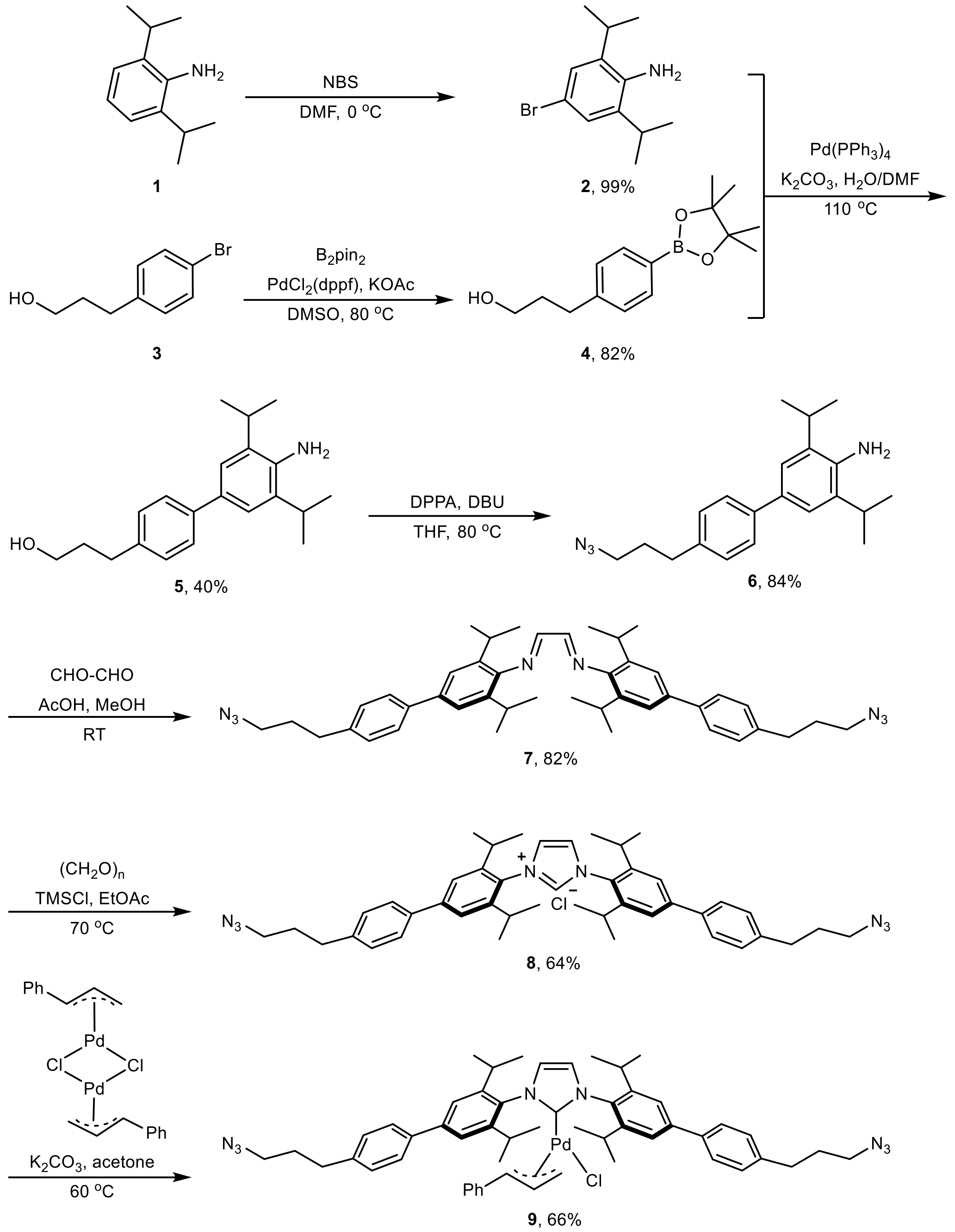

Supplementary Figure 1. Synthetic route of the *N*-heterocyclic carbene-palladium complex.

4-Bromo-2,6-diisopropylaniline (compound **2**):

Compound **2** was synthesised by a modified method from the Weisenfeld's work[1]. A solution of *N*-bromosuccinimide (NBS) (1.77 g, 10 mmol, 1.0 eq.) in *N*,*N*-dimethylformamide (10 mL) was added dropwise over 30 min to a stirred solution of 2,6-

diisopropylaniline (1.77 g, 10 mmol, 1.0 eq.) in *N*,*N*-dimethylformamide (20 mL) at 0°C. The reaction was stirred for another hour at 0°C, water (100 mL) was added. The resulting mixture was extracted with ethyl acetate and the combined organic layers were washed with saturated NH_4Cl solution (3 × 100 mL) followed by water (100 mL) and dried over anhydrous sodium sulfate. Solution concentration under vacuum afforded 2.562 g 4-bromo-2,6-diisopropylaniline (compound **2**) without purification, ~99% yield. Compound **2** was used directly for the following step.

3-(4-(4,4,5,5-Tetramethyl-1,3,2-dioxaborolan-2-yl)phenyl)propan-1-ol (compound **4**):
Compound **4** was synthesised according to Miyaura's work[2]. A flask charged with $PdCl_2$(dppf) (219 mg, 0.3 mmol), KOAc (1.47 g, 7 mmol), and B_2pin_2 (1.4 eq.) was flushed with nitrogen. DMSO (5 mL) and 3-(4-bromophenyl)propan-1-ol (1.08 g, 10 mmol, 1.0 eq.) were then added. After being stirred at 80°C for 24 h, the product was extracted with EtOAc, washed with water, and dried over anhydrous sodium sulfate and filtered with the filtrate concentrated in vacuo. Purification in a silica gel column (PE/EtOAc) afforded compound **4**, 2.150 g, ~82% yield. ^{1}H NMR (400 MHz, chloroform-*d*) δ 7.74 (d, *J* = 7.4 Hz, 2H), 7.22 (d, *J* = 7.4 Hz, 2H), 3.66 (t, *J* = 6.3 Hz, 2H), 2.72 (t, *J* = 7.6 Hz, 2H), 1.89 (p, *J* = 6.7 Hz, 2H), 1.34 (s, 12H). This result corresponds to results in the literature[3].

3-(4'-Amino-3',5'-diisopropyl-[1,1'-biphenyl]-4-yl)propan-1-ol (compound **5**):
A solution of compound **2** (2.562 g, 10 mmol, 1.0 eq.), compound **4** (2.622 g, 10 mmol, 1.0 eq.), $Pd(PPh_3)_4$ (693 mg, 0.6 mmol, 6 mol%), potassium carbonate (40 mmol, 5.528 g, 4.0 eq.), water and DMF (1:2, 45 mL) was heated at 100°C for 35 h. The reaction mixture was cooled, poured into half-saturated aq. sodium bicarbonate (100 mL) and extracted with (3 × 100 mL) EtOAc. The combined organic layers were dried over sodium sulfate, concentrated in vacuo and carefully purified in a silica gel column (PE/EtOAc or DCM) to give compound **5**, 1.256 g, ~40% yield. ^{1}H NMR (400 MHz, Chloroform-*d*) δ 7.48 (d, *J* = 8.1 Hz, 2H), 7.26 (s, 2H), 7.23 (d, *J* = 8.2 Hz, 2H), 3.69 (t, *J* = 6.4 Hz, 2H), 2.97 (hept, *J* = 6.8 Hz, 2H), 2.72 (dd, *J* = 8.7, 6.7 Hz, 2H), 1.97 – 1.86 (m, 2H), 1.32 (d, *J* = 6.8 Hz, 12H). ^{13}C NMR (101 MHz, Chloroform-*d*) δ 140.04, 139.70, 139.67, 132.77, 131.39, 128.72,

126.77, 121.73, 62.39, 34.34, 31.75, 28.14, 22.57. HRMS (ESI) calcd. for $C_{21}H_{30}NO^+$ $[M+H]^+$: 312.2322; found: 312.2316.

Note: a comparative amount of a by-product was also formed, 3-([1,1'-biphenyl]-4-yl)propan-1-ol, with very similar polarities of compound **5**. 3-([1,1'-biphenyl]-4-yl)propan-1-ol: ^{1}H NMR (400 MHz, Chloroform-*d*) δ 7.54 (d, *J* = 7.3 Hz, 2H), 7.48 (d, *J* = 8.1 Hz, 2H), 7.38 (t, *J* = 7.6 Hz, 2H), 7.28 (t, *J* = 7.4 Hz, 1H), 7.22 (d, *J* = 8.0 Hz, 2H), 3.63 (t, *J* = 6.5 Hz, 2H), 2.77 – 2.59 (m, 2H), 2.40 (s, 1H), 1.87 (dt, *J* = 13.5, 6.6 Hz, 2H). ^{13}C NMR (101 MHz, Chloroform-*d*) δ 141.13, 129.01, 128.88, 127.23, 127.19, 127.10, 62.20, 34.28, 31.83. The ^{1}H NMR data correspond to results in the literature[4].

4'-(3-Azidopropyl)-3,5-diisopropyl-[1,1'-biphenyl]-4-amine (compound **6**):

1,5-diazabicyclo(5,4,0)undec-5-ene (DBU) (104 mg, 0.68 mmol, 2.0 eq,) and THF (10 mL), diphenylphosphoryl azide (DPPA) (187 mg, 0.68 mmol, 2 eq.) was added dropwise to a mixture containing compound **5** (106 mg, 0.34 mmol, 1.0 eq.). The resulting mixture was stirred at 80°C for 25 h. The solvent was removed and the residue was extracted using EtOAc and water. The combined organic layers were dried over sodium sulfate and concentrated in vacuo and purified in a silica gel column (PE/EtOAc) to get 96 mg product, ~84% yield. ^{1}H NMR (400 MHz, Chloroform-*d*) δ 7.49 (d, *J* = 8.1 Hz, 2H), 7.26 (s, 2H), 7.21 (d, *J* = 7.9 Hz, 2H), 3.77 (s, 2H), 3.30 (t, *J* = 6.8 Hz, 2H), 2.96 (hept, *J* = 6.8 Hz, 2H), 2.71 (t, *J* = 7.6 Hz, 2H), 1.99 – 1.85 (m, 2H), 1.32 (d, *J* = 6.8 Hz, 12H). ^{13}C NMR (101 MHz, Chloroform-*d*) δ 140.34, 139.85, 138.66, 132.76, 128.77, 126.86, 121.73, 50.75, 32.43, 30.55, 28.18, 22.58. HRMS (ESI): calcd. for $C_{21}H_{29}N_4^+$ $[M+H]^+$: 337.2387; found: 337.2387.

N^1,N^2-bis(4'-(3-azidopropyl)-3,5-diisopropyl-[1,1'-biphenyl]-4-yl)ethane-1,2-diimine (compound **7**):

Glyoxal (40wt.% in H_2O, 40 μL, 0.35 mmol, 1.0 eq.) in MeOH (0.5 mL) was added to a solution of compound **6** (249 mg, 2.1 eq., 0.74mmol) in MeOH (0.5 mL) and AcOH (5 μL) previously warmed to 60°C. The resulting solution was stirred at room temperature, soon started to crystallise and then stirred for 12 more hours at room temperature. The resulting solution was put in a refrigerator for 1-2 hours and the bright yellow solid was filtered and

washed with small portions of MeOH until the filtrate color did not change (bright yellow). Drying on vacuum afforded compound **7**, 199 mg, ~82% yield. ^{1}H NMR (400 MHz, Chloroform-*d*) δ 8.16 (s, 2H), 7.56 (d, *J* = 8.3 Hz, 4H), 7.39 (s, 4H), 7.31 – 7.24 (m, 4H), 3.34 (t, *J* = 6.7 Hz, 4H), 3.01 (hept, *J* = 7.1 Hz, 4H), 2.77 (t, *J* = 7.6 Hz, 4H), 1.97 (p, *J* = 7.1 Hz, 4H), 1.27 (d, *J* = 6.8 Hz, 24H). ^{13}C NMR (101 MHz, Chloroform-*d*) δ 163.24, 147.32, 139.67, 139.59, 137.80, 137.29, 128.86, 127.20, 122.09, 50.69, 32.43, 30.49, 28.23, 23.49.

1,3-bis(4'-(3-azidopropyl)-3,5-diisopropyl-[1,1'-biphenyl]-4-yl)-1H-imidazol-3-ium chloride (compound **8**):

A solution of trimethylchlorosilane (TMSCl) (120 mg, 1.10 mmol, 1.03 eq.) in ethyl acetate (1 mL) was added dropwise to a solution of compound **7** (744 mg, 1.07 mmol, 1 eq.) and paraformaldehyde (99 mg, 1.10 mmol, 1.03 eq.) in ethyl acetate (6 mL) at 70°C. The resulting solution was stirred for 8 hours after addition at 70°C. After cooling to room temperature, the solvent was removed, dissolved in MeOH, and purified in a reverse phase column (C18) to get the desired product, 514 mg, ~64% yield. ^{1}H NMR (400 MHz, Chloroform-*d*) δ 10.49 (s, 1H), 8.00 (s, 2H), 7.53 (d, *J* = 7.7 Hz, 4H), 7.46 (t, *J* = 2.4 Hz, 4H), 7.32 (d, *J* = 7.8 Hz, 4H), 3.35 (t, *J* = 6.8 Hz, 4H), 2.79 (t, *J* = 7.7 Hz, 4H), 2.48 (ddt, *J* = 10.9, 7.0, 3.5 Hz, 4H), 1.97 (p, *J* = 7.1 Hz, 4H), 1.35 – 1.22 (m, 24H). ^{13}C NMR (101 MHz, Chloroform-*d*) δ 145.31, 144.81, 141.15, 139.96, 138.06, 129.12, 129.06, 127.60, 126.40, 123.40, 50.62, 32.44, 30.43, 29.30, 24.70, 23.76. HRMS (ESI): calcd. for $C_{45}H_{55}N_8^+$ [M-Cl]$^+$: 707.4544; found: 707.4536.

(1,3-bis(4'-(3-azidopropyl)-3,5-diisopropyl-[1,1'-biphenyl]-4-yl)-1,3-dihydro-2*H*-imidazol-2-ylidene)chloro(η^3-cinnamyl)palladium (compound **9**):

Compound **9** was synthesised according to the Nolan and Cazin's work[5]. Compound **8** (200 mg, 0.27 mmol, 2.1 eq.), [Pd(η^3 -cin)(μ-Cl)]$_2$ (67 mg, 0.13 mmol, 1.0 eq.), a magnetic stir bar and acetone (1.2 mL) were charged into a vial, followed by K_2CO_3 (72 mg, 0.52 mmol, 4.0 eq.). The mixture was stirred at 60°C for 24 h. After the reaction was complete, the solvent was removed under vacuum. The residue was purified in a silica gel column (PE/EtOAc) to get 166 mg product, ~66% yield. ^{1}H NMR (400 MHz, Chloroform-*d*) δ 7.60

(d, *J* = 7.7 Hz, 4H), 7.47 (d, *J* = 3.3 Hz, 4H), 7.31 (d, *J* = 7.8 Hz, 4H), 7.21 (s, 2H), 7.13 (qd, *J* = 11.1, 10.0, 4.0 Hz, 5H), 5.19 – 5.01 (m, 1H), 4.36 (d, *J* = 12.6 Hz, 1H), 3.35 (t, *J* = 6.6 Hz, 4H), 3.20 – 2.95 (m, 5H), 2.79 (t, *J* = 7.5 Hz, 4H), 1.98 (p, *J* = 7.0 Hz, 4H), 1.86 (d, *J* = 11.7 Hz, 1H), 1.42 (dd, *J* = 25.3, 6.9 Hz, 12H), 1.27 – 1.11 (m, 12H). ^{13}C NMR (101 MHz, Chloroform-*d*) δ 185.42, 146.44, 142.29, 140.39, 139.03, 137.96, 135.20, 128.97, 128.91, 128.27, 127.51, 127.36, 126.69, 124.45, 122.64, 108.96, 89.94, 50.68, 46.69, 32.46, 30.49, 28.77, 26.28, 26.18, 23.11, 22.95.

Macroscopic experiments of Mizoroki-Heck cross-coupling reaction:

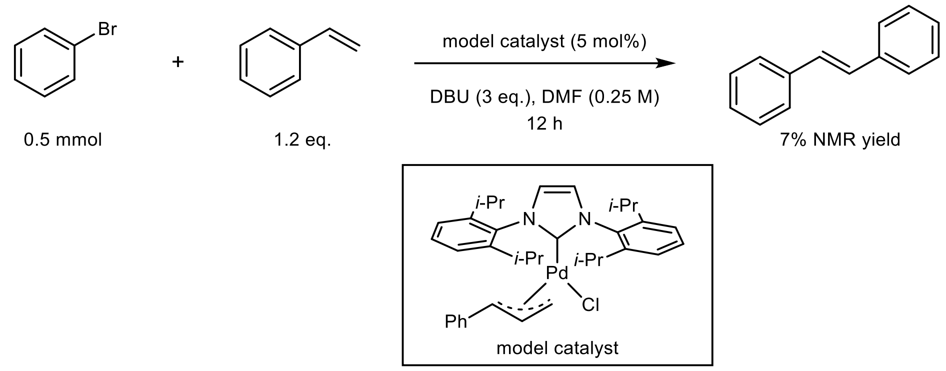

In a 20-mL vial, bromobenzene (53 μL, 0.5 mmol), styrene (69 μL, 1.2 eq.), DMF (2 mL), model catalyst (16 mg, 5 mol%) and 1,8-diazabicyclo[5.4.0]undecane-7-ene (DBU, 224 μL, 3.0 eq.) were added successively. The vial was sealed and heated at 120 °C for 12 h. Then, cooled to room temperature, 50 mL ethyl acetate was added, washed with water three times. The organic phase was dried by Na_2SO_4 and the solvent was removed under vacuum. The obtained residue was analysed by ^{1}H NMR with adding 1,3,5-trimethoxybenzene as internal standard. According to the literature report of the chemical shift of cross-coupling product, the yield was determined as ~7%, *E*/*Z* >20. Then, the cross-coupling product was separated in a silica gel column (PE/EtOAc), and has been characterised by NMR spectra. ^{1}H NMR (400 MHz, Chloroform-*d*) δ 7.55 – 7.48 (m, 4H), 7.35 (t, *J* = 7.7 Hz, 4H), 7.29 – 7.21 (m, 2H), 7.11 (s, 2H).[6]

The yield of the macroscopic experiment was relatively low. The main reasons were the unoptimized reactions conditions and side reactions of the model catalyst molecule during pre-activation process.[7]

Synthesis of substrates for signal attribution experiments and fluorescence emission:

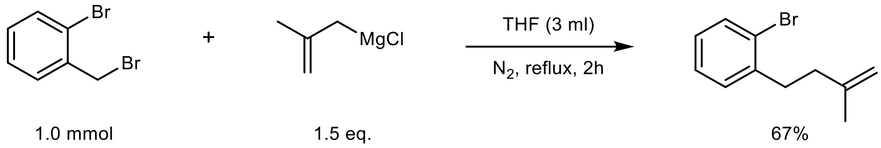

In an oven dried, 10 mL round bottom flask, equipped with a magnetic stir bar and septum under nitrogen, was placed 2-bromobenzyl bromide (250 mg, 1.0 mmol), and dry THF (3 mL). The flask was placed in an ice water bath and (2-methylallyl)magnesium chloride (1.0 M in Et_2O, 1.5 mL, 1.5 eq.) was added via syringe. The stirring solution was then fitted with a condenser and refluxed under nitrogen for 2 h at which point TLC indicated that the reaction was complete. The reaction was quenched by addition of sat. NH_4Cl solution (20 mL), extracted (3 × 2 mL, Et_2O), the combined organic layers were dried (Na_2SO_4) and concentrated under vacuum to yield the desire product (150 mg, 67%). ^{1}H NMR (400 MHz, Chloroform-*d*) δ 7.59 – 7.48 (m, 1H), 7.23 – 7.19 (m, 2H), 7.04 (ddd, *J* = 7.9, 5.1, 3.9 Hz, 1H), 4.82 – 4.72 (m, 2H), 2.93 – 2.80 (m, 2H), 2.37 – 2.27 (m, 2H), 1.80 (s, 3H).[8]

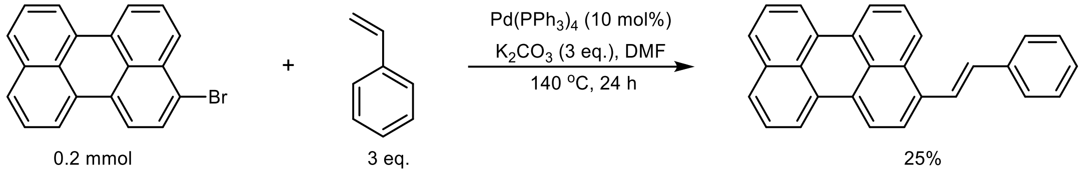

In a glove box, 3-bromoperylene (66 mg, 0.2 mmol), styrene (69 μL, 3 eq.), DMF (2 mL), $Pd(PPh_3)_4$ (23 mg, 10 mol%) and K_2CO_3 (83 mg, 3 eq.) were added to a 10-mL Schlenk tube. The Schlenk tube was sealed and heated at 140 ^{o}C for 24 h. Then, cooled to room temperature, 50 mL ethyl acetate was added, washed with water three times. The organic phase was dried by Na_2SO_4 and the solvent was removed under vacuum. The obtained residue was purified by a silica gel column (PE/EtOAc) to yield the desire product (18 mg, 25%). ^{1}H NMR (400 MHz, DMSO-*d*$_6$) δ 8.44 (d, *J* = 7.6 Hz, 1H), 8.40 (dd, *J* = 8.2, 5.6 Hz, 3H), 8.33 (d, *J* = 8.5 Hz, 1H), 8.04 (d, *J* = 16.1 Hz, 1H), 7.95 (d, *J* = 8.0 Hz, 1H), 7.81 (dd, *J* = 8.2, 2.9 Hz, 4H), 7.63 (t, *J* = 8.0 Hz, 1H), 7.56 (t, *J* = 7.8 Hz, 2H), 7.44 (t, *J* = 7.6 Hz, 2H), 7.38 (d, *J* = 16.1 Hz, 1H), 7.33 (t, *J* = 7.3 Hz, 1H).[9]

Spectra:

Compound **5**

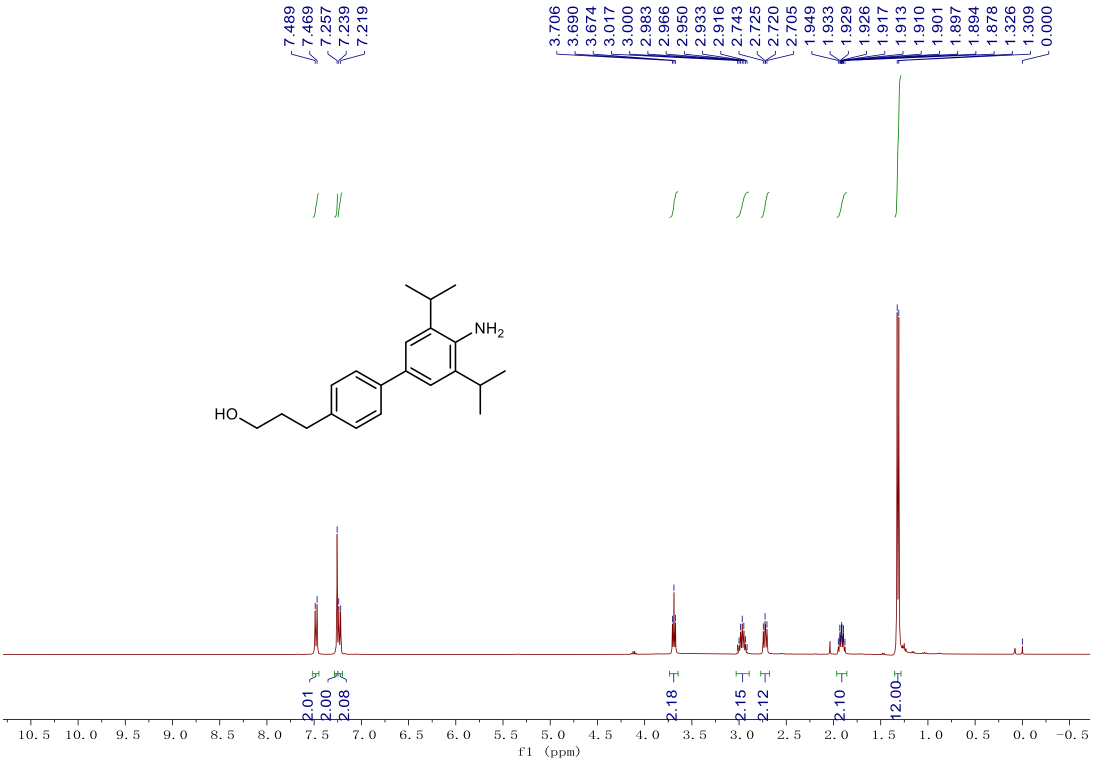

Supplementary Figure 2. ^{1}H NMR of compound 5 in $CDCl_3$.

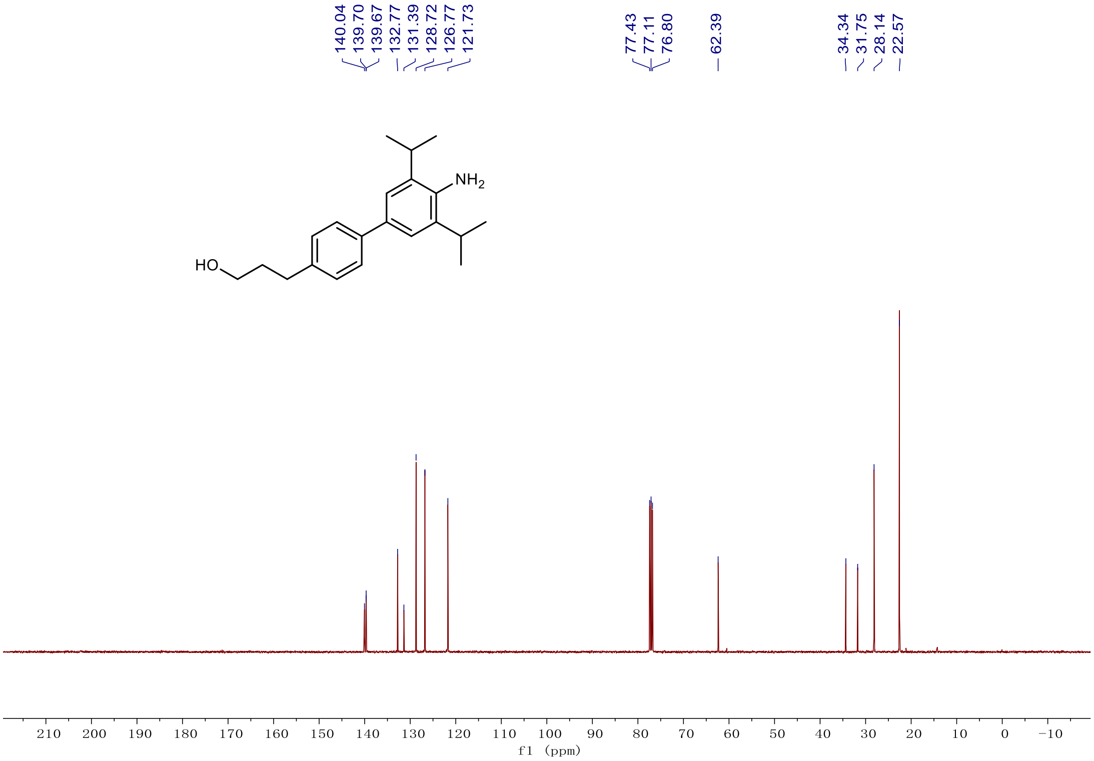

Supplementary Figure 3. ^{13}C NMR of compound 5 in $CDCl_3$.

Compound **6**

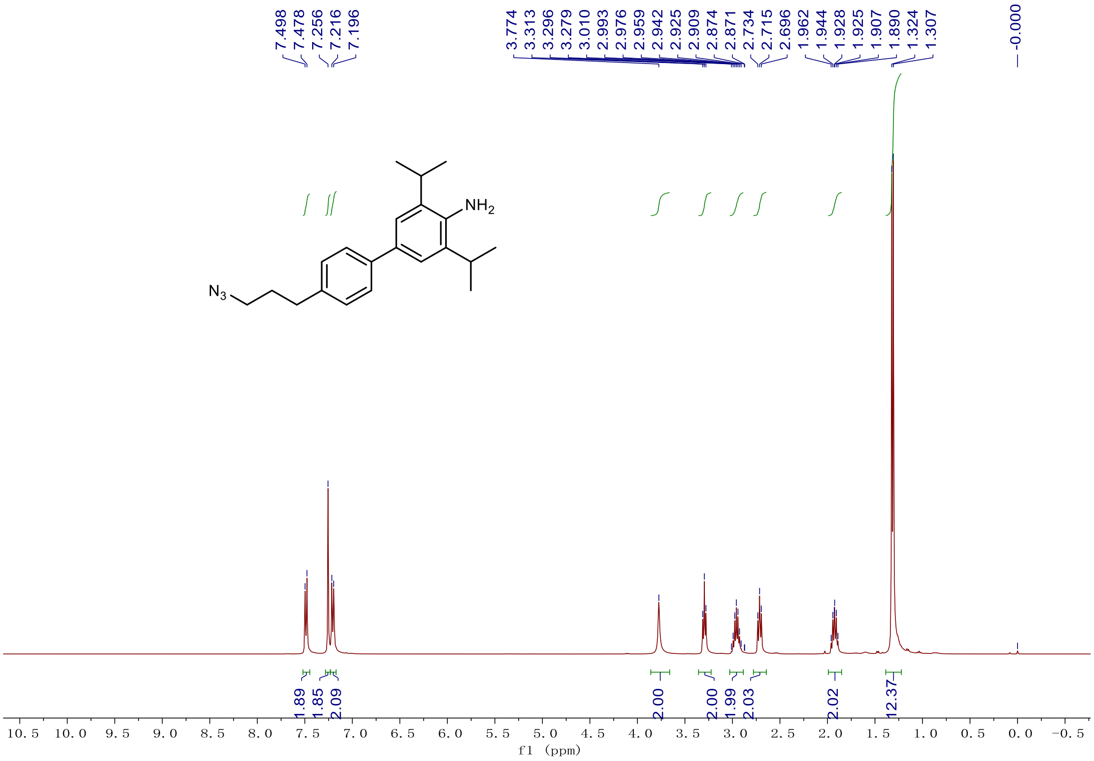

Supplementary Figure 4. ^{1}H NMR of compound 6 in CDCl$_3$.

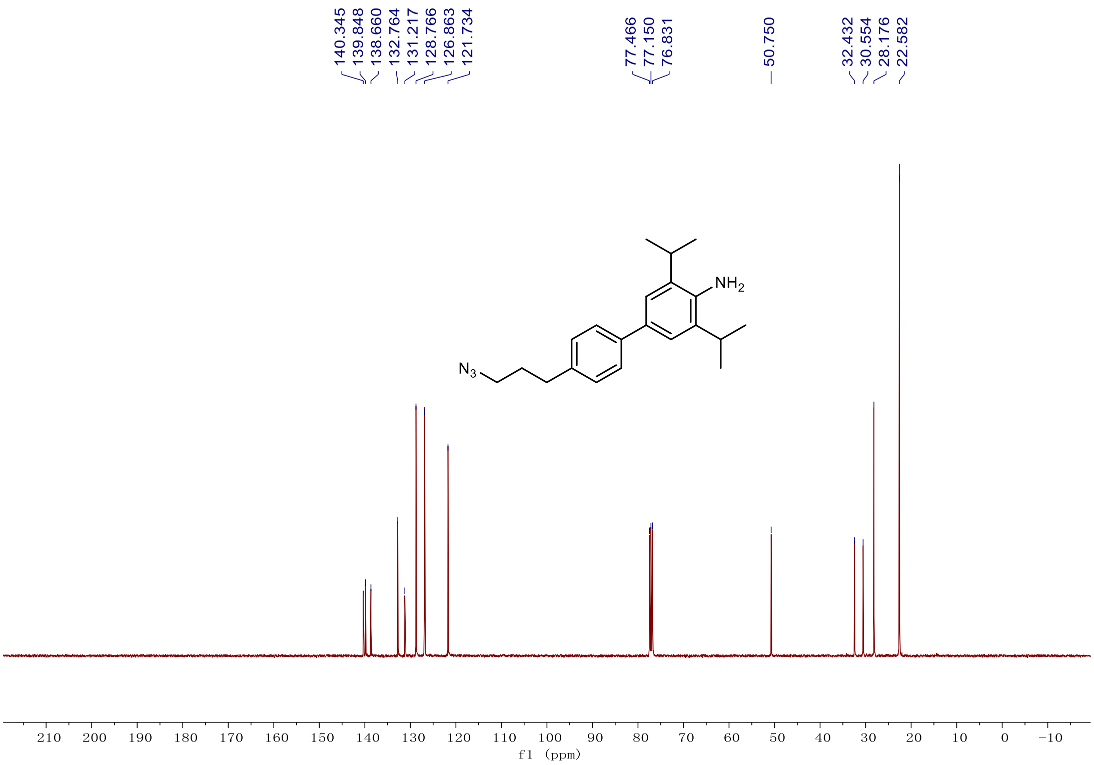

Supplementary Figure 5. ^{13}C NMR of compound 6 in CDCl$_3$.

Compound **7**

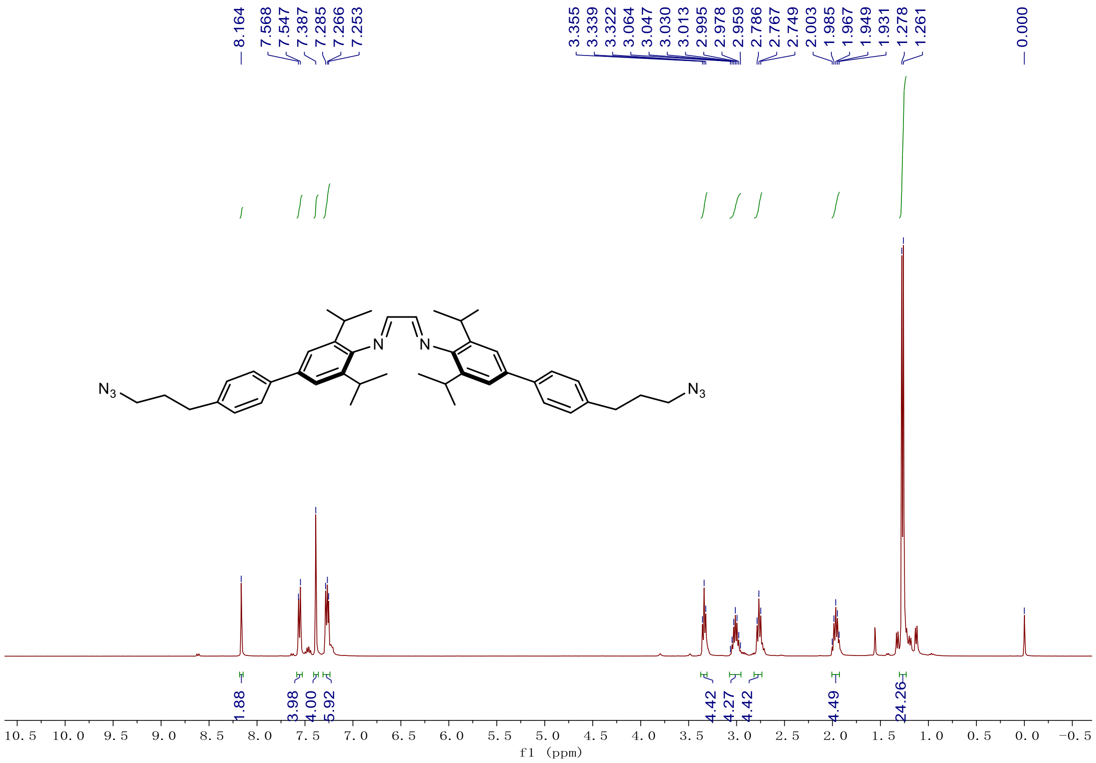

Supplementary Figure 6. ^{1}H NMR of compound 7 in $CDCl_3$.

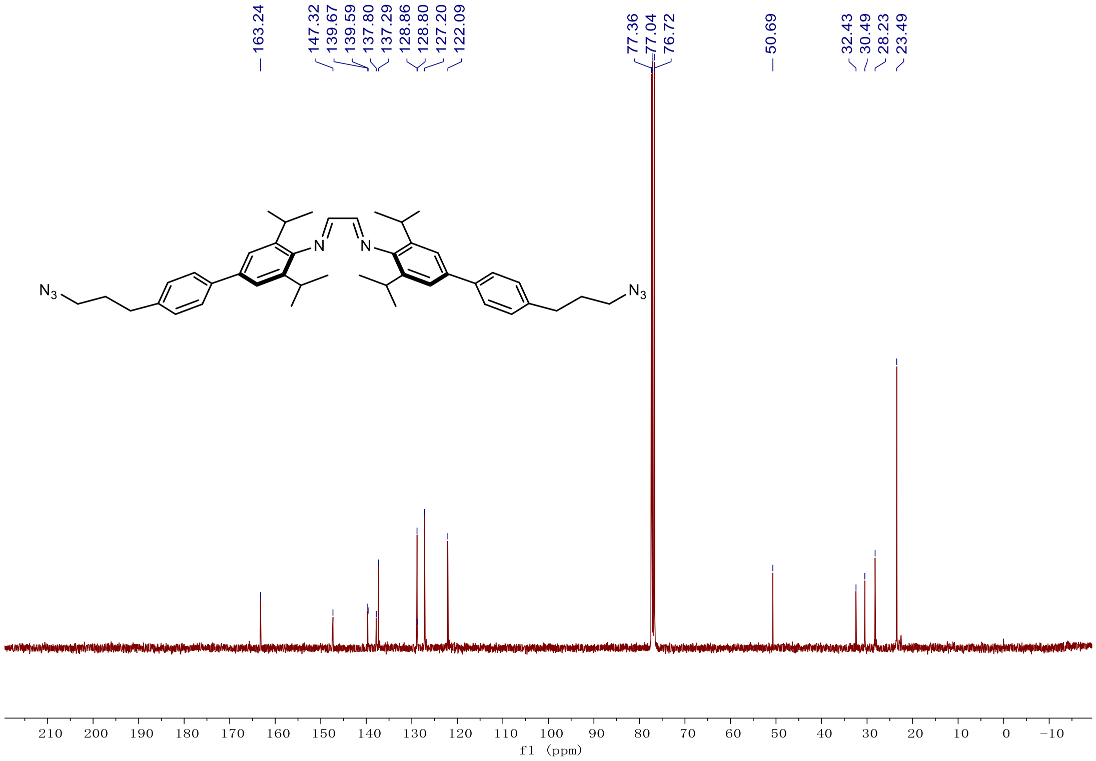

Supplementary Figure 7. ^{13}C NMR of compound 7 in $CDCl_3$.

Compound **8**

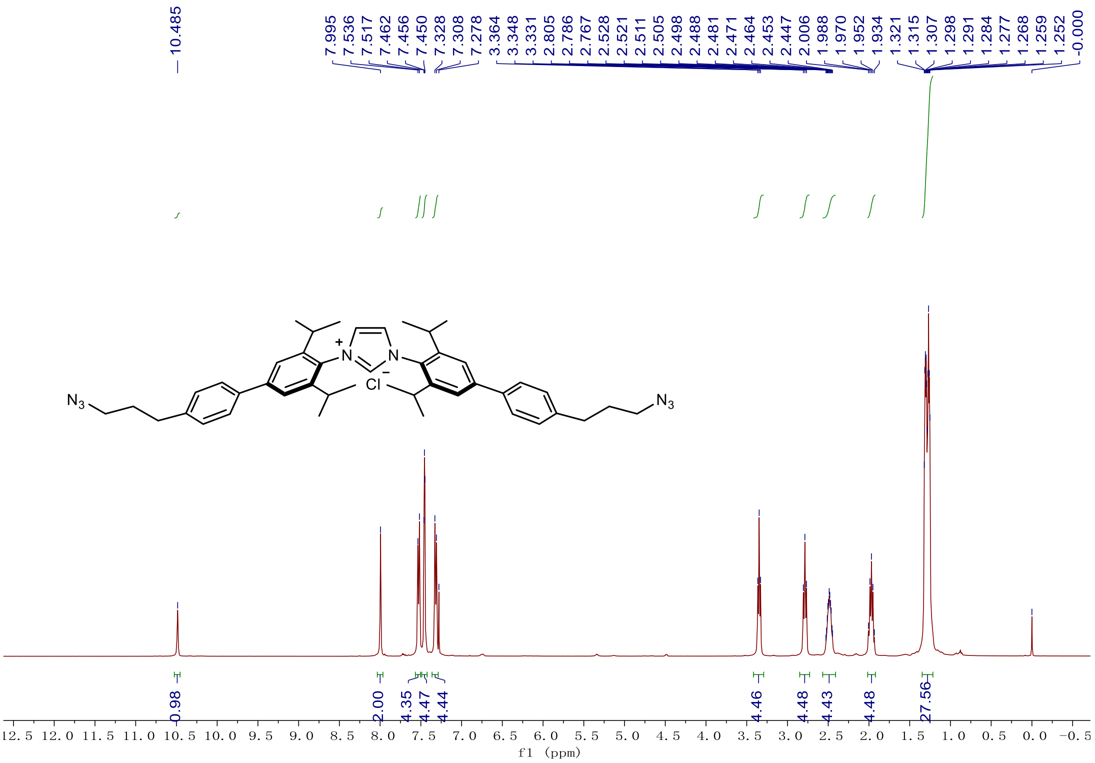

Supplementary Figure 8. ^{1}H NMR of compound 8 in $CDCl_3$.

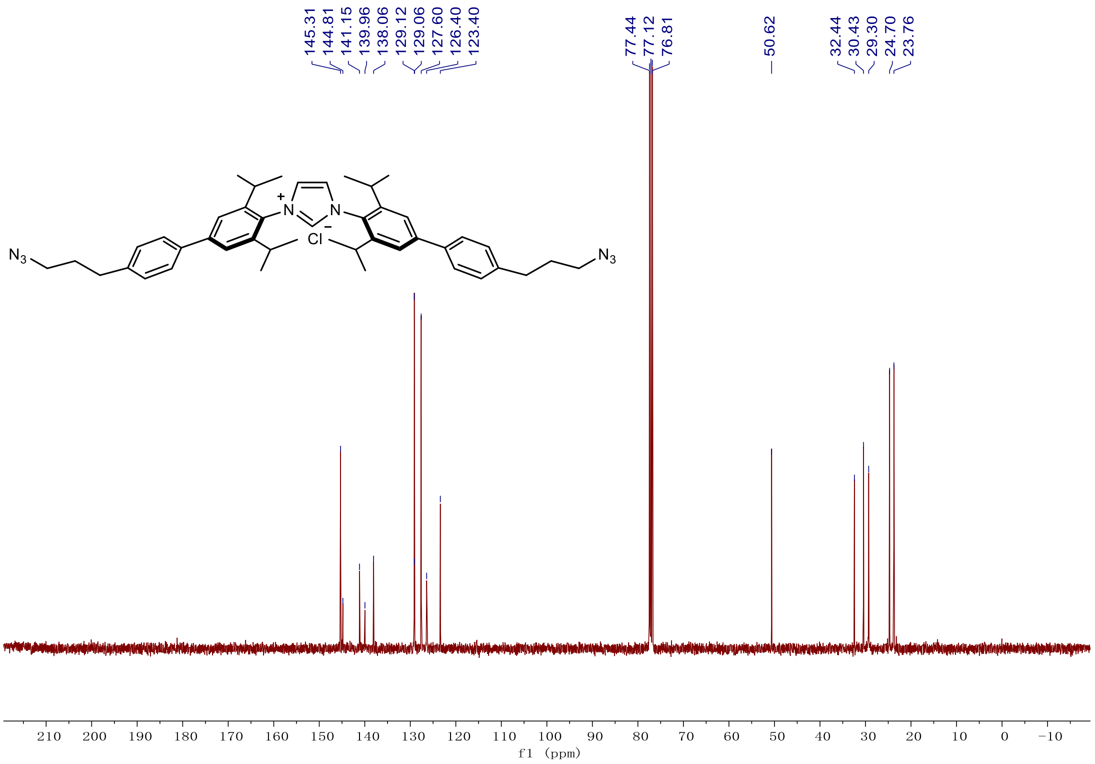

Supplementary Figure 9. ^{13}C NMR of compound 8 in $CDCl_3$.

Compound **9**

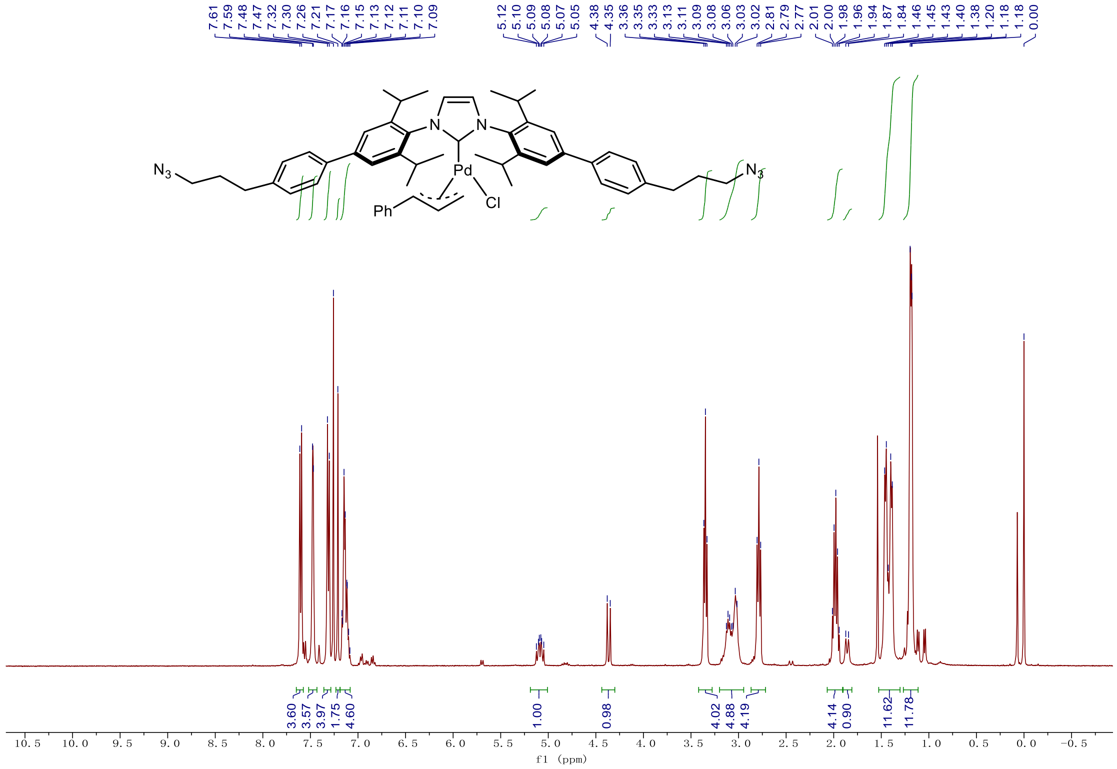

Supplementary Figure 10. ^{1}H NMR of compound 9 in $CDCl_3$.

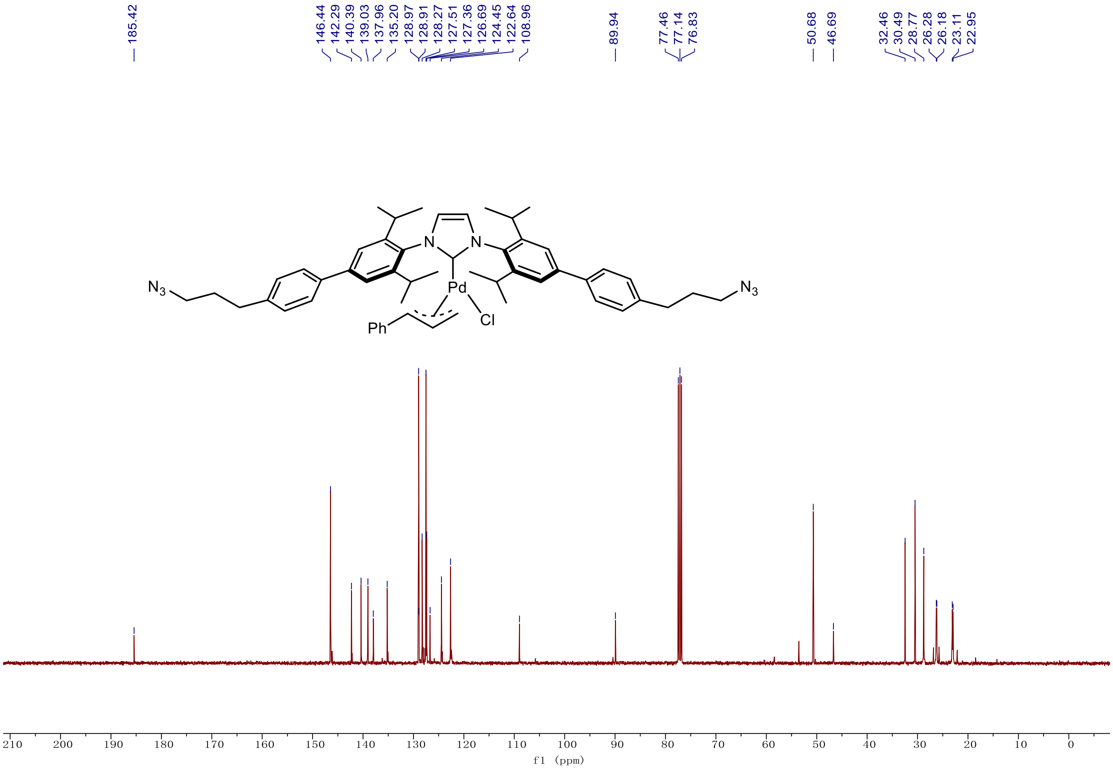

Supplementary Figure 11. ^{13}C NMR of compound 9 in $CDCl_3$.

Other spectra

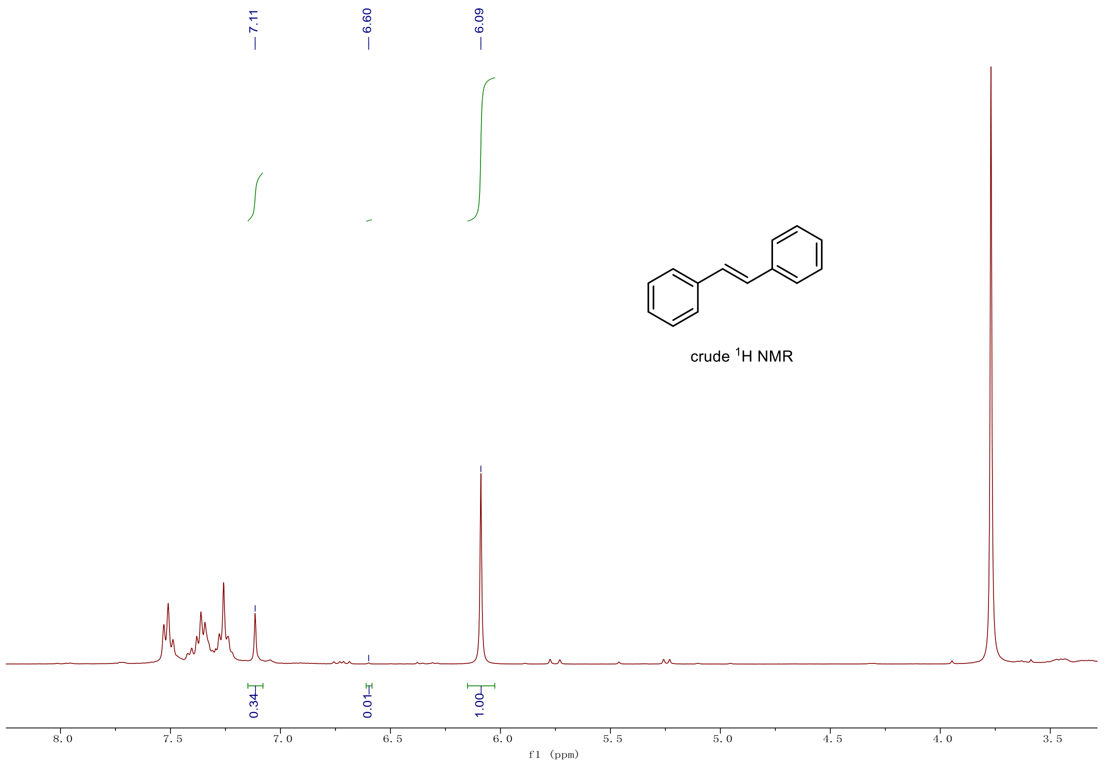

Supplementary Figure 12. ¹H NMR (in $CDCl_3$) of the Mizoroki-Heck cross-coupling reaction product without purification. δ = 7.11, *trans*-Stilbene; δ = 6.60, *cis*-Stilbene; δ = 6.09, 1,3,5-Trimethoxybenzene (internal standard).

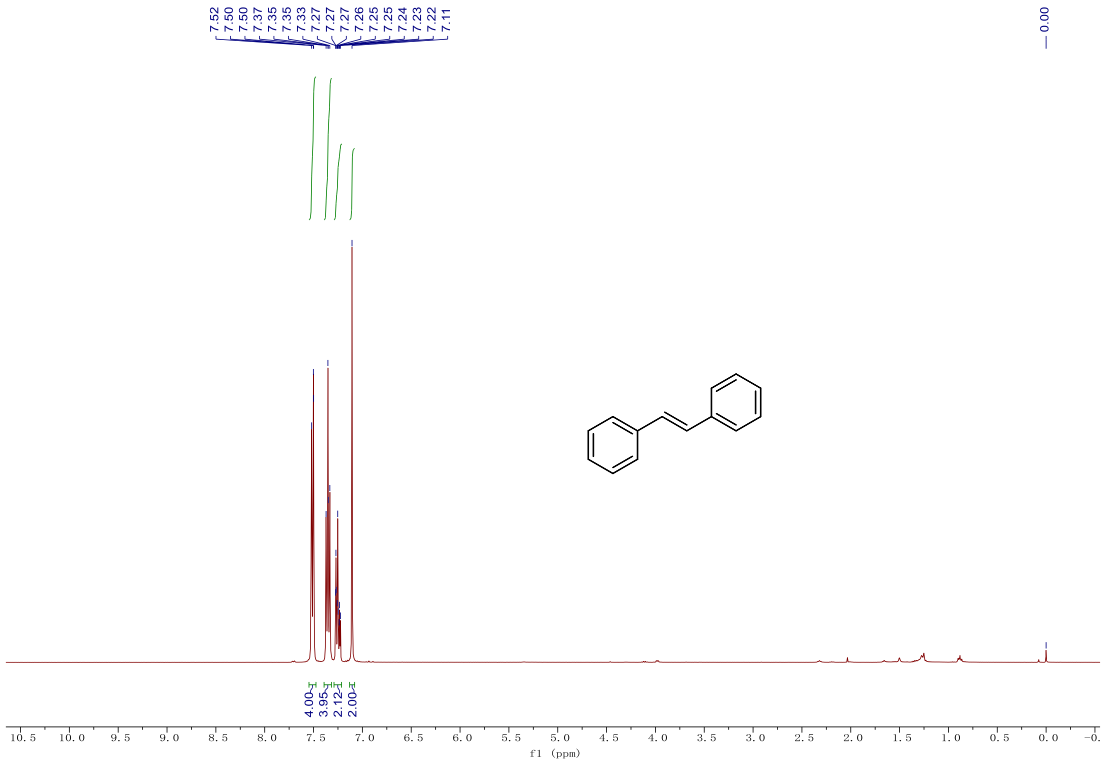

Supplementary Figure 13. ¹H NMR of *trans*-Stilbene in $CDCl_3$ after purification.

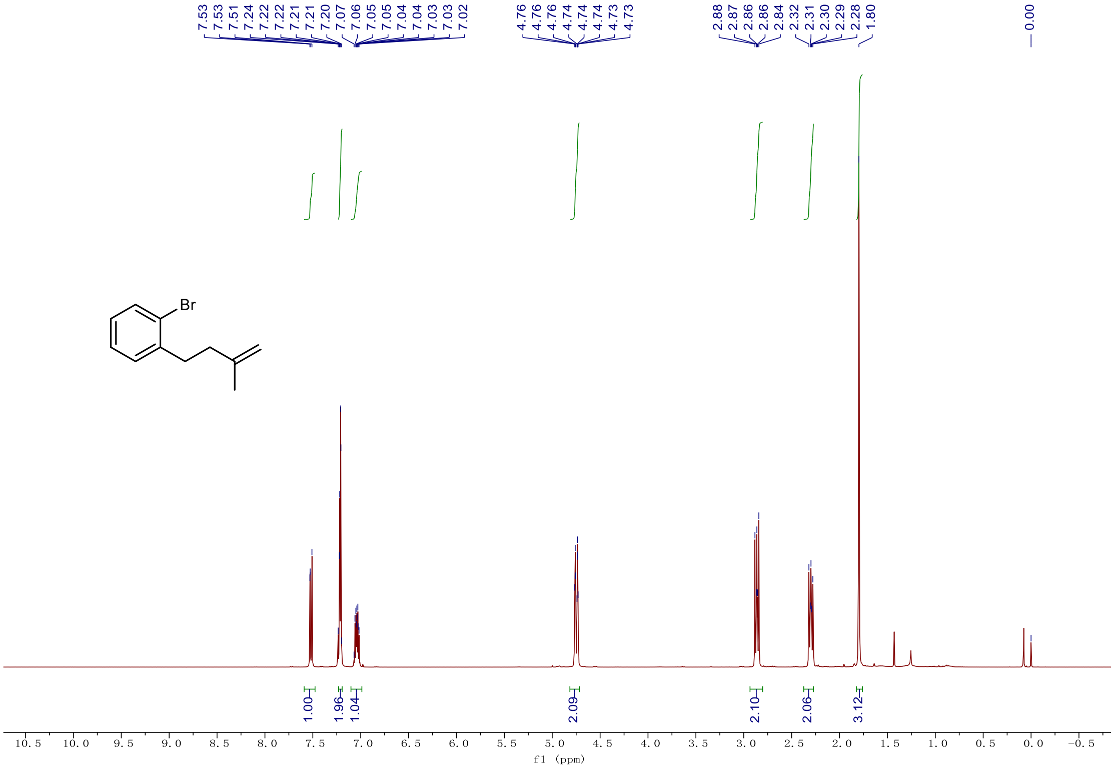

Supplementary Figure 14. ^{1}H NMR of 1-bromo-2-(3-methylbut-3-en-1-yl)benzene in $CDCl_3$.

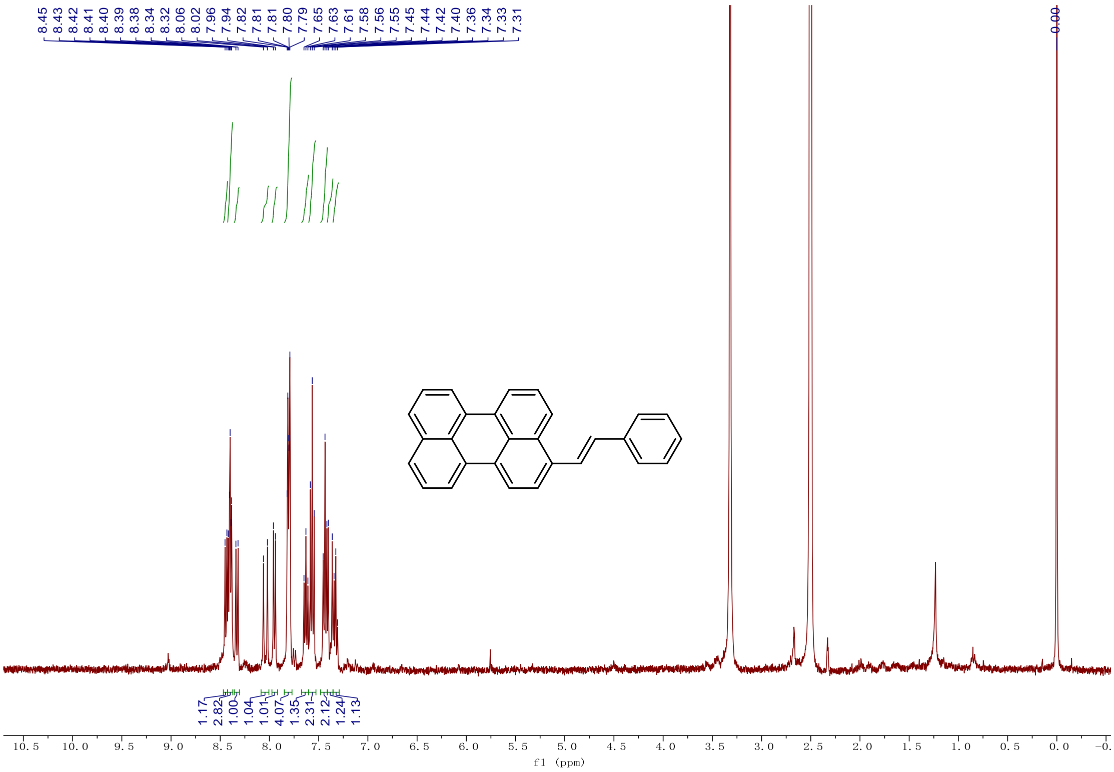

Supplementary Figure 15. ^{1}H NMR of (*E*)-3-styrylperylene in $CDCl_3$.

2. Single-molecule Connection Analysis

I−V curves for 16 connected devices:

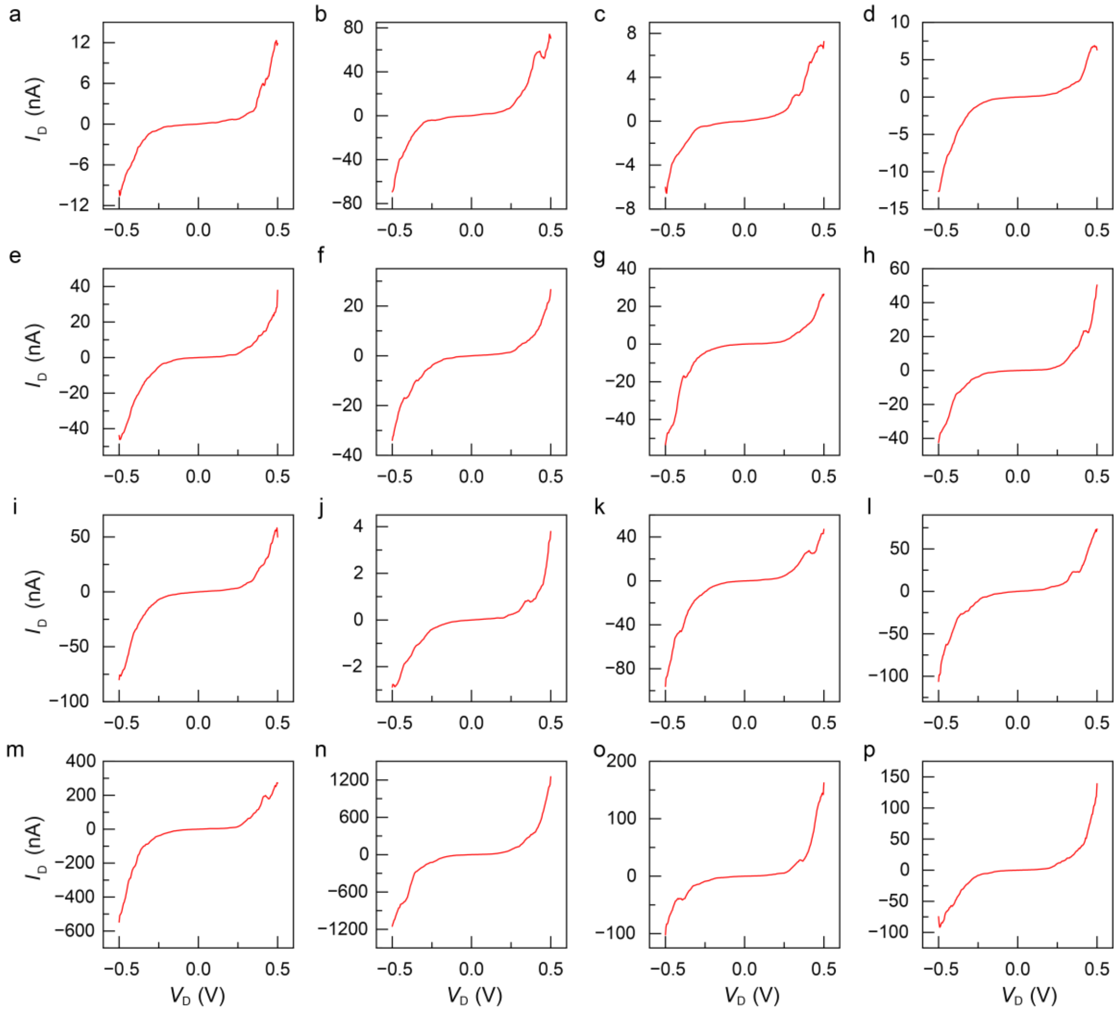

Supplementary Figure 16. Additional single-molecule devices. *I*−*V* curves for 16 connected devices, indicating the reproducibility at 298 K.

Supplementary Note 2. Probability of connecting one molecule

m = ~210 pairs of graphene point electrodes were formed between each pair of gold electrodes. With optimised conditions, the connection yield reached ca. 17 % in real experiments, so the probability of a successful connection between a pair of graphene electrodes is ρ = 17% / 210 = 0.083%. Assuming a binomial distribution, the probability that n molecules are connected to a pair of gold electrodes is G_n:

$$G_n = \frac{m!}{n!\,(m-n)!}\rho^n(1-\rho)^{m-n}$$

The probability of molecules being connected to a pair of gold electrodes is:

$$\gamma_c = 1 - G_0 = 1 - \frac{m!}{0!\,(m-n)!}\rho^0(1-\rho)^m = 1-(1-\rho)^m$$

Thus, the probability of connecting only one molecule is: G_1 / γ_c = ~92%.

This result indicates that there is most likely only one molecular integrated between a pair of successfully connected gold electrodes.

Super high-resolution optical-electrical integrated detector:

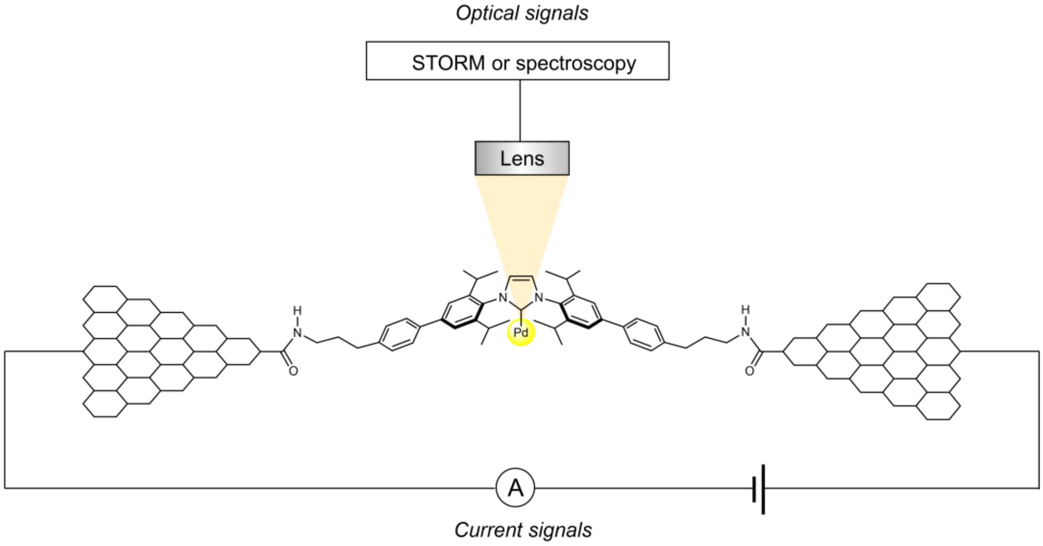

Supplementary Figure 17. Schematic of the superhigh-resolution photo-electrical integrated system. The optical and electrical signals can be recorded synchronously on a self-built super high-resolution optical-electrical integrated detector[10]. STORM: stochastic optical reconstruction microscopy.

Fluorescent super-resolution imaging of the single-molecule catalyst:

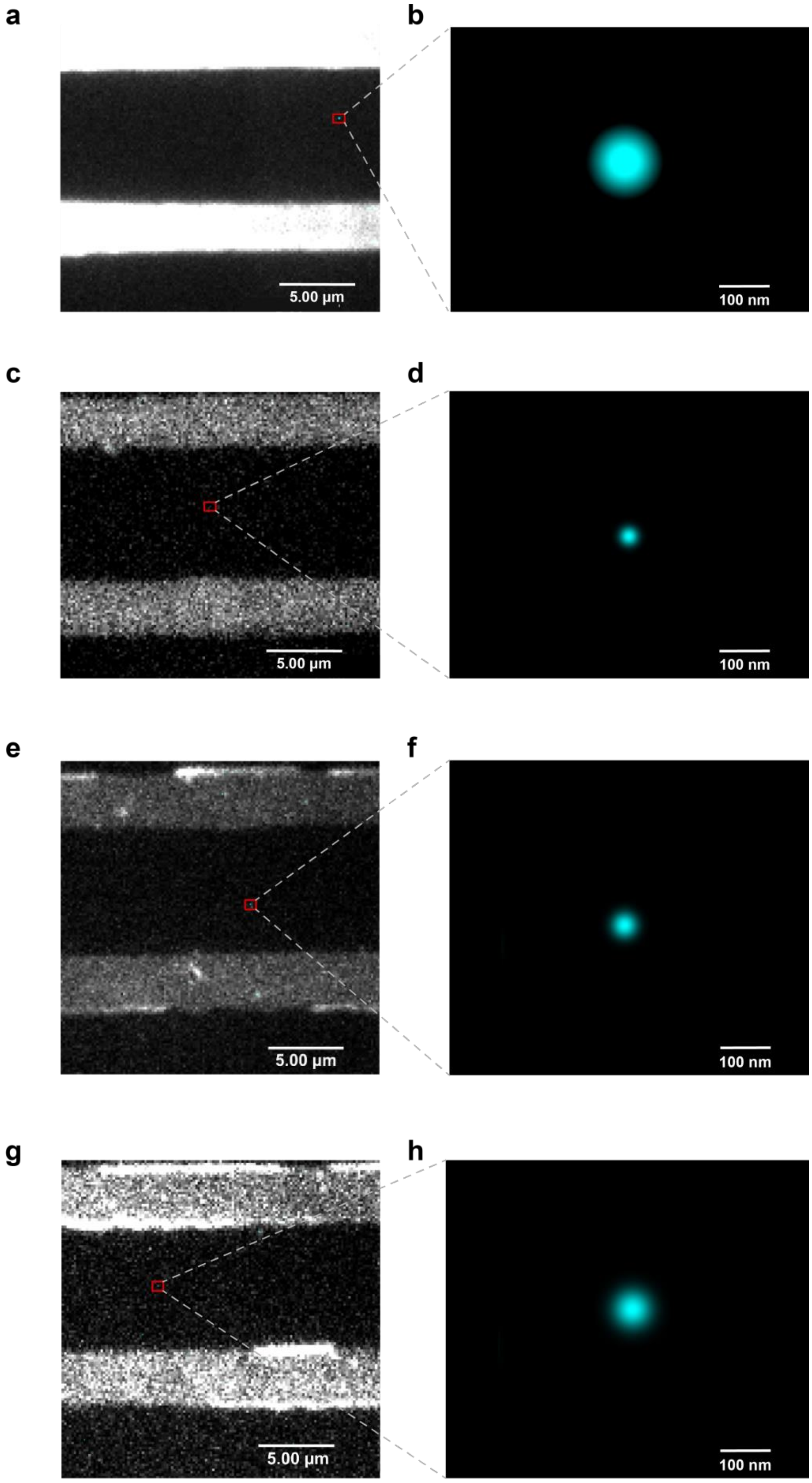

Supplementary Figure 18. Fluorescent imaging photographs for additional single-molecule devices. **a**, **c**, **e** and **g** are mapping images of the devices with the background after STORM. The bright areas indicate the accumulated fluorescence. The proximity of the fluorescent substrate and the accumulation of the fluorescent products during the reaction caused the fluorescence accumulation at a single-molecule site after STORM. **b**, **d**, **f** and **h** are enlarged photographs at the single-molecule site.

3. Machine Learning and Programs for Data Analysis

Supplementary Note 3. Program code and annotations

The number and values of the current levels (Python programs):

```
from sklearn.mixture import GaussianMixture
from scipy.signal import find_peaks
import matplotlib.pyplot as plt
import numpy as np

#import data
data = []
data = [float(l.split()[1]) for l in open("PhBr.txt")]     # the data are from the Mizoroki-
Heck reaction between PhBr and styrene at 298 K

#convert the type of data
origin_x = np.array(data)
origin_x = origin_x.reshape(-1,1)

#frequency analysis
x = np.array(data)
x = np.around(x)
count = np.zeros((300,),dtype = int)
for i in x:
    count[int(i)] += 1
peaks, _ = find_peaks(count,prominence = 1)
print("peaks = ",peaks)     # the result: peaks =   [164 181 194 262]
k = peaks.shape[0]     # the number of k

x = x.reshape(-1,1)
gmm = GaussianMixture(n_components = k,means_init = peaks.reshape(-1,1))
gmm.fit(origin_x)     # training model
label_gmm = gmm.predict(origin_x)
centers = gmm.means_.reshape(k,)
print("GMM center = ",centers)     # the result: GMM center =   [163.84486933
184.71954882 194.03737983 262.4433609 ]
centers = np.around(centers).astype(int)

#plot the result of cluster analysis
plt.subplot(211)
y = np.zeros((x.shape[0],))
plt.xlim(0,300)
plt.title('gmm scatter, k = '+ str(k) )
```

```
plt.scatter(x,y,c = label_gmm)
plt.subplot(212)
plt.xlim(0,300)
plt.plot(centers,count[centers],'xr')
plt.plot(count)
plt.legend(['GMM centers'])
plt.xlabel("$\it{I}$ (nA)")


plt.show()
```

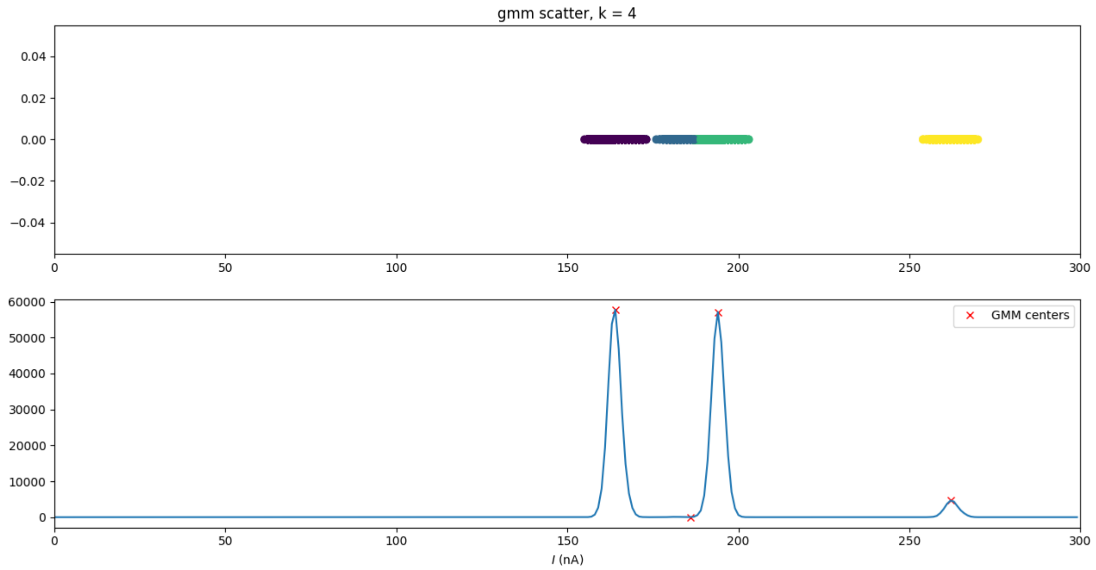

Supplementary Figure 19. The result of cluster analysis via Python program. Gmm: Gaussian mixed model.

Programs (Python) for data analysis:

```
import numpy as np
import matplotlib.pyplot as plt
import csv

# extract data from original data file
file = "./PhBr.txt"      # the data are from the Mizoroki-Heck reaction between PhBr and
styrene at 298 K
t = np.loadtxt(file, delimiter=None, dtype="float")
t1 = t[:, 0]      # extract column 1 (time) in original data file
t2 = t[:, 1]      # extract column 2 (current) in original data file

# idealisation of the current signal
t2[t2 < 175] = 1      # the lowest current level is defined as current level 1
t2[t2 > 253] = 4      # the highest current level is defined as current level 4
```

```
i = 0
while i < 576000:
        if 187 < t2[i] <= 203:
                t2[i] = 3       # the current values between 187 and 203 nA (these values vary at different reaction conditions) are defined as current level 3
        if 175 <= t2[i] <= 187:
                t2[i] = 2       # the current values between 175 and 187 nA (these values vary at different reaction conditions) are defined as current level 2
        i += 1
t1 = list(t1)      # convert t1 to list data
t2 = list(t2)      # conver t2 to list data

# generate current level transformation sequences and record the time position of transformation
t3 = list()          # create new column for t3 (sequences)
change_position = list()      # create new list for the time position of transformation
i = 0
while i < 575999:
        if t2[i] != t2[i+1]:
                t3.append(t2[i])
                change_position.append(i)
        i += 1
t3.append(t2[i])      # record the last current level within the data, assign as t3
t3 = list(map(int, t3))    # convert t3 to integer data
print(t3)     # print the current level transformation sequences
print(len(t3))      # print the length of t3 (the time position of current level transformation: t3/57600)
print(change_position)      # print the time position of transformation
# the sequences and the time position of transformation can be used to analyze the dwell time and kinetics of Mizoroki-Heck reaction


# Search specific current transformation and can be used for reversibility study
A = [1, 3, 4, 2, 1]      # set a sequence (any sequence can be set)
i = 0
a = 0
for i in range(0, len(t3)-len(A)+1):      # if the sequence exist, it will be counted as a
        if A == t3[i:i+len(A)]:
                a += 1
print('There are', a, ' sequences of 13421.')

# Record specific current transformation
A = [1, 3, 4, 2, 1]      # set a sequence (any sequence can be set)
A_start_position = []
```

```
A_end_position = []
a = 0
for i in range(0, len(t3)-len(A)+1):      # if the sequence exist, it will be counted as a
    if A == t3[i:i+len(A)]:
        a += 1
        A_start_position.append(change_position[i])
        A_end_position.append(change_position[i+len(A)])

# extract corresponding original data of the specific sequence, and record in csv. file.
t2 = t[:, 1]      # extract column 2 (current signals)
for i in range(a):
    b = A_end_position[i] - A_start_position[i]
    b = int(b)
    filename = 'Br_13421_' + str(i+1) + '.csv'
    csvFile = open(filename, 'w', newline='')
    try:
        writer = csv.writer(csvFile)
        for j in range(int(b+57599*0.02)):
            writer.writerow((t1[int(A_start_position[i] - 57600*0.01 + j)],
t2[int(A_start_position[i]-57600*0.01+j)]))
    finally:
        csvFile.close()
```

4. Experiments for the Attribution of Different Current Levels

The reversibility of different current levels:

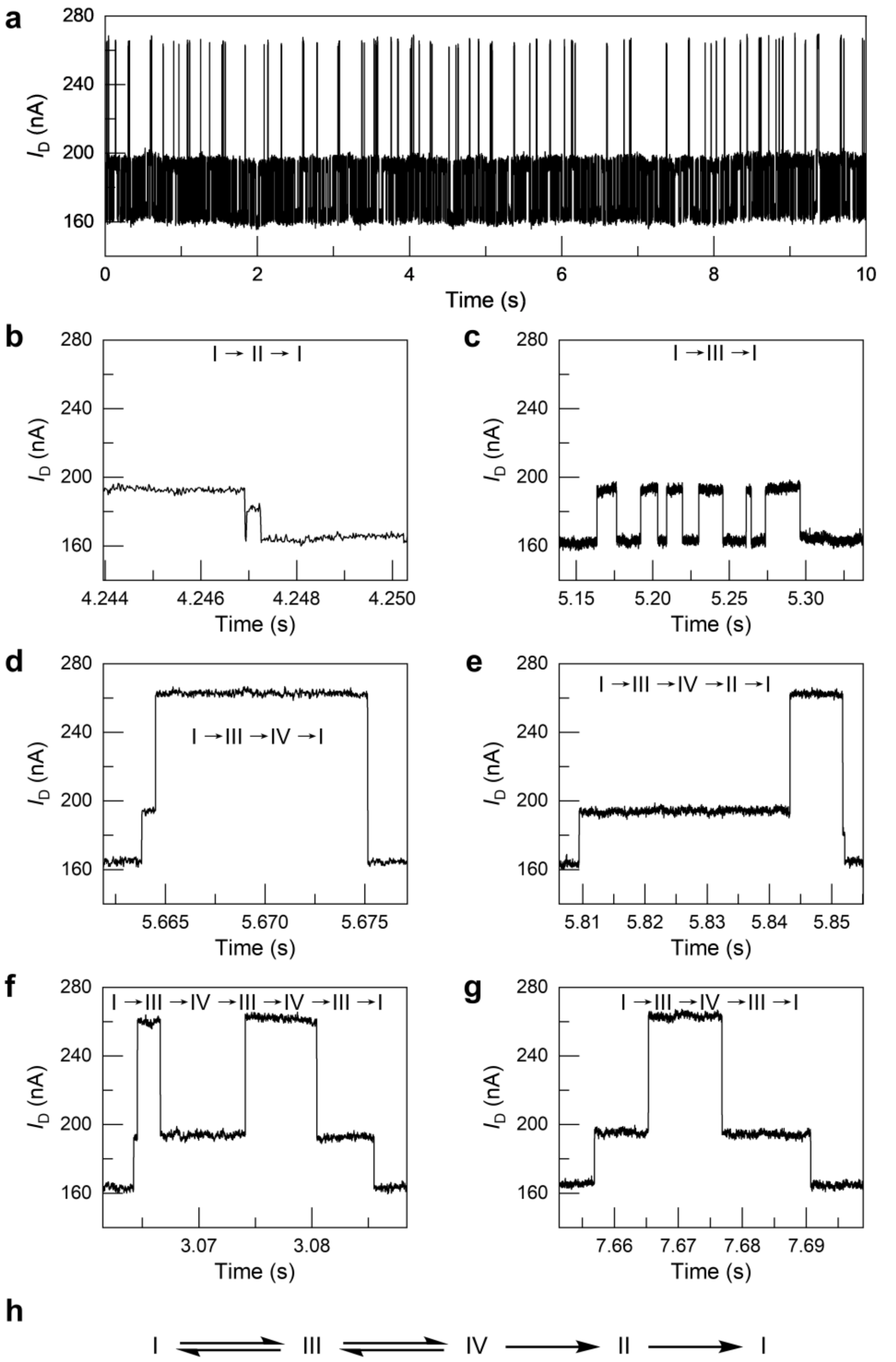

Supplementary Figure 20. The reversibility of different current levels for the single-molecule Mizoroki-Heck cross-coupling between styrene and bromobenzene at 300 mV and 298 K. a. The monitored current signal for 10 s. **b**. Current transformation: I to II to I. These processes are attributed as oxidative addition of HBr to Pd(0) and its reverse step. **c**. Current transformation: I to III to I. These processes are attributed as oxidative

addition of PhBr to Pd(0) and its reverse step. **d**. Current transformation: I to III to IV to I. These processes are attributed as oxidative addition of PdBr to Pd(0), olefin coordination and reverse step of oxidative addition / olefin dissociation. **e**. Current transformation: I to III to IV to II to I. These processes are attributed as oxidative addition of PdBr to Pd(0), olefin coordination, olefin insertion and β-H elimination/reductive elimination. **f**. Current transformation: I to III to IV to III to IV to III to I. These processes are attributed as oxidative addition of PdBr to Pd(0), olefin coordination, olefin dissociation, olefin coordination, olefin dissociation and reverse step of oxidative addition. **g**. Current transformation: I to III to IV to III to I. These processes are attributed as oxidative addition of PdBr to Pd(0), olefin coordination, olefin dissociation and reverse step of oxidative addition. **h**. The analysis result of the reversibility of different current levels. When the gate voltage is applied to elementary steps of an ongoing catalytic cycle, oxidative addition and olefin coordination are still reversible, whereas the reversibility of olefin insertion depends on the magnitude of the gate voltage (see Supplementary Figures 30, 38 and 42−45).

Stepwise experiments for attribution of different current levels:

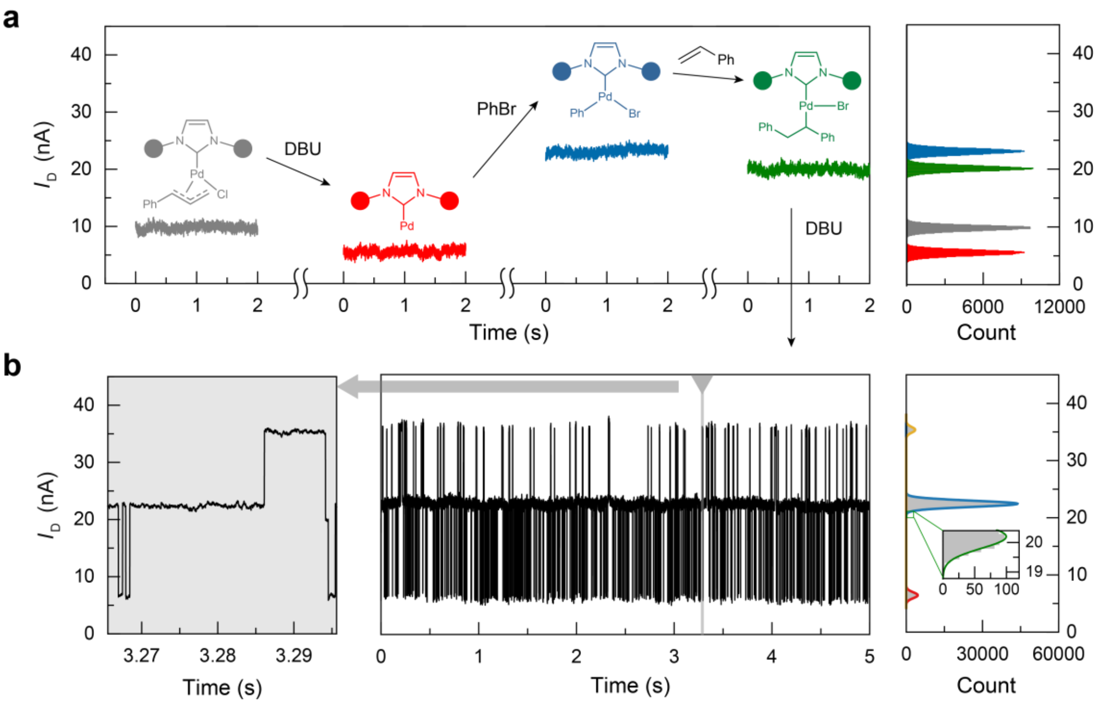

Supplementary Figure 21. Signal transformation of the single-molecule Mizoroki-Heck cross-coupling between styrene and bromobenzene via adding substrates/reagents successively. **a**. Left: Current levels during the stepwise experiments and the corresponding current levels. Right: The corresponding frequency distributions of the current signals at the left figure. DBU: 1,5-diazabicyclo(5,4,0)undec-5-ene. **b**. Middle: The current signal transformation after adding DBU. Left: A catalytic cycle (enlarged figure from the middle). Right: the corresponding attributions of the middle.

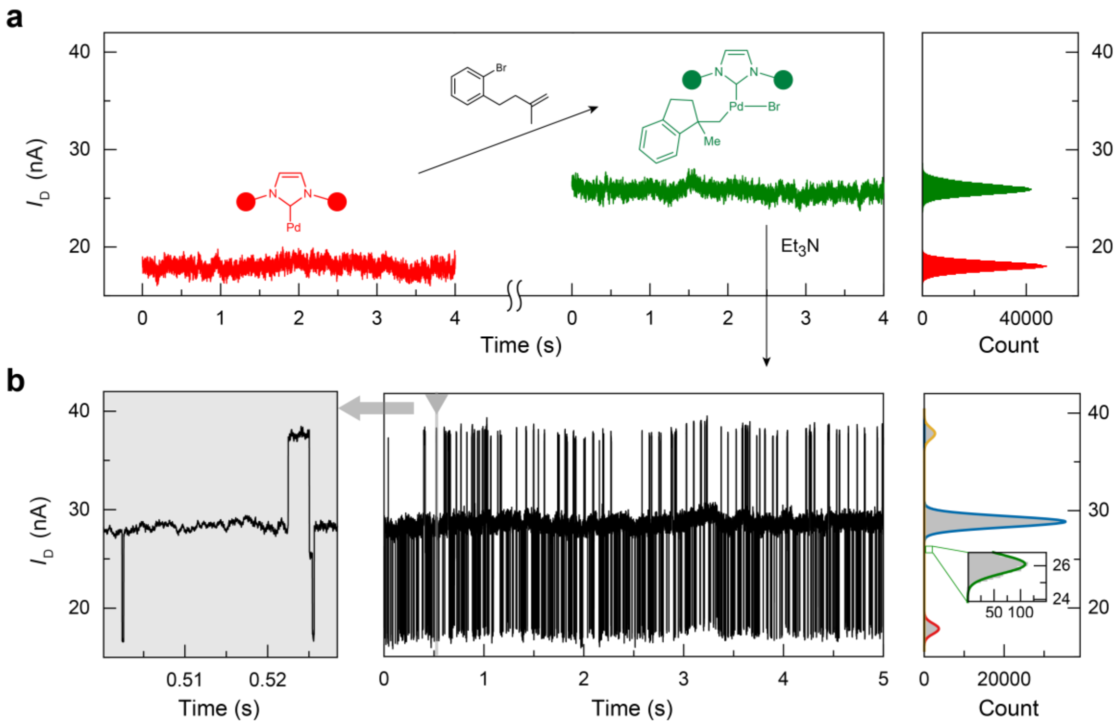

Supplementary Figure 22. Signal transformation of the single-molecule Mizoroki-Heck cross-coupling between styrene and 1-bromo-2-(3-methylbut-3-en-1-yl)benzene via adding substrates/reagents successively. a. Left: Current levels during the stepwise experiments and the corresponding current levels. Right: The corresponding frequency distributions of the current signals at the left figure. **b**. Middle: The current signal transformation after adding Et_3N. Left: A catalytic cycle (enlarged figure from the middle). Right: the corresponding attributions of the middle.

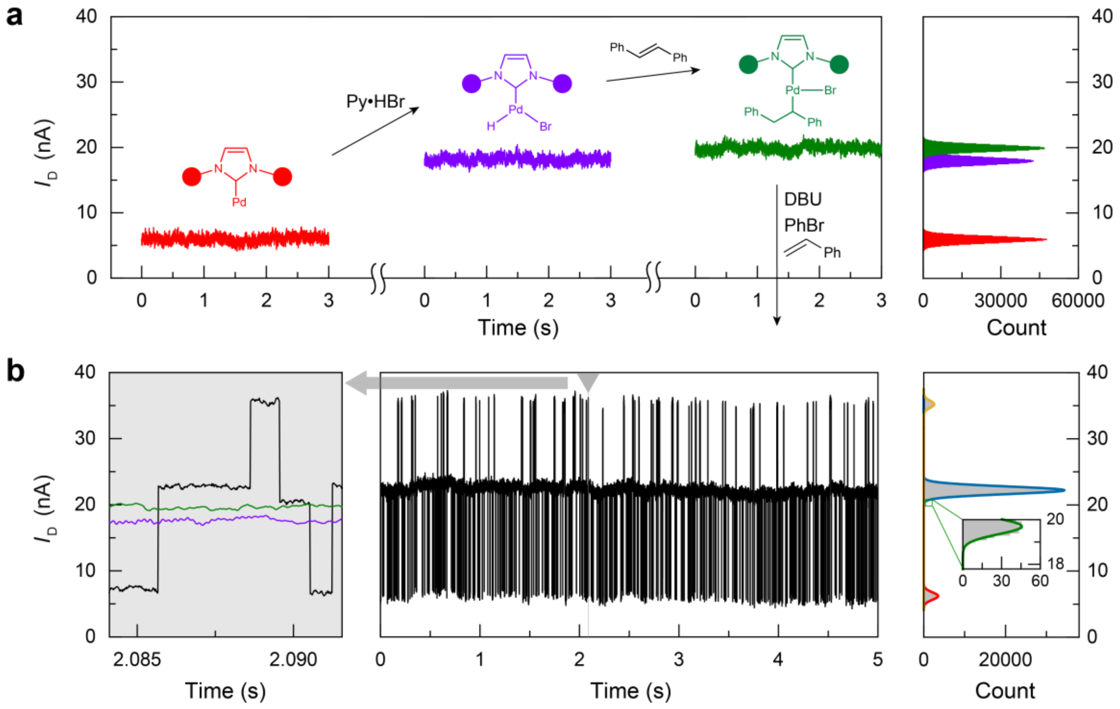

Supplementary Figure 23. Signal transformation of the single-molecule Mizoroki-Heck cross-coupling via adding substrates/reagents successively. a. Left: Current levels during the stepwise experiments and the corresponding current levels. Right: The corresponding frequency distributions of the current signals at the left figure. **b**. Middle: The current signal transformation after removing Py·HBr/*trans*-stilbene and adding DBU, PhBr as well as styrene. Left: A catalytic cycle (enlarged figure from the middle), the current level which is attributed to oxidative addition product of HBr to Pd(0) (purple) and the current level which is attributed to olefin insertion intermediate (green). Right: the corresponding attributions of the middle. Py: pyridine.

Mizoroki-Heck catalytic cycle:

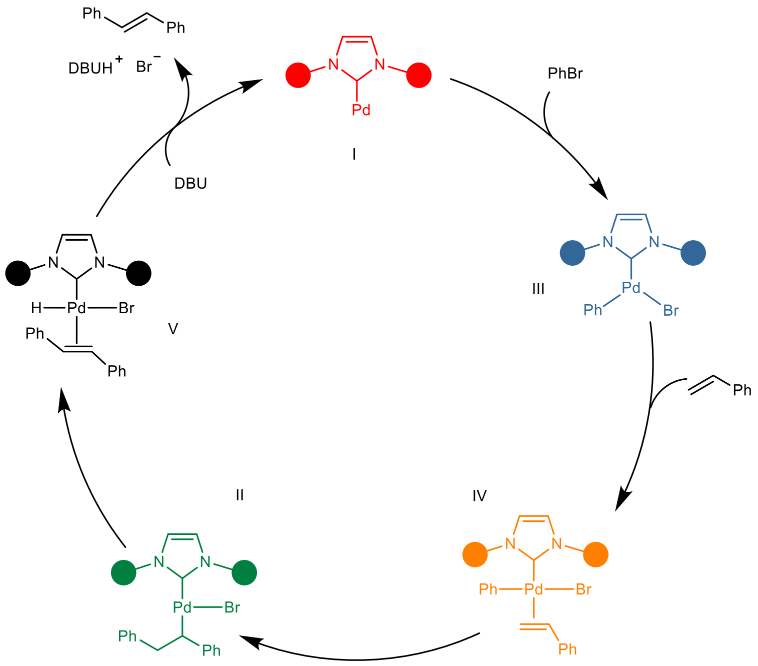

Supplementary Figure 24. Five intermediates within Mizoroki-Heck catalytic cycle and corresponding current level numbers.

5. Theoretical Calculation

Methods:

All the structures were optimised at the B3LYP/6-31G(d)-LANL2DZ[11–14] level of theory. Frequency calculations were performed to verify that intermediates have no imaginary frequency while the transition structures have only one imaginary frequency. Single point energy calculations were carried out at M06L/6-311++G(2d,p)-SDD[15–16] level of theory. The SMD solvation model[17] of DMF was included in both geometric optimization and single point energy calculations for the condition without external electric fields (EEFs). The reported Gibbs free energies were calculated at 298.15K and 1 M. All the calculations were performed with Gaussian 09 software[18].

For the condition in 1-butyl-3-methylimidazolium tetrafluoroborate with EEFs, the ionic liquid solvent in SMD solvation model[19] was defined with the solvent descriptors: dielectric constant (ε) = 11.70[20]; square of refraction (n^2) = 2.0207[21]; Abraham's hydrogen bond acidity parameter ($\Sigma\alpha_2^{\mathrm{H}}$) = 0.263[19]; Abraham's hydrogen bond basicity parameter ($\Sigma\beta_2^{\mathrm{H}}$) = 0.320[19]; the macroscopic surface tension (γ) = 67.07[22]; the fraction of non-hydrogen atoms that are aromatic carbon atoms (ϕ) = 0.20; the fraction of non-hydrogen atoms that are electronegative halogen atoms (ψ) = 0.27. The orientation of external electric fields was set along Pd-C bond between Pd and the NHC ligand (see below Supplementary Figure 26) with the field strength Ez = +0.002 a.u. (about +2 V/nm) or Ez = –0.002 a.u. (about -2 V/nm).

DFT calculation without external electric fields:

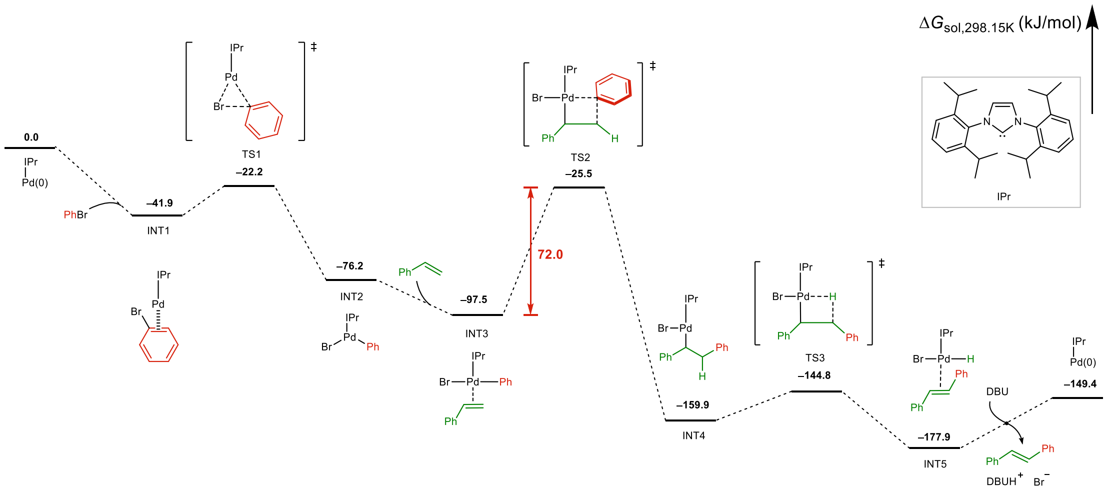

Supplementary Figure 25. DFT calculation results in DMF. IPr: 1,3-bis(2,6-diisopropylphenyl)-1*H*-imidazol-3-ium-2-ide; TS: transition state; INT: intermediate.

DFT calculation under external electric fields:

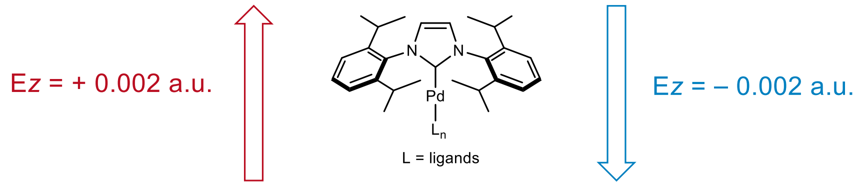

Supplementary Figure 26. The applied external electric fields for DFT calculation.

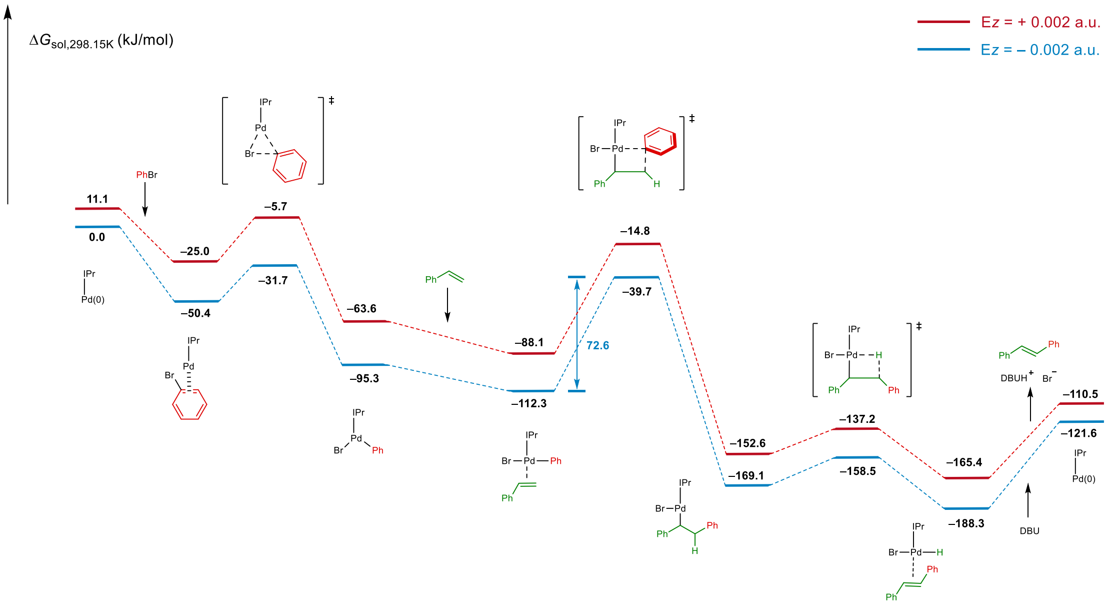

Supplementary Figure 27. DFT calculation results with external electric fields along Pd–C.

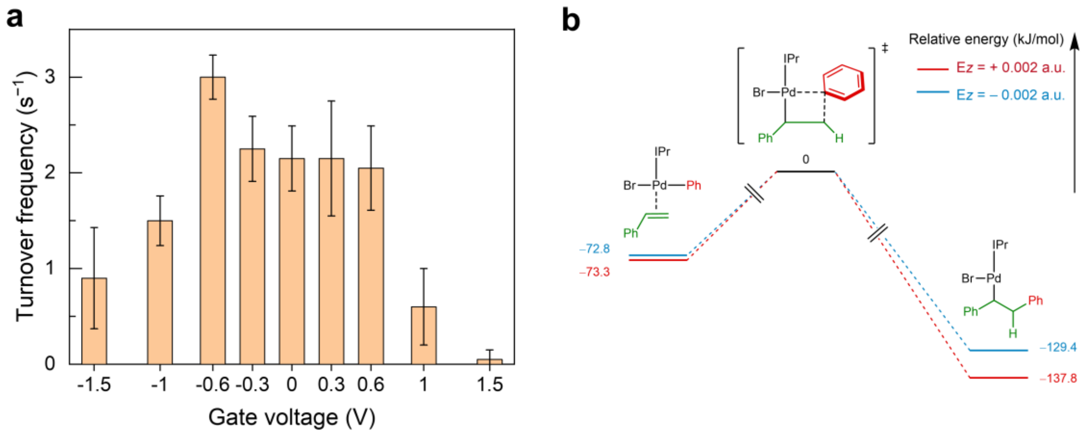

Supplementary Figure 28. The tuning effect of the TON and relative energy for the olefin insertion step under different reaction conditions. a. The tuning effect of the whole Mizoroki-Heck reaction under different gate voltages. **b**. The relative energy of the olefin insertion step. Under mild electric field (−0.002 a.u. < Ez < 0.002 a.u.), the rate-determining step is olefin insertion. The more negative electric field is beneficial for the whole catalytic cycle (for example, the TON is higher under −0.6 V gate voltage compared with other TONs) based on the energies.

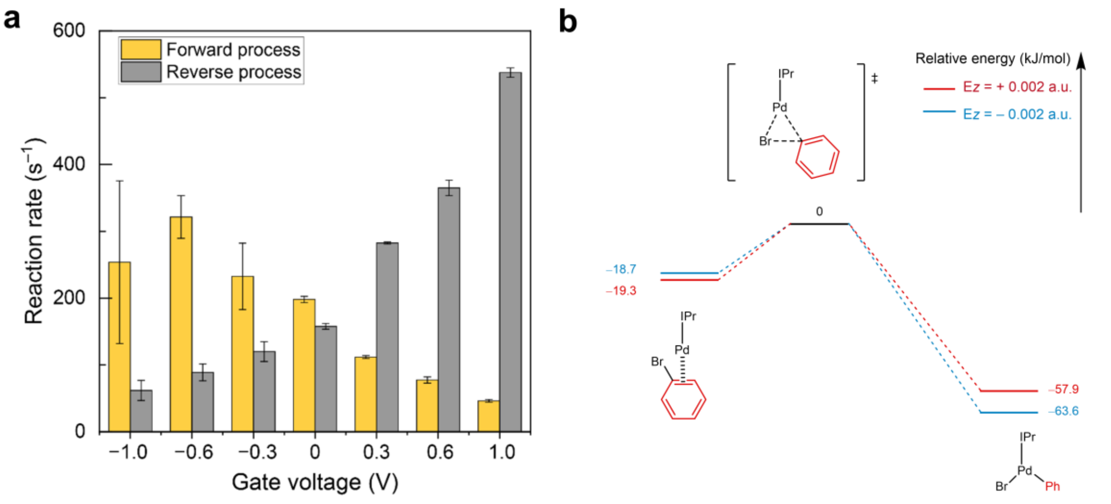

Supplementary Figure 29. The tuning effect of oxidative addition step (forward and reverse processes) and relative energy under different reaction conditions. a. The tuning effect of oxidative addition process (reaction between Pd(0) and bromobenzene) and its reverse process. **b**. The relative energy of the oxidative addition step. When the external electric field is in an opposite orientation of Pd→NHC, the oxidative addition is favored in energy.

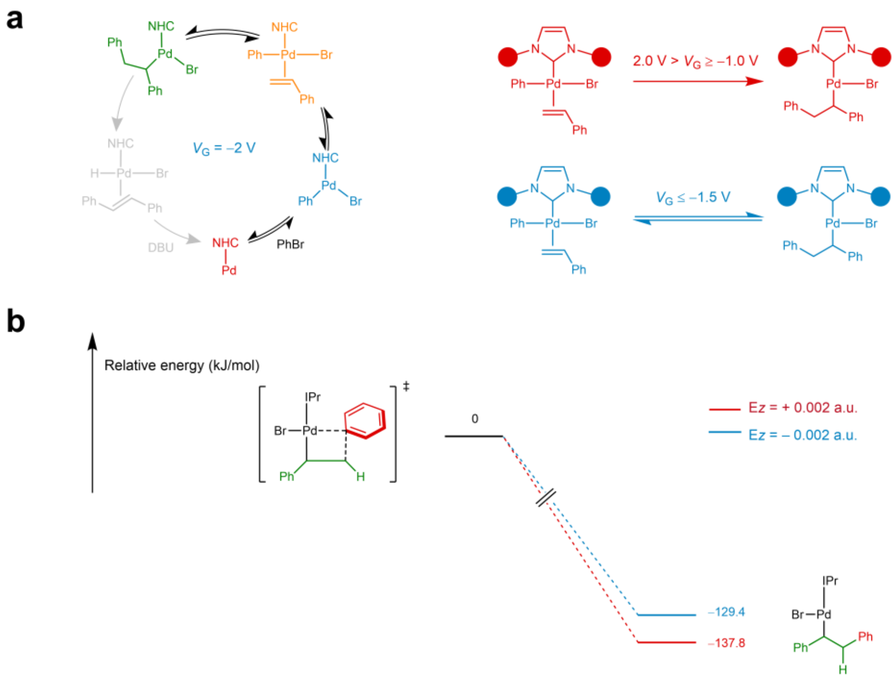

Supplementary Figure 30. The tuning effect of the reverse olefin insertion step and relative energy under different reaction conditions. a. The influence of −2 V gate voltage on the catalytic cycle. When the gate voltages are bigger than −1.5 V, the olefin insertion step is irreversible. Under −2 V and −1.5 V gate voltages, the olefin insertion step becomes reversible. **b.** Relative energy for reverse olefin insertion step. Negative gate voltages would reduce the barrier of the reverse step.

Transmission spectra calculation:

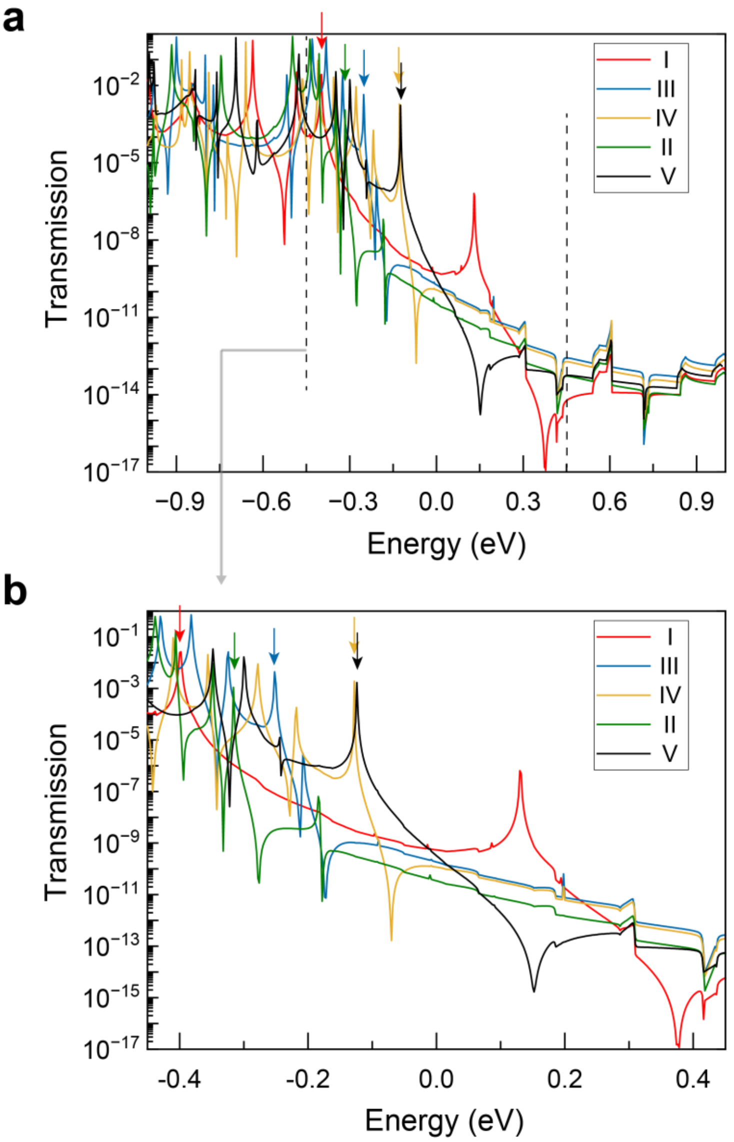

Supplementary Figure 31. Transmission spectra of four intermediates. a. Full transmission spectra of five intermediates. The transport device model was constructed by three parts, i.e. left electrode, central region and right electrode. Both electrodes were semi-infinite heavily p-type doped graphene (0.009 holes per carbon). The central region consisted of the extended molecule and one electrode extension on both sides. The structures of the transport devices were optimised using the B3LYP/6-31G(d)-LANL2DZ level of theory with Gaussian 09 software. The electronic transport simulations were performed by employing the real-space NEGF techniques[23–24] with GGA-PBE functional[25] and PseudoDojo pseudopotential, as implemented in the QuantumATK software (version: R-2020.09). The used basis sets were Medium for hydrogen, carbon and boron, High for nitrogen and oxygen, and Ultra for Palladium, respectively. The density mesh cutoff was set to be 125 Ha. A k-point mesh of 2×1×111 was used for device self-consistent calculations. The OzakiContour method is used for complex contour integration. The transmission spectra are calculated self-consistently for the bias voltage of 0.3 V with a k-point mesh of 12×1, based on which the currents under bias 0.1, 0.2 and 0.3 V are

evaluated. The energy resolution for transmission spectra is set to be as high as 0.002 eV. The solvent effect is included. **b.** Enlarged transmission spectra of the five intermediates.

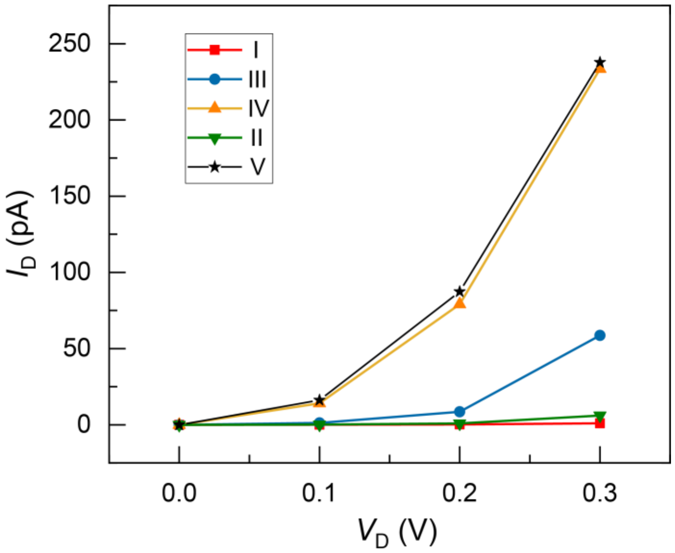

Supplementary Figure 32. Calculated *I*−*V* curves of different intermediates. The responses of the five intermediate currents to the bias voltage are consistent with experimental results. The current level V, which corresponds to the intermediate of β-H elimination (Supplementary Figure 24), has not been observed during the monitoring. The intermediates of current levels IV and V have the similar structure (olefin coordination) and current levels IV and V have the similar relative conductance according to the calculation. There is no high current level behind current II. According to the reaction trajectory, the appearance of current level V can be excluded.

Different haloarenes involved Mizoroki-Heck reactions:

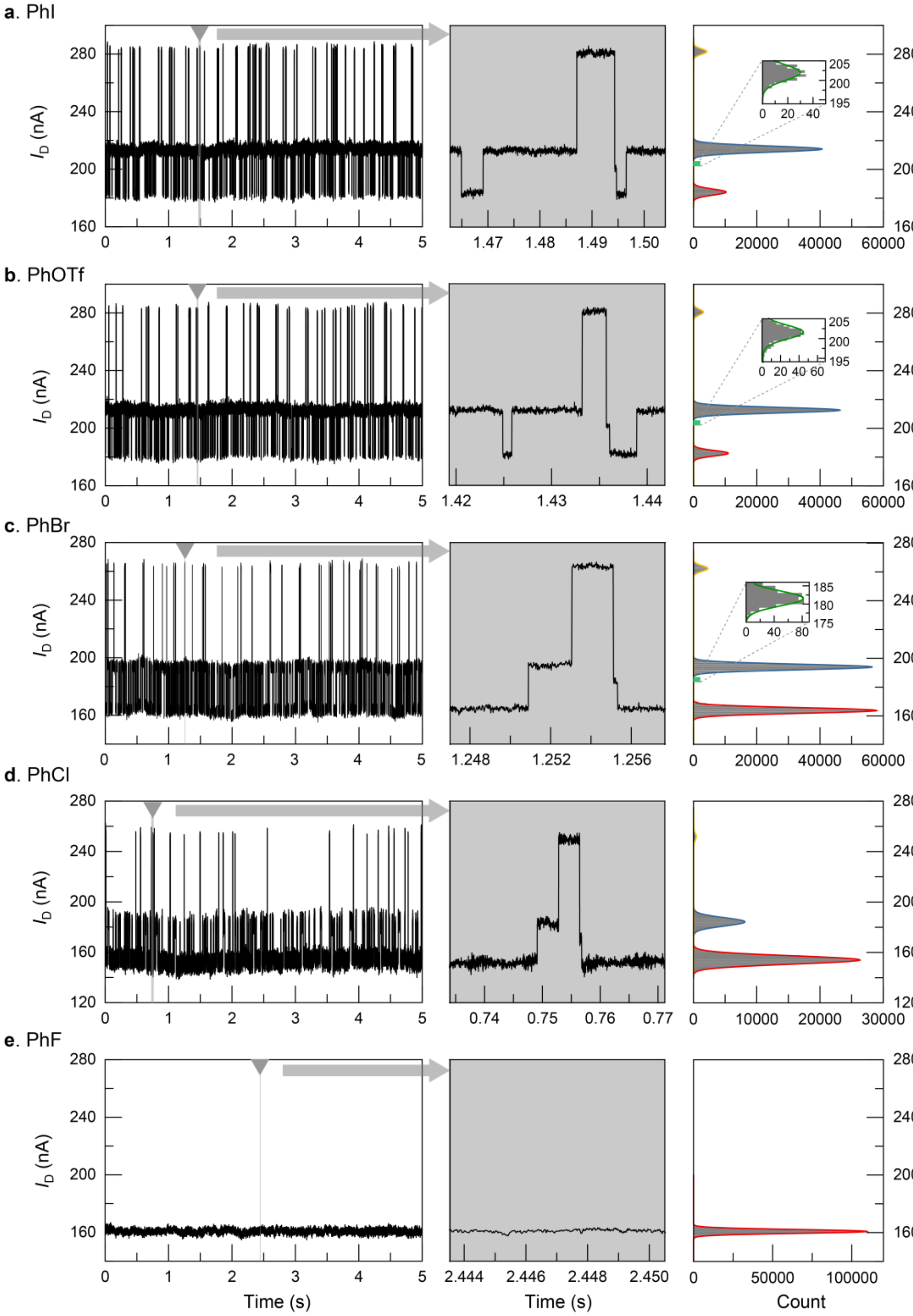

Supplementary Figure 33. Single-molecule Mizoroki-Heck cross-coupling between styrene and different haloarenes at 300 mV and 298 K. The left figures within **a**, **b**, **c**, **d** and **e** are the monitored current signal at the Mizoroki-Heck reaction conditions (the middles are the enlarged figures). The right figures are the corresponding frequency distributions of the current signals.

6. Dynamic Analysis

Other temperature-dependent measurements

Recorded current signals at different temperatures:

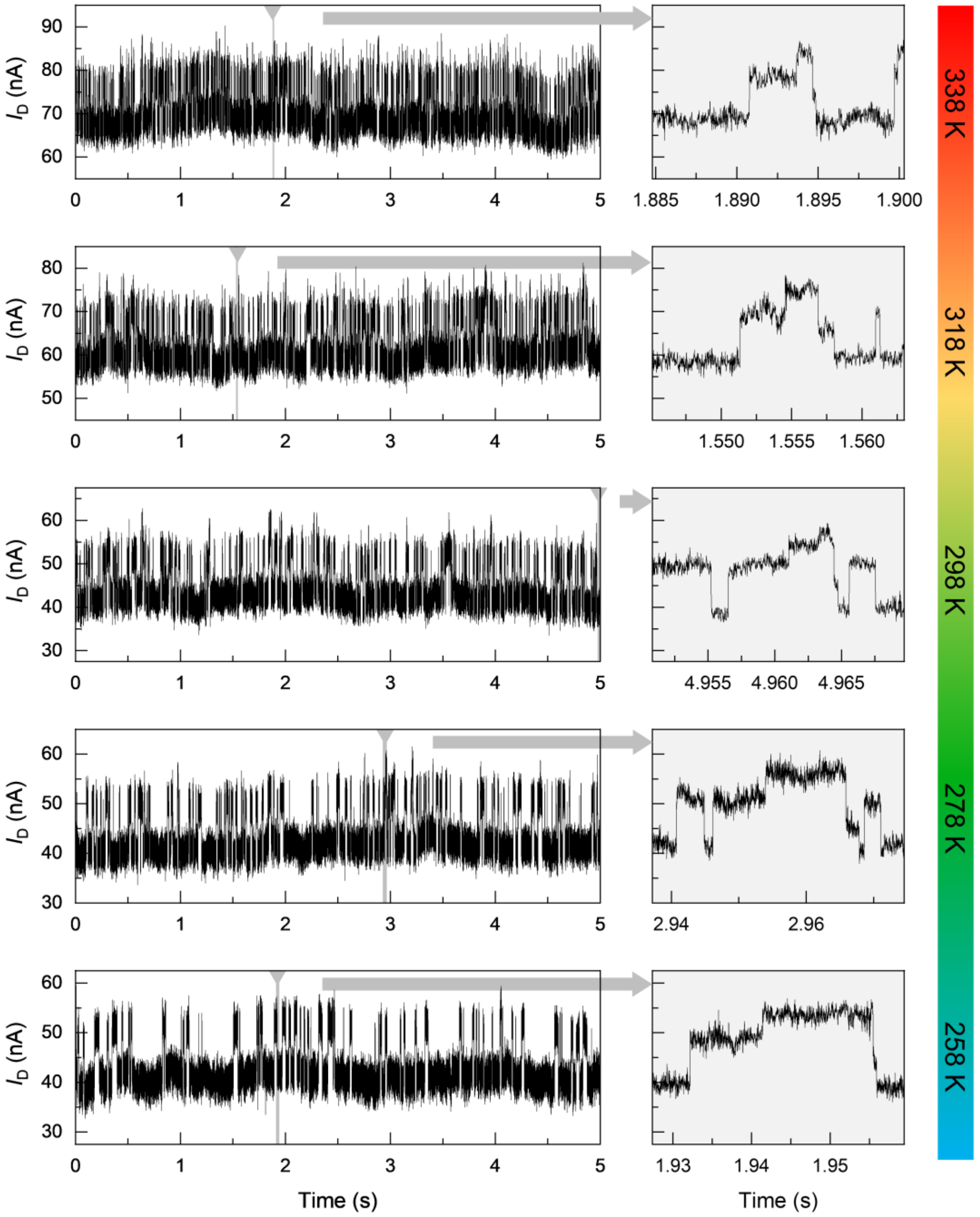

Supplementary Figure 34. Other temperature-dependent measurements at a 300 mV bias voltage. These measurements were conducted by using another single-molecule catalyst device.

Idealisation of the current signal:

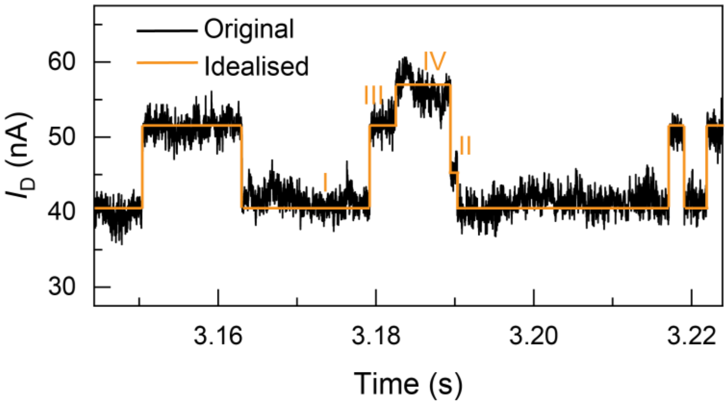

Supplementary Figure 35. *I−t* curves of the single-molecule reaction and their idealised curve fits. The dwell times of the four current levels (I, II, III, IV) and the dwell times before current level transformations can be obtained from the idealised signal.

Kinetic analysis:

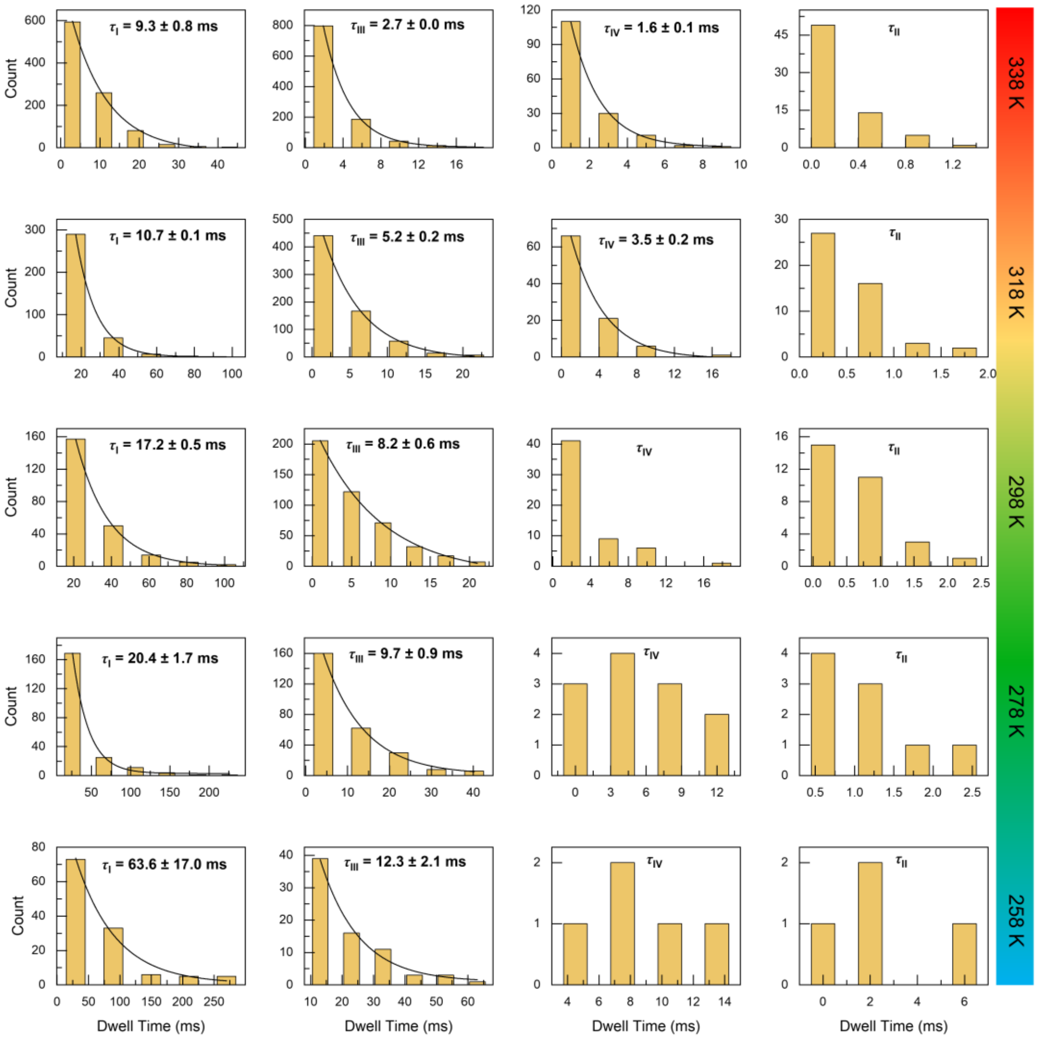

Supplementary Figure 36. Time intervals of the four current levels at various temperatures with single-exponent fits. As the temperature decreased, the dwell time, τ, of each current level became longer, which means that the number of catalytic cycles per unit time decreased. In some cases, the appearance of current levels is insufficient for the single-exponent curve fit.

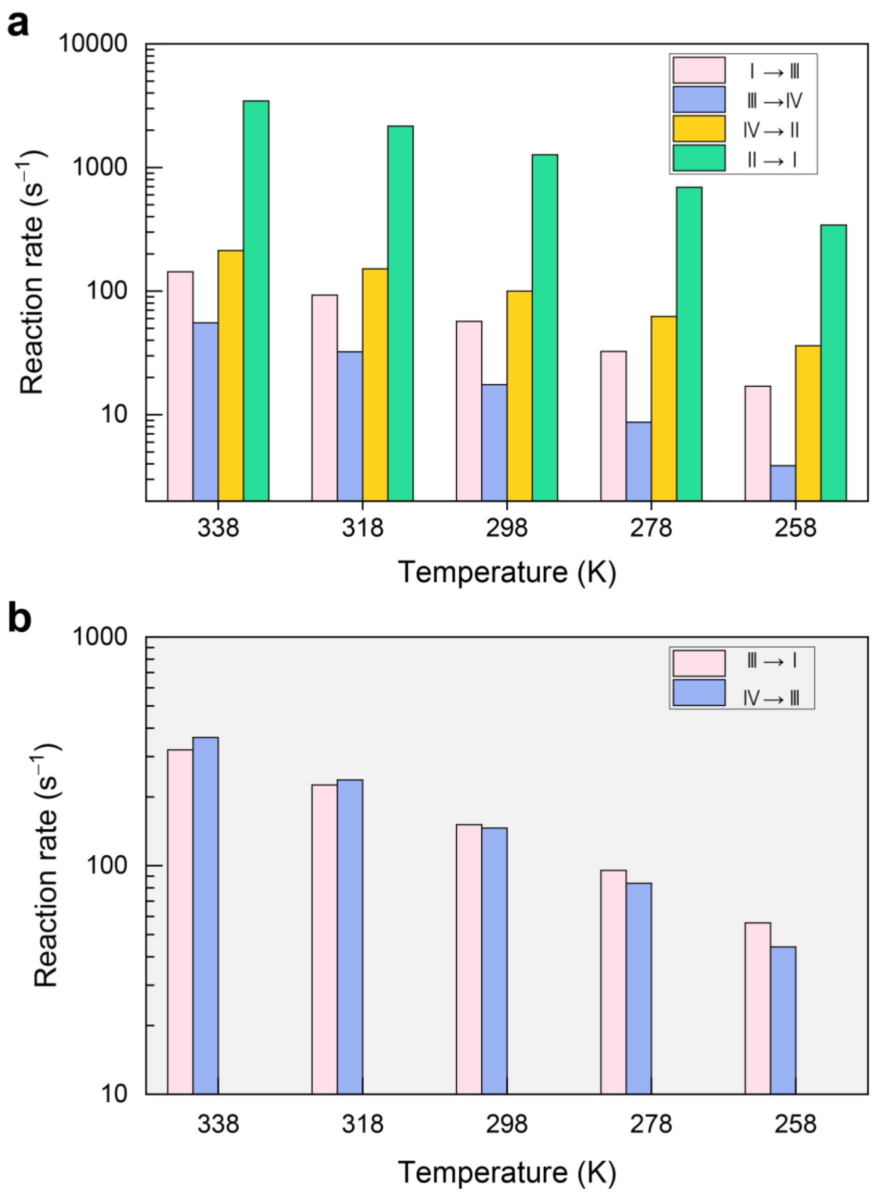

Supplementary Figure 37. Rate constants for each elementary reaction at various temperatures. Rate constants could be obtained via $k = 1/\tau$ (τ was calculated based on the same method in Supplementary Figure 36 with considering reaction trajectory). The single elementary reaction is approximated as a zero-order reaction at 1 mM according to our previous work[10].

Detailed analysis of temperature-dependent measurements in the manuscript

Dwell time of different current levels:

T/K	From I →III	From III →I	From III →IV	From IV →III	From IV →II	From II →IV	From II →I	From I →II
298	6.1	4.6	6.0	19.1	2.9	/	0.5	/
288	11.2	9.4	7.4	3.3	4.4	/	0.5	/
278	13.5	30.0	9.3	10.9	7.7	/	0.8	/
268	16.2	57.2	11.9	19.1	20.5	/	1.4	/
258	29.0	91.2	21.5	44.8	24.2	/	3.1	/

Supplementary Table 1. Dwell time of different current levels in Fig. 3b. Dwell time τ was calculated based on the same method in Supplementary Figure 36. Unit: ms.

Reaction rates of different current levels:

T/K	From I →III	From III →I	From III →IV	From IV →III	From IV →II	From II →IV	From II →I	From I →II
298	163.1	218.3	166.7	52.4	349.6	/	2173.9	/
288	89.7	107.0	136.0	306.7	227.3	/	1851.8	/
278	74.2	33.3	107.8	92.1	130.5	/	1190.5	/
268	61.8	17.5	83.9	52.4	48.8	/	704.2	/
258	34.4	11.0	46.4	22.3	41.4	/	323.6	/

Supplementary Table 2. Reaction rates of different current levels in Fig. 3c. Rate constants could be obtained via $k = 1/\tau$ (τ was calculated based on the same method in Supplementary Figure 36 with considering reaction trajectory). Unit: s^{-1}.

Kinetic and thermodynamic parameters:

T/K	kJ/mol*	From I →III	From III →I	From III →IV	From IV →III	From IV →II	From II →IV	From II →I	From I →II
	E_a	21.1	53.4	20.1	29.2	37.2	/	35.8	/
	ΔH	−32.3		−9.1		/		/	
	$\Delta H^‡$	18.6	50.9	17.6	26.7	34.7	/	33.3	/
	$\Delta G^‡$	60.4	59.6	60.3	63.2	58.5	/	53.9	/
298	ΔG	0.8		−2.9		/		/	
	$\Delta S^‡$	−140.1	−29.2	−143.2	−122.3	−79.7	/	−69.2	/
	ΔS	−110.9		−20.9		/		/	
	$\Delta H^‡$	18.7	51.0	17.7	26.8	34.8	/	33.4	/
	$\Delta G^‡$	59.7	59.3	58.7	56.7	57.5	/	52.4	/
288	ΔG	0.4		2.0		/		/	
	$\Delta S^‡$	−142.3	−28.7	−142.3	−103.9	−78.7	/	−66.1	/
	ΔS	−113.6		−38.4		/		/	
	$\Delta H^‡$	18.8	51.1	17.8	26.9	34.9	/	33.5	/
	$\Delta G^‡$	58.0	59.8	57.1	57.5	56.7	/	51.6	/
278	ΔG	−1.8		−0.4		/		/	
	$\Delta S^‡$	−140.9	−31.4	−141.4	−110.0	−78.3	/	−65.0	/
	ΔS	−109.5		−31.4		/		/	
	$\Delta H^‡$	18.9	51.2	17.9	27.0	35.0	/	33.6	/
	$\Delta G^‡$	56.2	59.0	55.5	56.6	56.7	/	50.8	/
268	ΔG	−2.8		−1.1		/		/	
	$\Delta S^‡$	−139.3	−29.3	−140.5	−110.5	−81.2	/	−64.2	/
	ΔS	−110.0		−30.0		/		/	
	$\Delta H^‡$	19.0	51.3	18.0	27.1	35.1	/	33.7	/
	$\Delta G^‡$	55.3	57.7	54.6	56.2	54.9	/	50.5	/
258	ΔG	−2.4		−1.6		/		/	
	$\Delta S^‡$	−140.8	−25.1	−142.2	−113.0	−76.9	/	−65.2	/
	ΔS	−115.7		−29.2		/		/	

Supplementary Table 3. Kinetic and thermodynamic parameters of single-molecule Mizoroki-Heck cross-coupling at different temperatures calculated from the *I–t* curves in Fig. 3b. For the thermodynamic constants of every elementary reaction, we can acquire $\Delta G^‡$ at different temperatures through the Eyring equation ($k = (k_BT/h) \times \exp(-\Delta G^‡/RT)$). The activation energy of each process is provided in Fig. 3d through the linear fitting of ln*k* versus 1000/T according to the Arrhenius equation ($k = A\times\exp(-E_a/RT)$). Then, $\Delta H^‡$ can be obtained by $\Delta H^‡ = E_a - RT$ and $\Delta S^‡ = (\Delta H^‡ - \Delta G^‡)/T$. Finally, ΔG, ΔH and

ΔS can be acquired through the difference between $\Delta G^{\ddagger}$, $\Delta H^{\ddagger}$ and $\Delta S^{\ddagger}$ of the forward and backward reactions, respectively. For the overall reaction, the thermodynamic constants are the total of each elementary reaction according to the Hess's law. *The units of $\Delta S^{\ddagger}$ and ΔS are J/(mol·K).

7. Gate Tuning of the Single-molecule Mizoroki-Heck Reaction

Gate-voltage-dependent measurement:

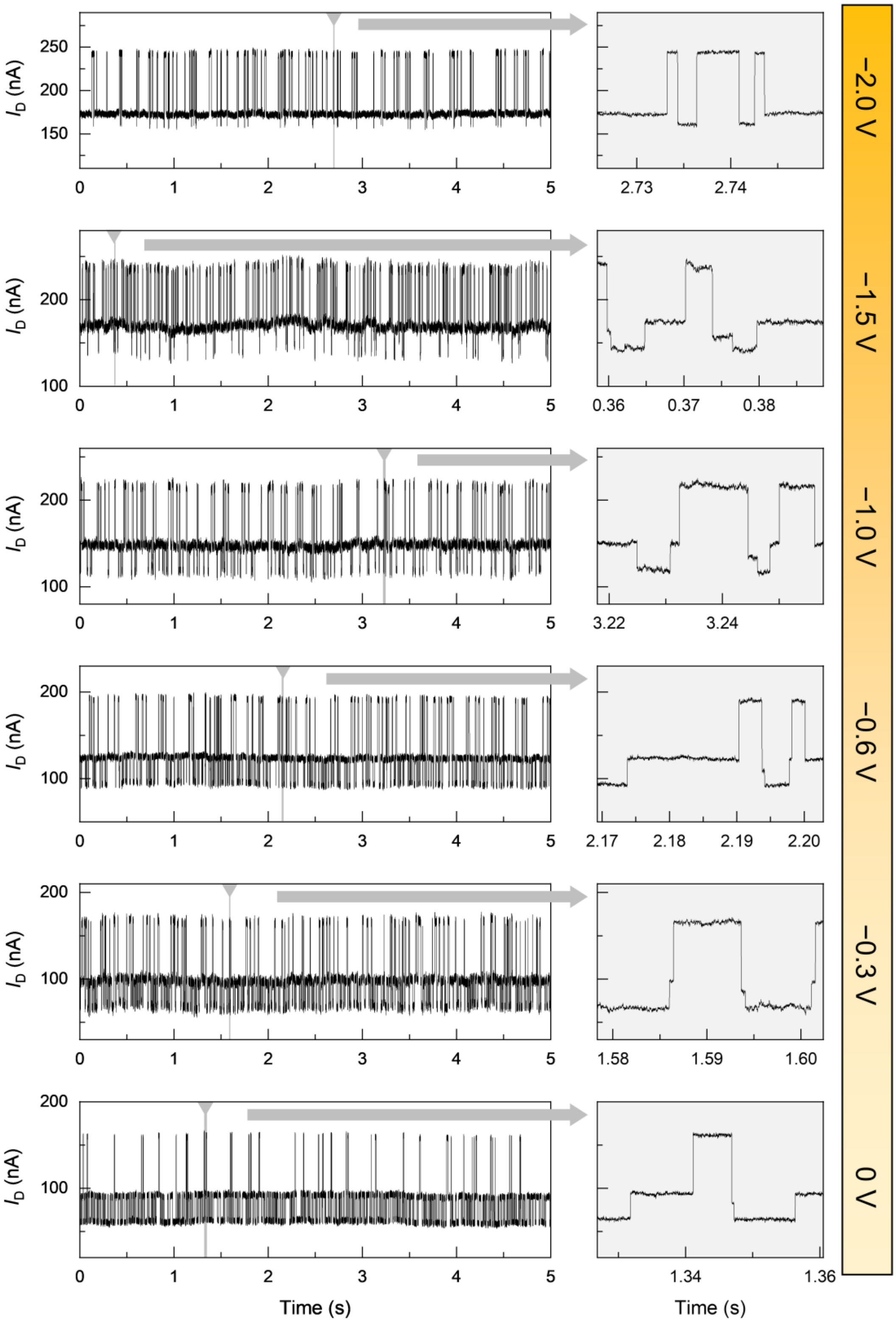

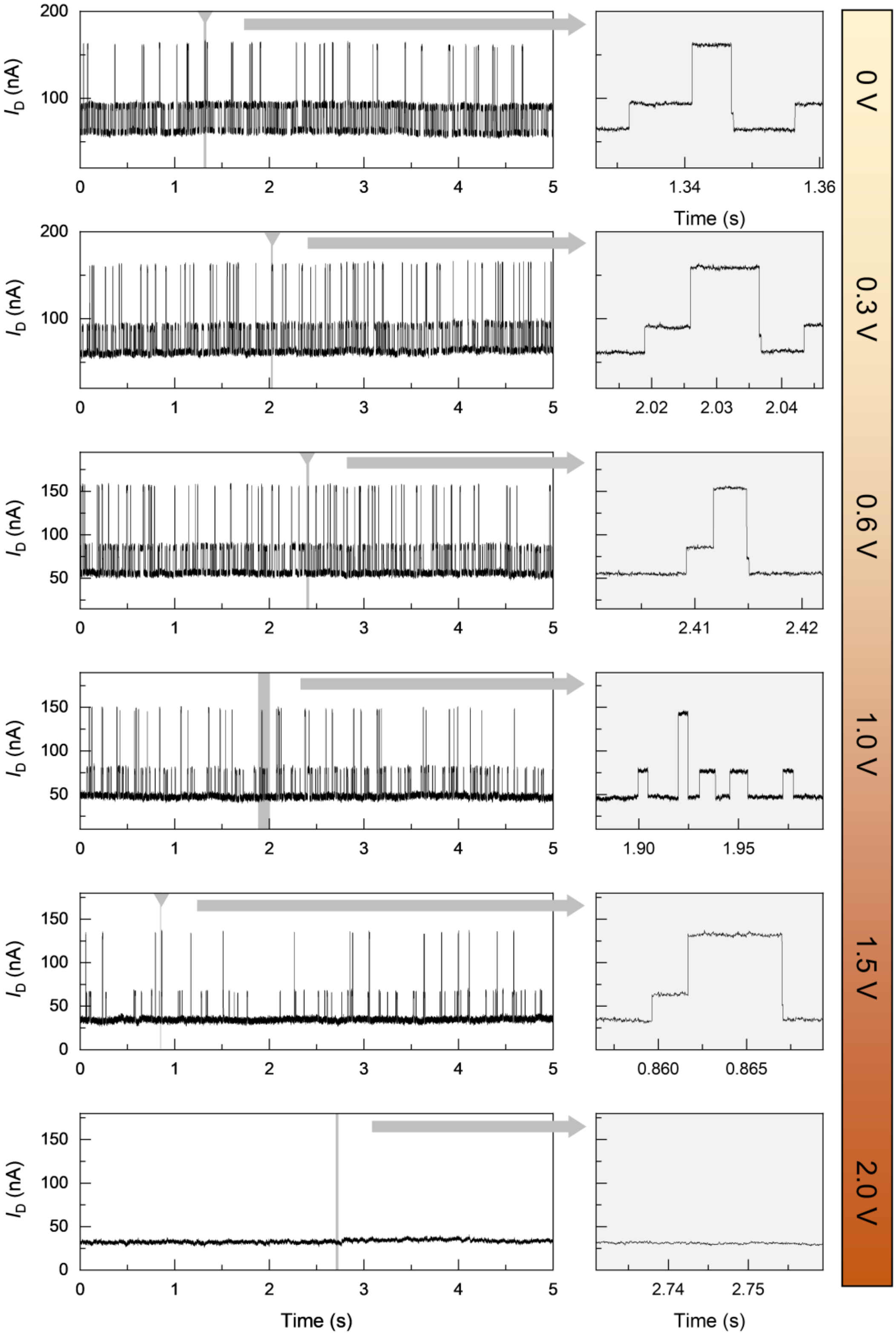

Supplementary Figure 38. Measurements at a 300 mV bias voltage and different gate voltages.

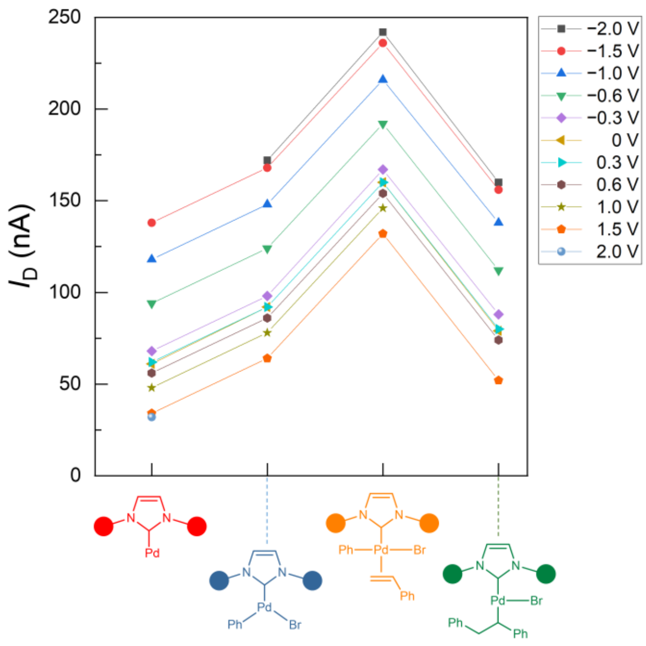

Supplementary Figure 39. Current level values under different gate voltages (bias voltage: 300 mV).

Kinetic analysis:

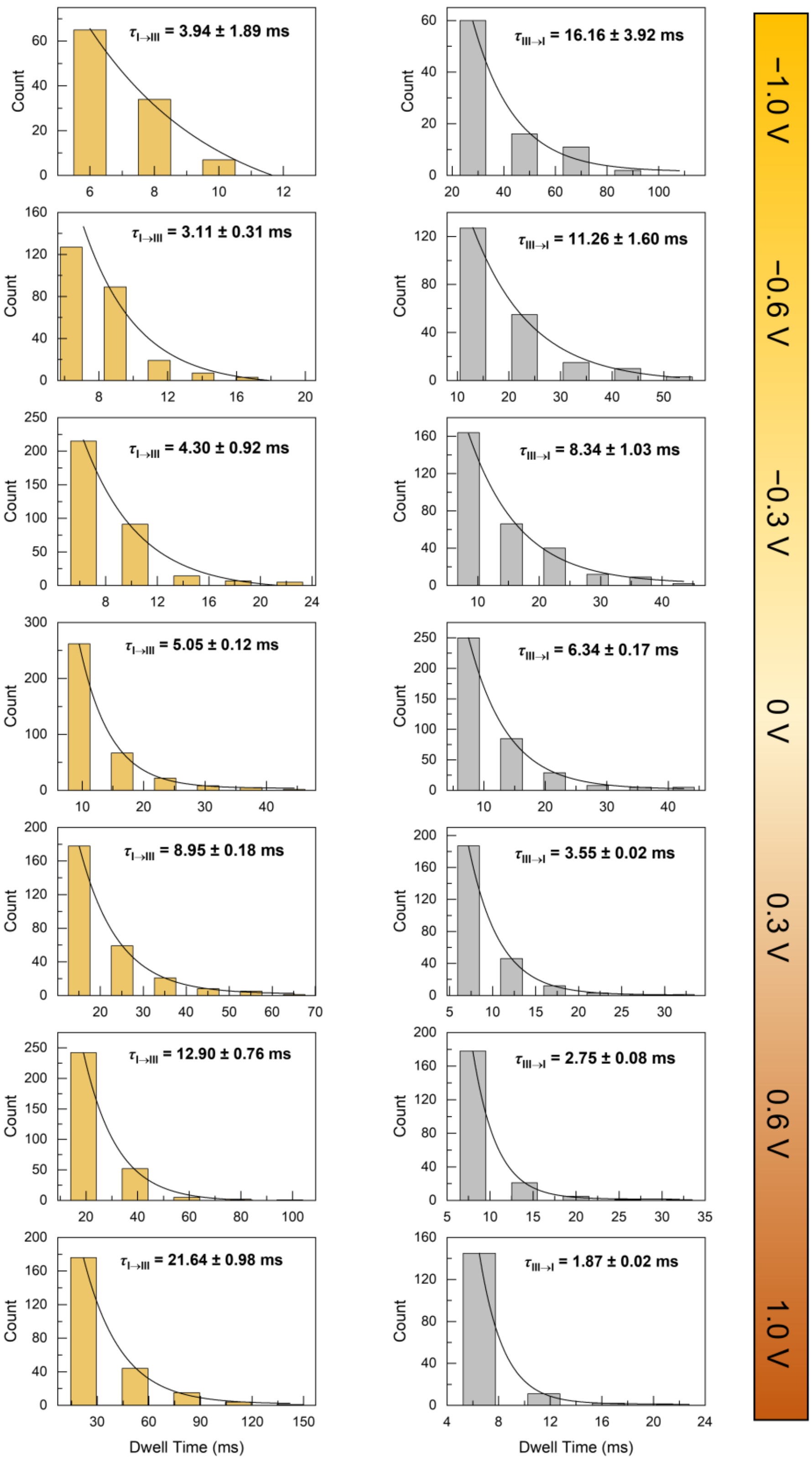

Supplementary Figure 40. Time intervals of oxidative addition of PhBr to Pd(0) and its reverse process at various gate voltages with single-exponent fits. Rate constants could be obtained via $k = 1/\tau$.

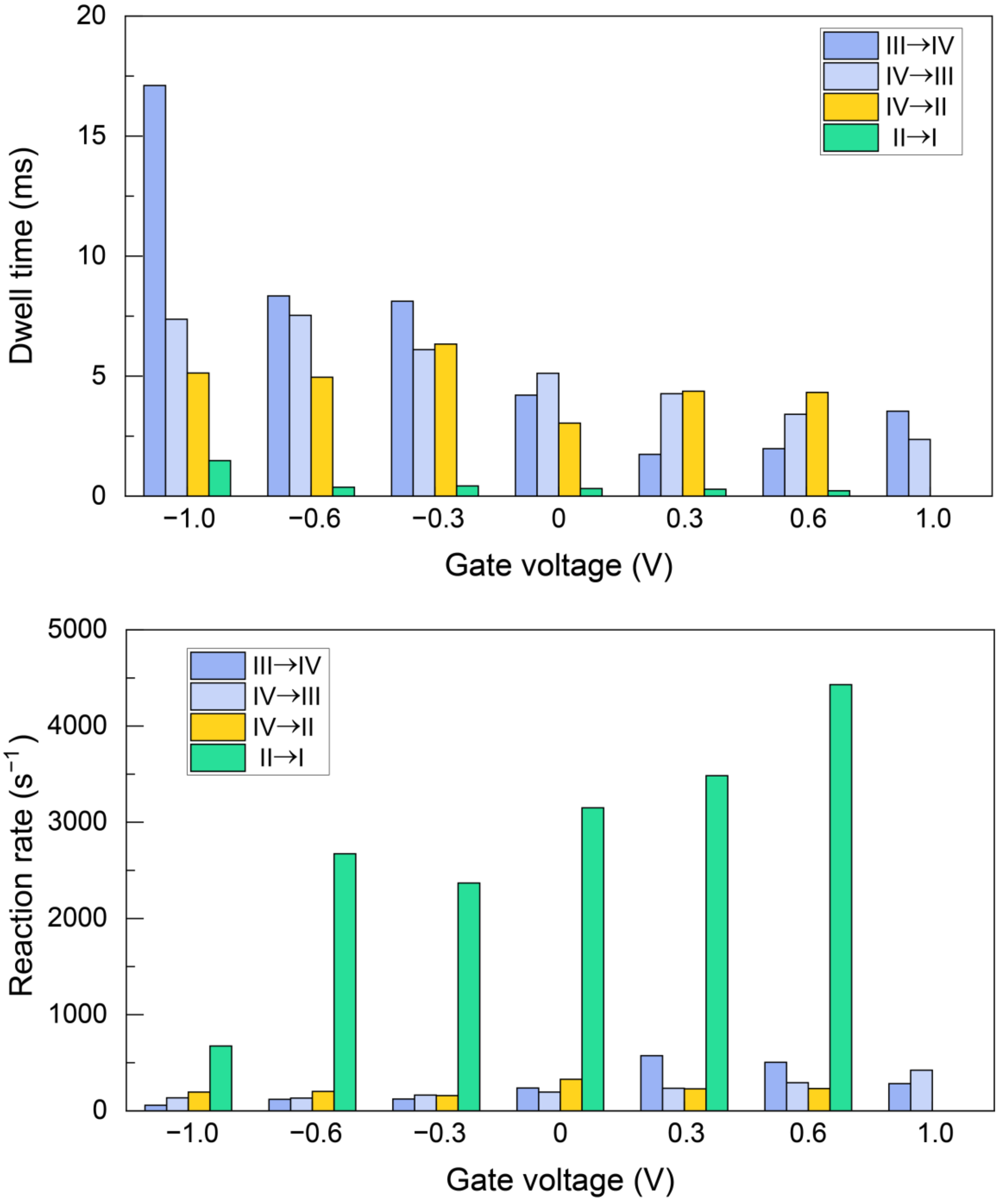

Supplementary Figure 41. The tuning effect on olefin coordination (forward and reverse processes), olefin insertion, *β*-H elimination and reductive elimination via gating. For the process III→IV, the dwell time of the current level III is recorded and analysed (the same rule for other processes). The dwell time is shown as the mean value.

Other gate-voltage-dependent measurements:

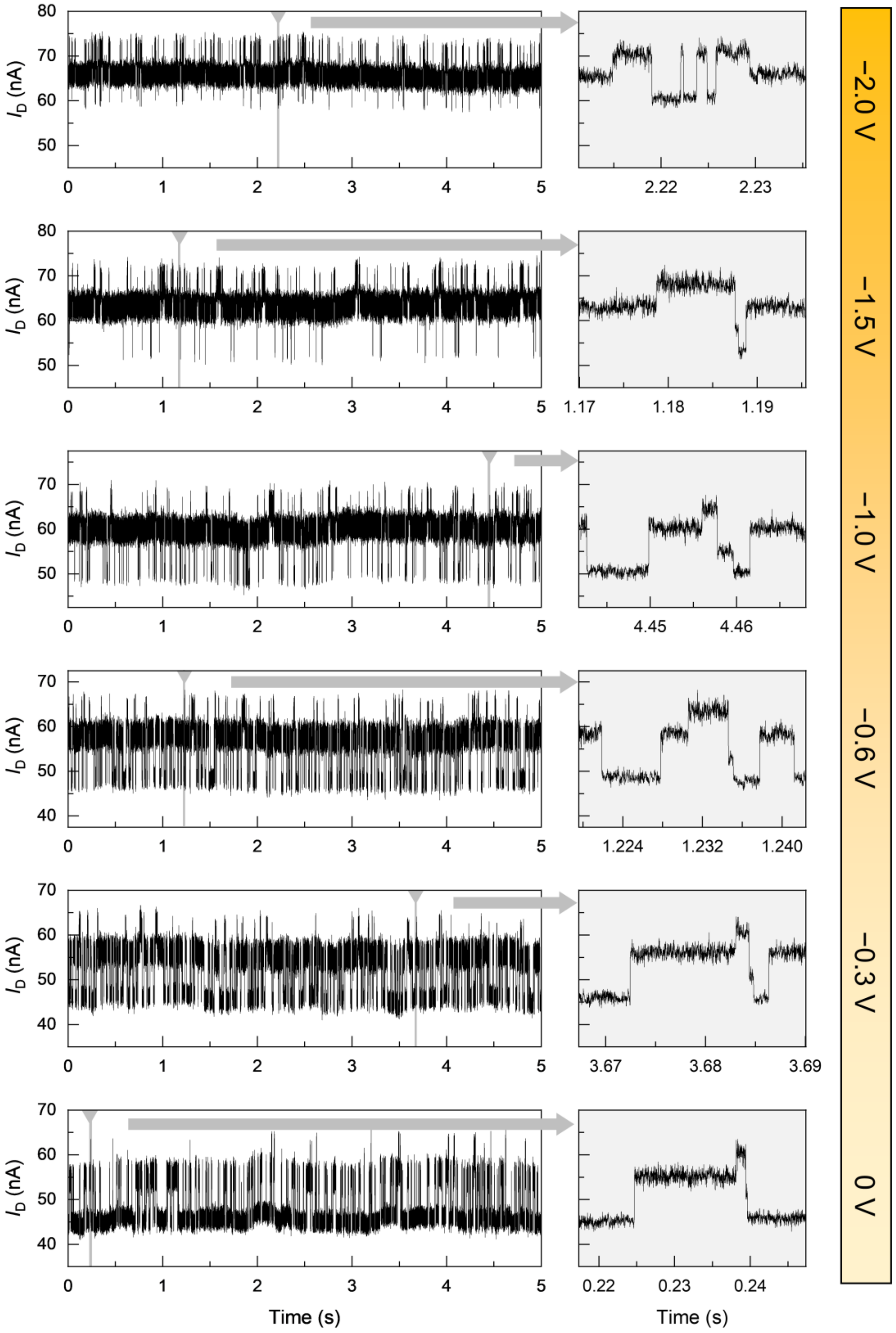

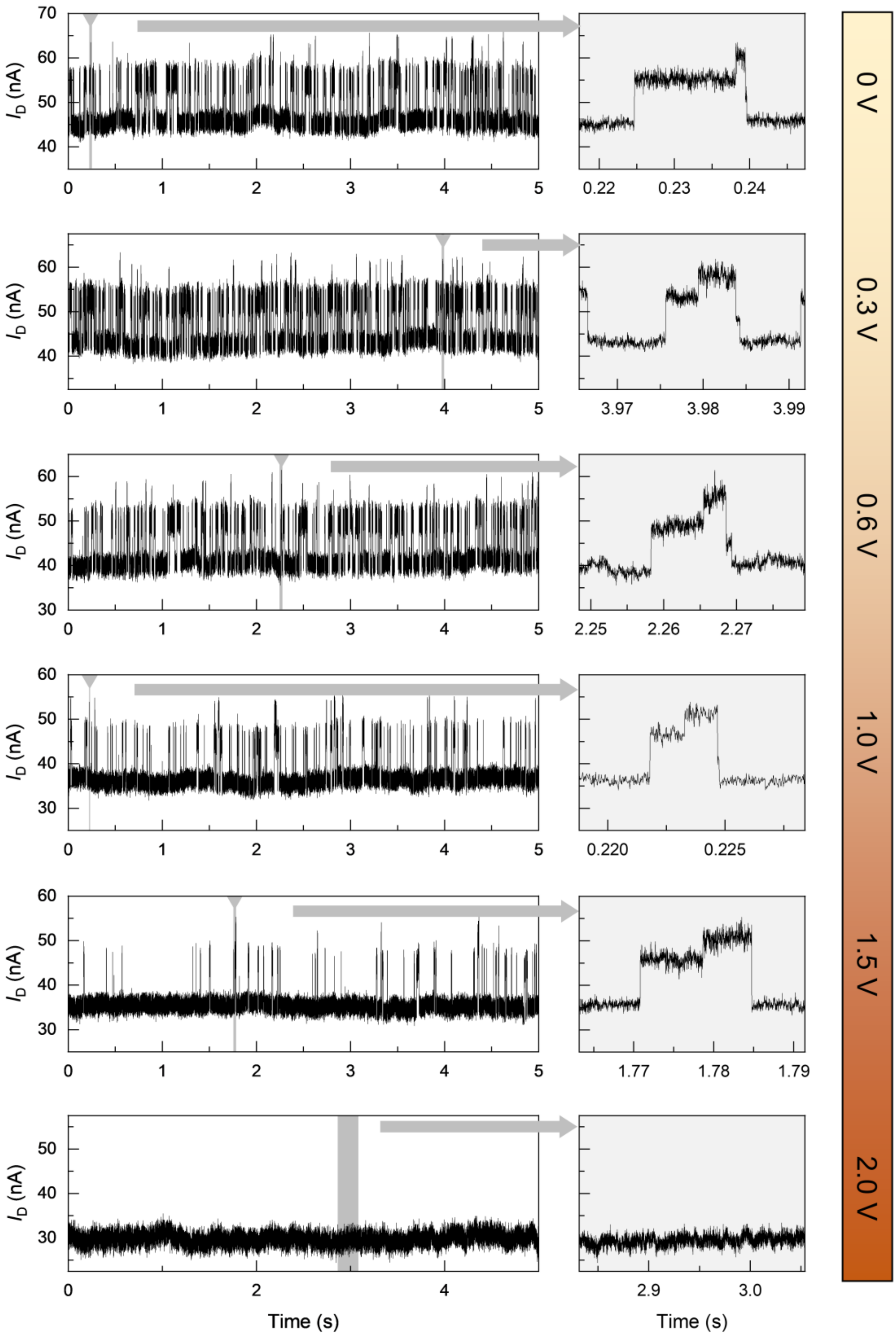

Supplementary Figure 42. Other gate-voltage- dependent measurements at 300 mV bias voltage. These measurements were conducted by using another single-molecule catalyst device.

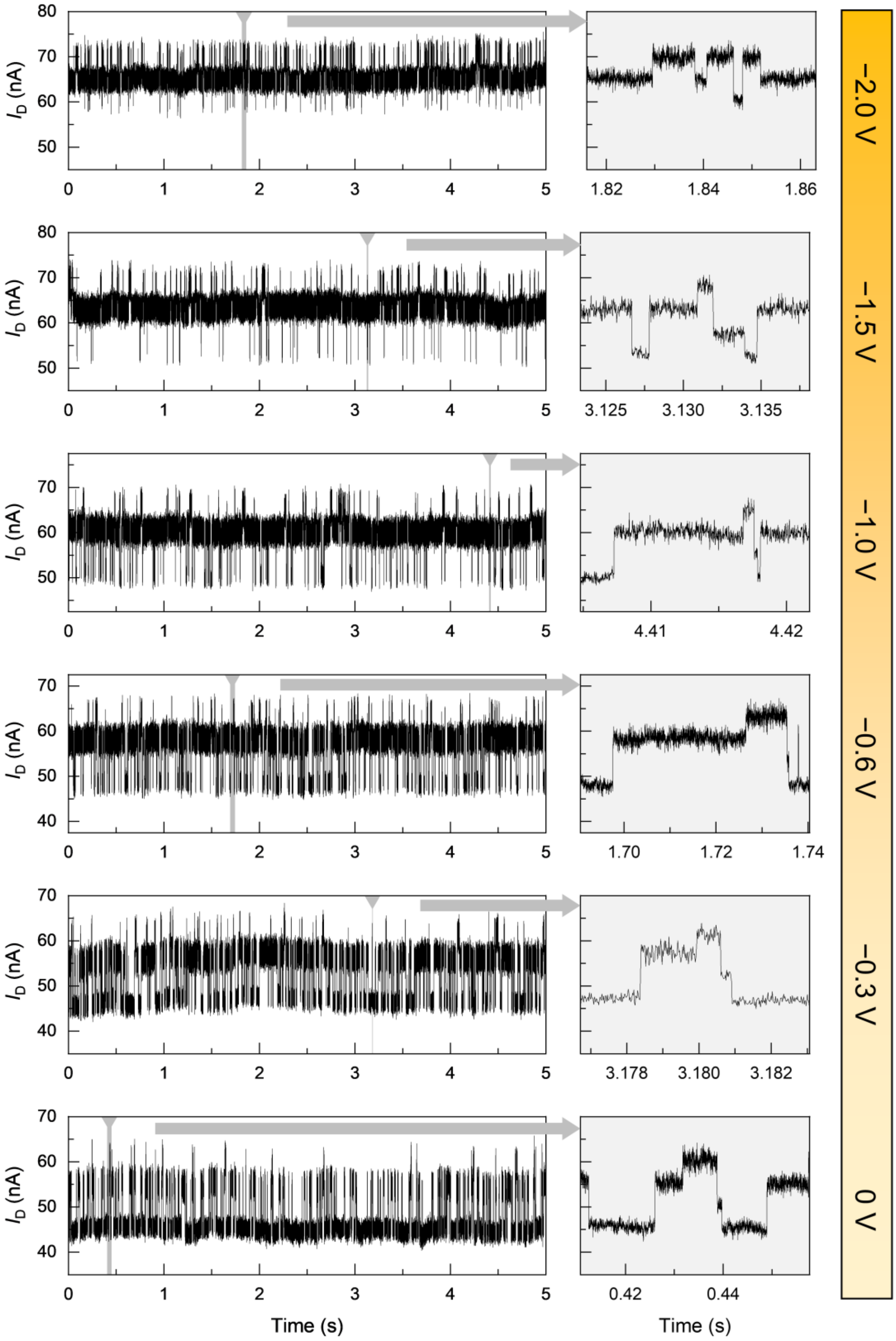

I_D (nA)
Time (s)
−2.0 V
−1.5 V
−1.0 V
−0.6 V
−0.3 V
0 V

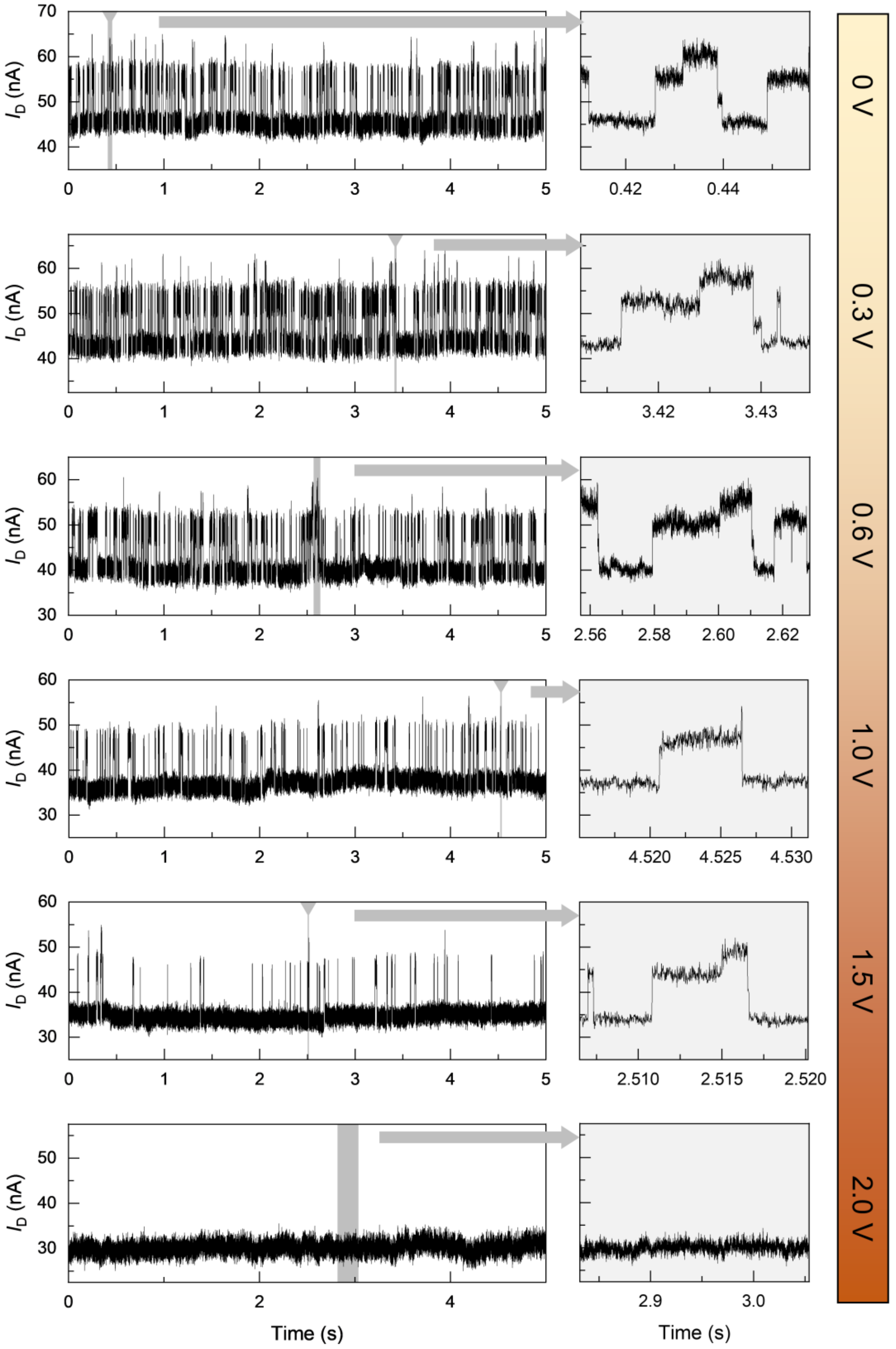

Supplementary Figure 43. Other gate-voltage- dependent measurements at 300 mV bias voltage.

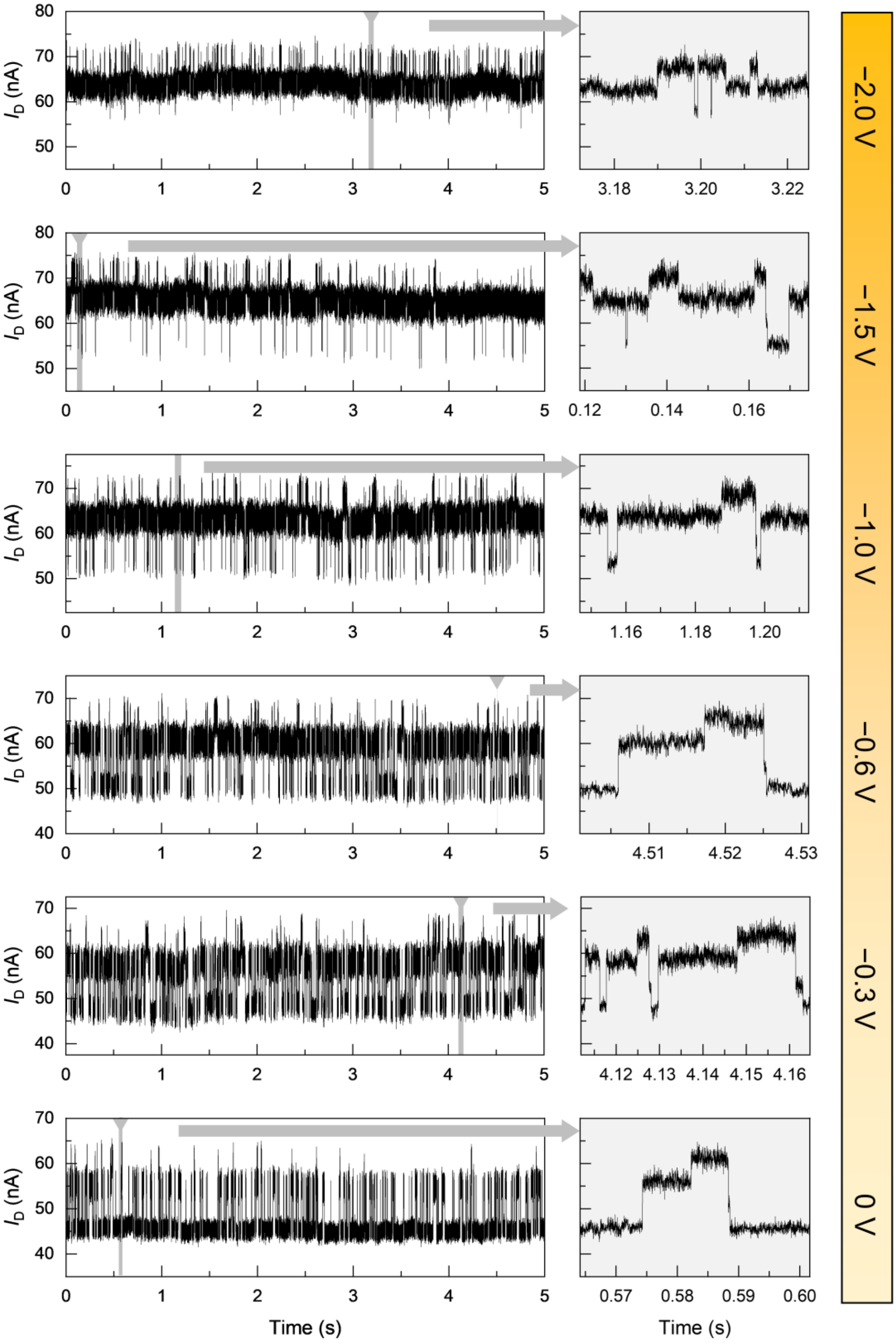

I_D (nA)
Time (s)
−2.0 V
−1.5 V
−1.0 V
−0.6 V
−0.3 V
0 V
3.18
3.20
3.22
0.12
0.14
0.16
1.16
1.18
1.20
4.51
4.52
4.53
4.12
4.13
4.14
4.15
4.16
0.57
0.58
0.59
0.60

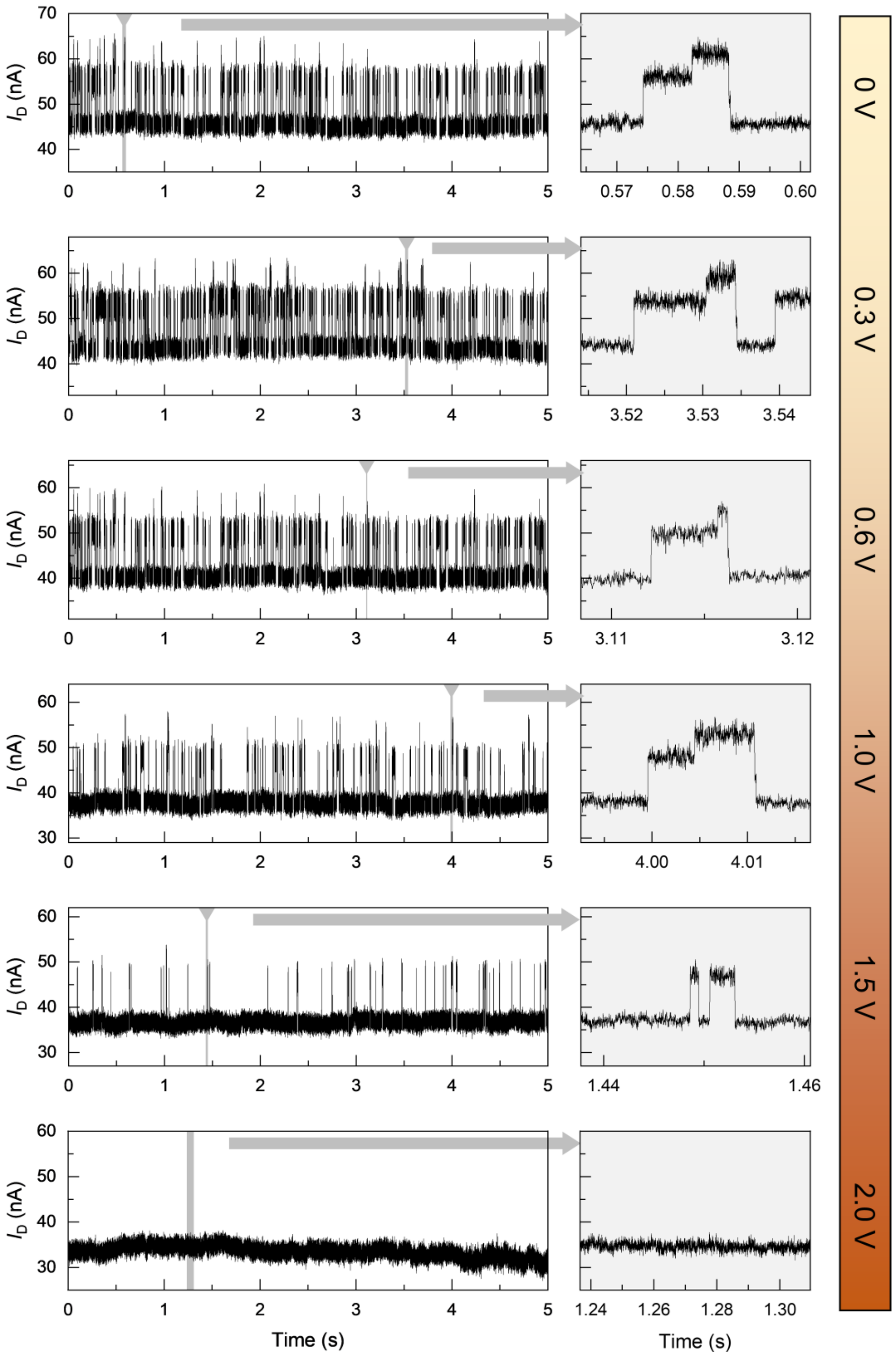

Supplementary Figure 44. Other gate-voltage- dependent measurements at 300 mV bias voltage.

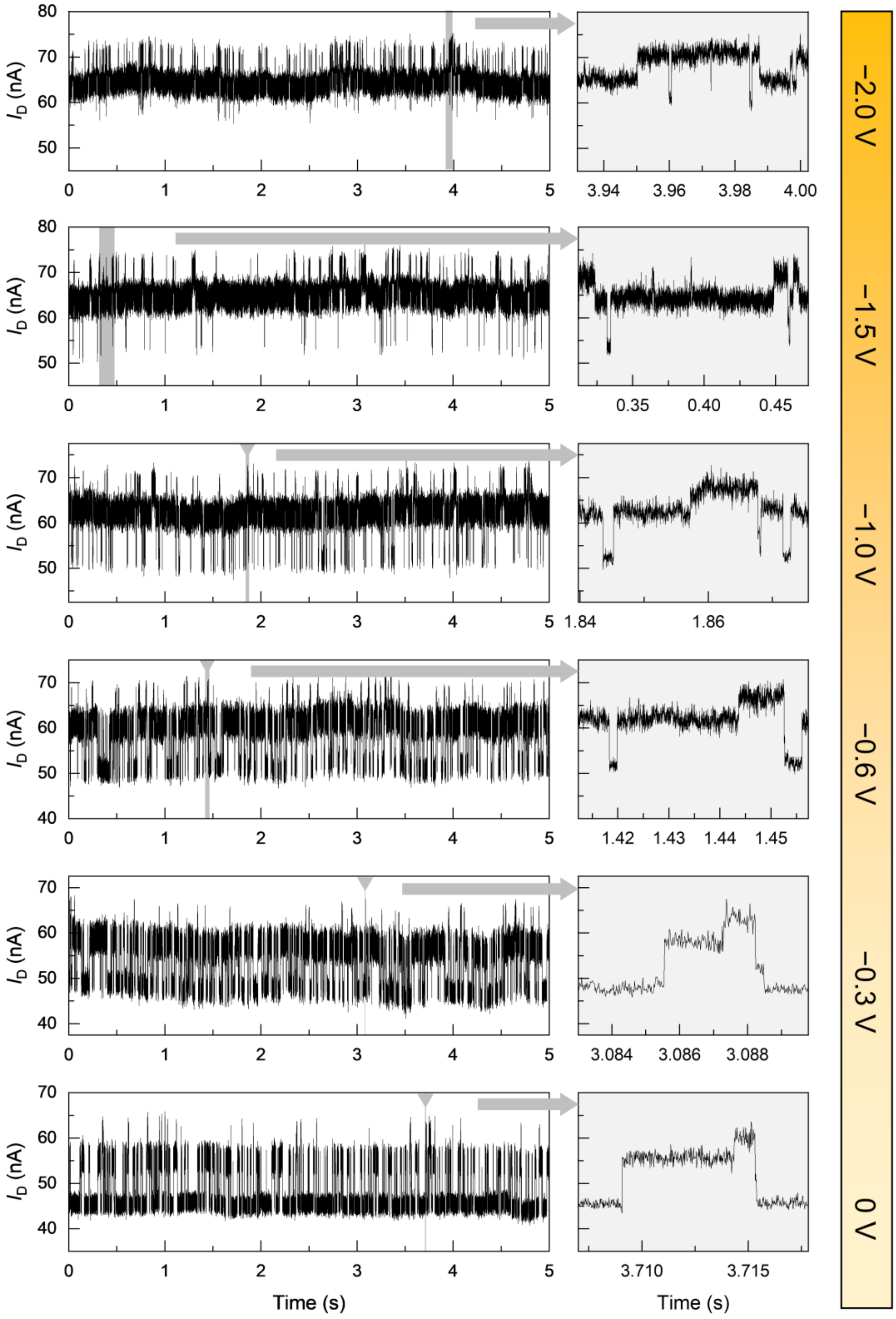

I_D (nA)
Time (s)
−2.0 V
−1.5 V
−1.0 V
−0.6 V
−0.3 V
0 V
3.94 3.96 3.98 4.00
0.35 0.40 0.45
1.84 1.86
1.42 1.43 1.44 1.45
3.084 3.086 3.088
3.710 3.715

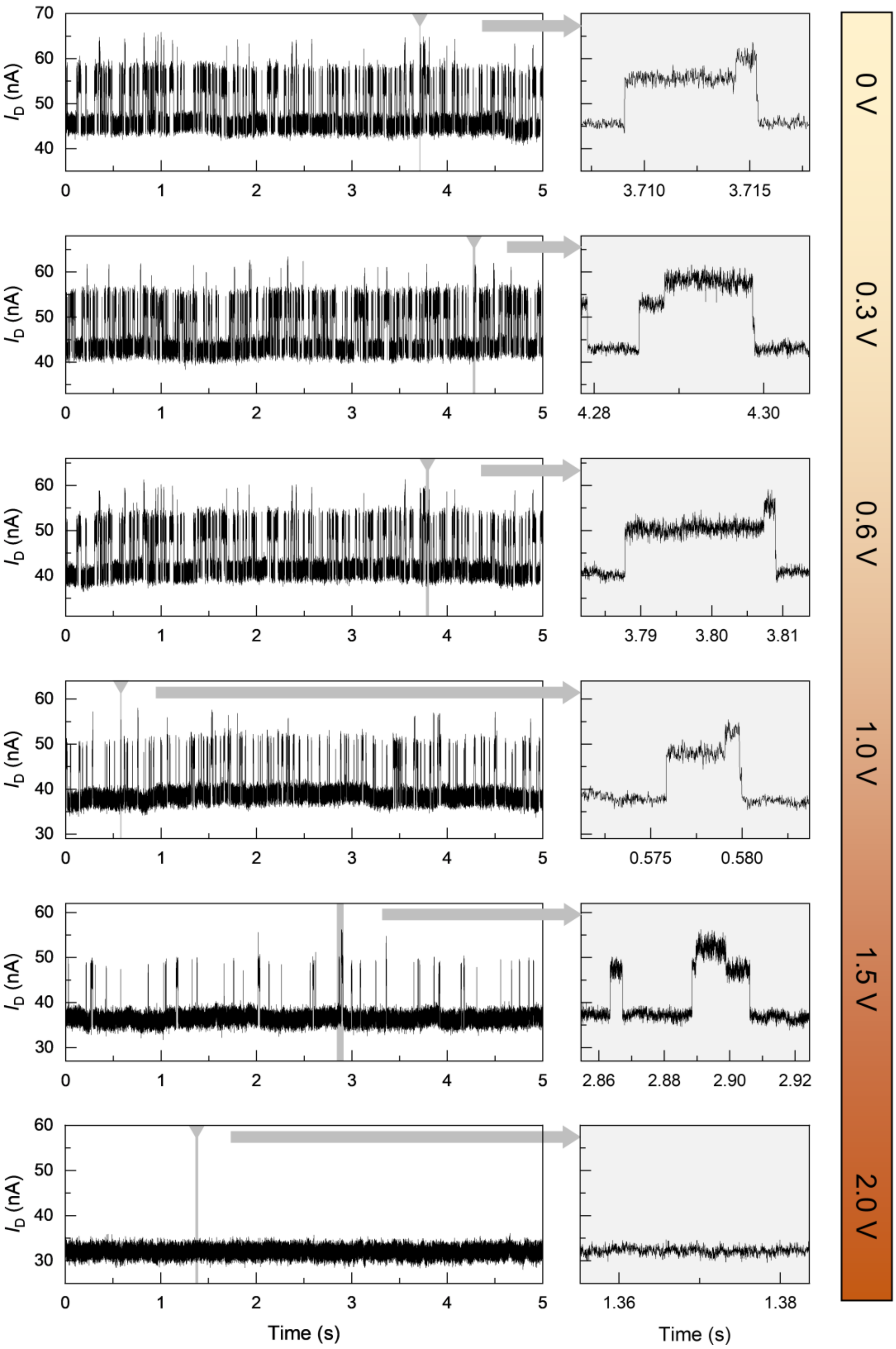

Supplementary Figure 45. Other gate-voltage- dependent measurements at 300 mV bias voltage.

8. Supplementary Movie

Supplementary Movie. Fluorescent and current signals at the single-molecule catalyst site during the Mizoroki-Heck cross-coupling. The real-time monitoring of the blinking catalyst site shows that the fluorescent and current signals are highly correlated. The electrical current signal (blue line) and the optical fluorescent intensity (flesh pink line) was displayed via 3× slowdown.

9. Atomic Coordinates

In DMF:

Pd(0)

N	1.07921400	0.00003300	0.85850200
C	2.46182900	0.00004500	0.44488100
N	-1.07921500	-0.00006900	0.85850200
C	3.11778100	-1.23569900	0.26161300
C	4.46011000	-1.20685400	-0.14049200
H	4.98964200	-2.14317800	-0.29323600
C	5.12537600	0.00005700	-0.34536200
H	6.16648200	0.00007000	-0.65802600
C	4.45984600	1.20697100	-0.14132600
H	4.98924600	2.14330000	-0.29453700
C	3.11750500	1.23580900	0.26069400
C	2.42996200	-2.57828200	0.49142400
H	1.39850200	-2.38424700	0.79820900
C	2.37526200	-3.41560000	-0.79984600
H	1.86308500	-2.87052100	-1.60217800
H	1.82857100	-4.35007800	-0.62251100
H	3.37939100	-3.67856900	-1.15396700
C	3.10605100	-3.36602400	1.62966300
H	4.14513000	-3.61685800	1.38450100
H	2.57047200	-4.30591100	1.81143200
H	3.10987600	-2.79366400	2.56503000
C	2.42961600	2.57838400	0.49021600
H	1.39800400	2.38443900	0.79653700
C	2.37549200	3.41597200	-0.80087800
H	3.37972200	3.67878400	-1.15479900
H	1.82901800	4.35055800	-0.62345100
H	1.86325900	2.87119900	-1.60338500
C	3.10547200	3.36583100	1.62881600
H	3.10837600	2.79364400	2.56428000
H	2.57040500	4.30609900	1.81014500
H	4.14487600	3.61586700	1.38416900
C	0.00000000	-0.00006600	0.00460700
C	0.67725300	0.00007800	2.19259600
H	1.38904900	0.00018800	3.00391400
C	-0.67725400	-0.00008400	2.19259600
H	-1.38904900	-0.00014800	3.00391400
C	-2.46182900	-0.00002300	0.44488000
C	-3.11758400	-1.23575700	0.26076700
C	-4.45992400	-1.20685600	-0.14125500

H	-4.98938400	-2.14316000	-0.29440900
C	-5.12537600	0.00008800	-0.34536400
H	-6.16648200	0.00012400	-0.65802900
C	-4.46003300	1.20697000	-0.14056600
H	-4.98950400	2.14331800	-0.29336700
C	-3.11770300	1.23575200	0.26153800
C	-2.42978500	-2.57836300	0.49037500
H	-1.39816800	-2.38446900	0.79671100
C	-3.10571800	-3.36570900	1.62900100
H	-3.10861100	-2.79347300	2.56443400
H	-2.57071100	-4.30599800	1.81039500
H	-4.14513000	-3.61569700	1.38434100
C	-2.37568700	-3.41602000	-0.80067400
H	-3.37992600	-3.67877300	-1.15461200
H	-1.82929000	-4.35064000	-0.62318500
H	-1.86338700	-2.87132600	-1.60319200
C	-2.42979300	2.57830300	0.49126400
H	-1.39833800	2.38421700	0.79803500
C	-2.37506700	3.41555500	-0.80004700
H	-1.86295700	2.87040000	-1.60237000
H	-1.82830000	4.35000000	-0.62277200
H	-3.37918700	3.67858400	-1.15415100
C	-3.10580600	3.36614400	1.62948000
H	-4.14487600	3.61702600	1.38433000
H	-2.57016600	4.30600800	1.81118700
H	-3.10964200	2.79383000	2.56487500
Pd	0.00000100	-0.00009600	-1.96380200

INT1

Pd	0.12772400	0.96166100	-0.41489800
N	-1.35570300	-1.65073000	0.48450600
C	-2.67155100	-1.13247300	0.19356600
N	0.77230200	-1.86742000	0.69943000
C	-3.22163800	-1.35235600	-1.08723000
C	-4.49927000	-0.83547700	-1.34256800
H	-4.94761300	-0.98505600	-2.32068700
C	-5.20612300	-0.14281400	-0.36191400
H	-6.19596400	0.24921400	-0.58137000
C	-4.65229300	0.03404400	0.90414000
H	-5.21888000	0.55900200	1.66808900
C	-3.37774000	-0.46041900	1.21361700
C	-2.50104700	-2.14838500	-2.17249600

H	-1.51815700	-2.43706000	-1.78928000
C	-2.26779600	-1.31216800	-3.44387800
H	-1.68164100	-0.41226900	-3.22453200
H	-1.71630700	-1.90164600	-4.18673700
H	-3.21267400	-0.99874000	-3.90375400
C	-3.26352200	-3.44681000	-2.50119800
H	-4.25711400	-3.23588900	-2.91491500
H	-2.71077400	-4.03483400	-3.24413800
H	-3.39644900	-4.07028600	-1.60913800
C	-2.82531300	-0.28586400	2.62563100
H	-1.81301800	-0.69851100	2.65065700
C	-2.72580600	1.19670600	3.02810600
H	-3.71166000	1.67556900	3.06274600
H	-2.27745000	1.28765300	4.02519700
H	-2.10060000	1.75817800	2.32426600
C	-3.66668800	-1.07644800	3.64671500
H	-3.70217600	-2.14322200	3.39582800
H	-3.23553800	-0.98010400	4.65079600
H	-4.69821300	-0.70631700	3.68881300
C	-0.18051900	-0.97142400	0.28434800
C	-1.13593400	-2.92399900	1.00223600
H	-1.94660500	-3.60222400	1.21963500
C	0.20573600	-3.06022600	1.13851300
H	0.80503500	-3.88118800	1.50123100
C	2.19526200	-1.62498100	0.67906600
C	2.92961800	-1.99087700	-0.46913800
C	4.30893900	-1.74182000	-0.46438300
H	4.90079300	-2.00883200	-1.33534000
C	4.93327700	-1.16433700	0.63926500
H	6.00425800	-0.97878400	0.62178000
C	4.18901100	-0.83685000	1.77055600
H	4.68784700	-0.40285600	2.63259700
C	2.80661600	-1.06417600	1.82014100
C	2.28972800	-2.66109900	-1.68195200
H	1.21507900	-2.74625200	-1.49800300
C	2.83406500	-4.08965000	-1.87579500
H	2.67274000	-4.70448700	-0.98246700
H	2.32671300	-4.57830900	-2.71673900
H	3.90938000	-4.08527100	-2.09136600
C	2.47014700	-1.82699800	-2.96365100
H	3.52785200	-1.72163700	-3.23333700
H	1.95918900	-2.31255900	-3.80428200
H	2.04848100	-0.82198000	-2.84756000
C	2.03435900	-0.73264900	3.09379400

H	0.97712900	-0.95139200	2.91949400
C	2.13896700	0.75971700	3.45704700
H	1.77908200	1.39223600	2.63715100
H	1.53034700	0.97649000	4.34361400
H	3.17167900	1.05117900	3.68309400
C	2.49839600	-1.61762900	4.26693600
H	3.55052800	-1.43388400	4.51566200
H	1.90085300	-1.40582300	5.16211000
H	2.38893700	-2.68323300	4.03283900
C	0.61566600	2.97405100	-0.97567600
C	2.01221500	2.91588500	-1.21998800
C	-0.31444600	2.68227700	-2.00472700
C	2.46206900	2.59330500	-2.49721600
H	2.71427900	3.16318200	-0.43071200
C	0.18276400	2.35714600	-3.28899500
C	1.55056200	2.33330300	-3.53679200
H	3.53132800	2.55677800	-2.68790400
H	-0.52629500	2.16693200	-4.09030600
Br	0.00125700	4.09763200	0.59140200
H	-1.37416500	2.86832000	-1.86586300
H	1.91799100	2.11241600	-4.53495800

TS1

Pd	-0.09155400	1.05277800	-0.22576000
N	1.23327400	-1.72688700	0.45210800
C	2.56676500	-1.17642600	0.49071600
N	-0.89724700	-1.94079700	0.27491300
C	3.04745400	-0.64773600	1.70790200
C	4.34614300	-0.12049300	1.71869300
H	4.74581900	0.29223700	2.64060900
C	5.13573700	-0.12580900	0.57073200
H	6.14025500	0.28849600	0.60120500
C	4.64536100	-0.67396900	-0.61255400
H	5.27516000	-0.68771500	-1.49751300
C	3.35481100	-1.21704900	-0.67924000
C	2.23043000	-0.66970000	2.99680200
H	1.23011700	-1.04234900	2.75954700
C	2.06154400	0.73451200	3.60467800
H	1.59982100	1.42475300	2.88927000
H	1.41688300	0.68554700	4.49085500
H	3.02144600	1.16245700	3.91769800
C	2.85094700	-1.63921700	4.02179700
H	3.85489400	-1.31484800	4.32184800

H	2.23068400	-1.68717100	4.92543500
H	2.93336100	-2.65410900	3.61507000
C	2.87104100	-1.85795000	-1.97705600
H	1.82370500	-2.14378500	-1.84622900
C	2.93556800	-0.88518800	-3.16830600
H	3.96581500	-0.58336900	-3.39164900
H	2.52997800	-1.36521600	-4.06741100
H	2.35014500	0.02130500	-2.97598800
C	3.66339000	-3.14529300	-2.27899400
H	3.59168800	-3.86455800	-1.45452600
H	3.27399000	-3.62841300	-3.18355600
H	4.72588200	-2.92986800	-2.44538400
C	0.09991800	-1.00502800	0.18167600
C	0.94667900	-3.06603900	0.70648600
H	1.71828800	-3.78367600	0.93887500
C	-0.39707200	-3.20134200	0.59356700
H	-1.03778600	-4.06226200	0.70700100
C	-2.30244400	-1.66404700	0.09764400
C	-3.07039900	-1.30972300	1.22757000
C	-4.43681500	-1.06561700	1.03347800
H	-5.05533300	-0.79364900	1.88405600
C	-5.01569400	-1.17247100	-0.22944600
H	-6.07767900	-0.97906300	-0.35777200
C	-4.23743800	-1.53550400	-1.32643100
H	-4.70076300	-1.62799900	-2.30460900
C	-2.86643700	-1.79351200	-1.18911000
C	-2.47871300	-1.21367600	2.63104900
H	-1.39730400	-1.35769600	2.55645200
C	-3.02899800	-2.33052800	3.53925000
H	-2.82744900	-3.32349700	3.12021100
H	-2.56088300	-2.28089400	4.53009700
H	-4.11326700	-2.23687200	3.67613900
C	-2.71042400	0.17105700	3.26327300
H	-3.77575200	0.36953100	3.43106900
H	-2.20498700	0.22982900	4.23501600
H	-2.31287900	0.97063600	2.62729200
C	-2.05549200	-2.22951500	-2.40569900
H	-1.00737600	-2.31373200	-2.10505200
C	-2.12354300	-1.19567200	-3.54465900
H	-1.77844400	-0.21088800	-3.20841900
H	-1.48500300	-1.51086500	-4.37896300
H	-3.14345400	-1.08298000	-3.93142400
C	-2.50723400	-3.61782600	-2.89836400
H	-3.54840400	-3.59962700	-3.24286600

H	-1.88281400	-3.94626700	-3.73833300
H	-2.42921700	-4.37025700	-2.10475100
C	-0.39794600	3.06499100	-0.36364200
C	-1.79402200	3.24791300	-0.27942400
C	0.47745900	3.65705300	0.57072000
C	-2.31118700	3.96319300	0.80594000
H	-2.45043500	2.85758500	-1.05029200
C	-0.07021200	4.36712200	1.64464000
C	-1.45575700	4.52567700	1.76021900
H	-3.38709700	4.09321800	0.89220200
H	0.59409200	4.81018200	2.38243800
Br	0.41396300	2.84893800	-2.42511200
H	1.55257900	3.57770100	0.44735700
H	-1.86822400	5.09818700	2.58633300

INT2

Pd	-0.49517000	0.99502300	-0.37392400
N	1.65381500	-1.08936900	0.88584300
C	2.79289200	-0.21553200	0.72510300
N	-0.33359800	-1.90223800	0.86954800
C	2.96214700	0.85017600	1.63385000
C	4.06631100	1.69363900	1.44721300
H	4.21626300	2.53172000	2.12175700
C	4.97972600	1.46635100	0.42058600
H	5.83240700	2.12886400	0.29591600
C	4.81068700	0.38214200	-0.43830400
H	5.54080600	0.20379000	-1.22215700
C	3.71559700	-0.48288900	-0.30916600
C	2.01470600	1.09856200	2.80449100
H	1.29363900	0.27718200	2.84109300
C	1.21939800	2.40297000	2.61697500
H	0.63611900	2.38019100	1.68676400
H	0.52408000	2.55352400	3.45173800
H	1.88470300	3.27406500	2.57232000
C	2.76318600	1.10095300	4.15078700
H	3.47252200	1.93377200	4.22245100
H	2.04878600	1.20250700	4.97679300
H	3.32085400	0.16875000	4.29963200
C	3.58437100	-1.68420900	-1.24228800
H	2.59752400	-2.12968800	-1.08676700
C	3.67800200	-1.29234200	-2.72764100
H	4.66157400	-0.87983300	-2.98218600
H	3.52173900	-2.17782600	-3.35622100

H	2.91376000	-0.55252800	-2.99030000
C	4.63402200	-2.75877600	-0.89502400
H	4.54802600	-3.08598100	0.14778800
H	4.50400000	-3.63928700	-1.53619800
H	5.65286200	-2.38049600	-1.04491500
C	0.38266600	-0.80848300	0.47035100
C	1.72206200	-2.32085600	1.53284800
H	2.64589500	-2.70547800	1.93644600
C	0.46741300	-2.83445300	1.52211000
H	0.07234200	-3.75910200	1.91367300
C	-1.75810800	-2.05261800	0.68273800
C	-2.62800200	-1.46011300	1.62290100
C	-4.00629200	-1.59238500	1.40477900
H	-4.70192200	-1.13736100	2.10401400
C	-4.49701100	-2.30647300	0.31377900
H	-5.56963100	-2.39965100	0.16426500
C	-3.61558500	-2.91343600	-0.57856300
H	-4.01006500	-3.48325200	-1.41511000
C	-2.22759900	-2.80275400	-0.41610700
C	-2.12735400	-0.71248600	2.85604300
H	-1.03853100	-0.80634900	2.89555600
C	-2.68227000	-1.32812500	4.15485000
H	-2.43371000	-2.39325900	4.23045100
H	-2.25284200	-0.81836200	5.02586000
H	-3.77250300	-1.23132400	4.21782200
C	-2.45476100	0.78925000	2.77696100
H	-3.53683500	0.96276800	2.72963500
H	-2.06851900	1.31284100	3.65981600
H	-2.00400700	1.24768500	1.88658000
C	-1.29452200	-3.50622200	-1.39800600
H	-0.26422300	-3.24829100	-1.13614700
C	-1.52691300	-3.04327000	-2.84765100
H	-1.37506900	-1.96256500	-2.94828800
H	-0.82003600	-3.54552000	-3.51975800
H	-2.53932700	-3.28408000	-3.19320700
C	-1.42632600	-5.03731500	-1.28184300
H	-2.43197300	-5.37566200	-1.55971400
H	-0.71047900	-5.53082100	-1.95056000
H	-1.22627000	-5.38148100	-0.26019800
C	-1.45002100	2.68513100	-0.82308000
C	-2.84688800	2.64686000	-0.93702100
C	-0.79341800	3.92332800	-0.80046000
C	-3.58438600	3.83799800	-0.96362200
H	-3.36959300	1.69360400	-0.99424800

C	-1.53387200	5.11217300	-0.82529300
C	-2.92925700	5.07177900	-0.90632700
H	-4.66920300	3.79874500	-1.03705700
H	-1.01714100	6.06907400	-0.79095000
Br	0.12015900	0.91204400	-2.86358900
H	0.29242900	3.97050300	-0.75784900
H	-3.50134600	5.99582800	-0.93597500

INT3

N	2.40153000	-0.69942400	-0.28038500
C	3.11680200	0.49709700	-0.68296500
N	0.90470200	-2.15977400	0.23055800
C	3.89208400	1.19306600	0.27349400
C	4.59607100	2.32422400	-0.16318500
H	5.19544500	2.88403800	0.54748000
C	4.55089200	2.73976000	-1.49121400
H	5.10253100	3.62291900	-1.80329600
C	3.82230800	2.00771200	-2.42420300
H	3.82594700	2.31708400	-3.46462400
C	3.10475800	0.86327400	-2.04838300
C	4.05233100	0.72194700	1.71733900
H	3.19596000	0.08719800	1.95998400
C	4.06921200	1.87806800	2.73240200
H	3.20755500	2.53770800	2.60123800
H	4.02776000	1.47349700	3.75090400
H	4.98690300	2.47384200	2.65862200
C	5.34170800	-0.11295000	1.87530100
H	6.22782200	0.49511200	1.65311400
H	5.43411800	-0.47531000	2.90691000
H	5.36068300	-0.98386400	1.21233000
C	2.44088000	0.01131700	-3.12713200
H	1.70252200	-0.63612100	-2.64637500
C	1.71154700	0.84553400	-4.19456000
H	2.41400000	1.39711500	-4.82992500
H	1.12949100	0.18700800	-4.85008100
H	1.02483800	1.57126700	-3.74544000
C	3.48556700	-0.90978900	-3.79275600
H	3.96859300	-1.56712300	-3.06106400
H	3.00745500	-1.54225000	-4.55106900
H	4.26830900	-0.32186800	-4.28772200
C	1.05633400	-0.82139600	-0.02757700
C	3.05430100	-1.92681700	-0.18339700
H	4.11225900	-2.02833100	-0.36420200

C	2.11626300	-2.84285400	0.14495800
H	2.18622100	-3.90530500	0.31616500
C	-0.33754500	-2.87279000	0.44815900
C	-0.88271600	-2.93481500	1.74910600
C	-2.07174200	-3.65946600	1.91684400
H	-2.52158800	-3.72603900	2.90200900
C	-2.67815900	-4.31388700	0.84638000
H	-3.59782100	-4.87193200	1.00271900
C	-2.09705900	-4.27133600	-0.41814600
H	-2.56543800	-4.80644000	-1.23876100
C	-0.91260100	-3.55705400	-0.64801000
C	-0.17716500	-2.32504100	2.95740700
H	0.44097400	-1.49386500	2.60917400
C	0.75364600	-3.37038900	3.60845900
H	1.49449600	-3.75348800	2.89757600
H	1.29574100	-2.92434700	4.45169700
H	0.18103400	-4.22551900	3.98972000
C	-1.14405800	-1.74888300	4.00386000
H	-1.72616900	-2.53059000	4.50673100
H	-0.57492600	-1.21849700	4.77544100
H	-1.84182300	-1.03276400	3.55722500
C	-0.26679900	-3.60017300	-2.03335000
H	0.50420900	-2.82511100	-2.07771300
C	-1.25782200	-3.32243800	-3.17849500
H	-1.77627600	-2.36709300	-3.04797600
H	-0.71649000	-3.28391900	-4.13154100
H	-2.01584400	-4.10935800	-3.26552300
C	0.42678400	-4.96023600	-2.25767300
H	-0.30482700	-5.77743400	-2.24597600
H	0.93183200	-4.97434900	-3.23127000
H	1.17578000	-5.16880500	-1.48591300
C	-2.10955800	-0.14956400	-1.27971600
H	-1.90129100	-1.16058900	-0.94249300
C	-1.14180400	0.48518500	-2.03745200
H	-1.34029800	1.43489600	-2.52453300
H	-0.31005700	-0.08881800	-2.42473900
C	-1.50278800	2.57110900	0.12550100
C	-1.11090600	3.62228900	-0.71748100
C	-1.79186300	4.84856700	-0.70724900
C	-2.87941600	5.04060800	0.14755300
C	-3.27664100	3.99958500	0.99370300
C	-2.59035300	2.77985700	0.98538500
H	-0.26799900	3.49757000	-1.39561200
H	-1.46938000	5.64944700	-1.37008300

H	-3.41129900	5.98909400	0.15598400
H	-4.12005800	4.13867800	1.66757900
H	-2.90863300	1.99131300	1.66254400
C	-3.50384600	0.27559000	-1.07383800
C	-4.11037300	1.28902000	-1.83962900
C	-4.28369100	-0.40362900	-0.11868800
C	-5.44797800	1.62381600	-1.63887300
H	-3.53883700	1.80573700	-2.60443300
C	-5.62040600	-0.06368500	0.08564800
H	-3.83168900	-1.19994700	0.46734600
C	-6.20642000	0.95358000	-0.67267800
H	-5.90332900	2.40482400	-2.24199800
H	-6.20538400	-0.59513700	0.83150300
H	-7.24984400	1.21637200	-0.52025200
Pd	-0.40186600	0.85427600	0.05919800
Br	0.39715300	1.50608800	2.45043400

TS2

C	-0.39468700	2.87519300	-0.74734600
H	0.11855800	3.04926600	-1.68889500
C	0.39772700	2.85943500	0.43346200
H	0.38264500	1.36379000	0.99131600
H	-0.09426100	3.15968500	1.35722300
C	1.85420500	3.18437100	0.40287100
C	2.70295900	2.77517000	-0.63954700
C	4.04524600	3.15197600	-0.64910700
C	4.56595500	3.93915500	0.38393600
C	3.73323800	4.34279200	1.42950100
C	2.38847100	3.96414700	1.44087900
H	2.31162900	2.14764200	-1.43593800
H	4.68864500	2.82395200	-1.46126300
H	5.61335700	4.22861500	0.37534500
H	4.12745600	4.95111400	2.23926700
H	1.74399100	4.28138700	2.25727100
C	-1.83711900	3.18452000	-0.75673000
C	-2.65980400	3.05726500	0.38094400
C	-2.42519600	3.64775100	-1.95000400
C	-4.01512200	3.37990600	0.32274200
H	-2.24124900	2.69773600	1.31610300
C	-3.77905600	3.97635000	-2.00477400
H	-1.80745600	3.75008700	-2.83807000
C	-4.58300100	3.84229000	-0.86878200
H	-4.62969800	3.26998200	1.21267100

H	-4.20721300	4.33780000	-2.93636800
H	-5.63886600	4.09619300	-0.91045500
N	-0.89339100	-2.01874700	0.88129100
C	-2.23238400	-1.58587100	1.21783800
N	1.17828000	-2.06403400	0.28373300
C	-3.30324300	-1.95168100	0.37397000
C	-4.59038300	-1.54023600	0.74796600
H	-5.43756100	-1.80402100	0.12225600
C	-4.80483900	-0.80884500	1.91356300
H	-5.81185100	-0.50020000	2.18255700
C	-3.73442200	-0.49065900	2.74633800
H	-3.91947300	0.05342400	3.66764400
C	-2.42653500	-0.88000900	2.42637000
C	-3.11519000	-2.82271300	-0.86574300
H	-2.05055700	-2.83185800	-1.11525200
C	-3.86426500	-2.28633600	-2.09719900
H	-3.55112600	-1.26519500	-2.33616400
H	-3.63956200	-2.91564100	-2.96728900
H	-4.95175600	-2.30106500	-1.95743700
C	-3.53994800	-4.27519600	-0.56637700
H	-4.60845000	-4.33112100	-0.32368100
H	-3.35962200	-4.91302800	-1.44063000
H	-2.98195600	-4.69558400	0.27803100
C	-1.29459400	-0.61122300	3.41558600
H	-0.34336600	-0.77948900	2.90413100
C	-1.27580400	0.83516600	3.93891500
H	-2.14960300	1.06010600	4.56150200
H	-0.38459000	0.99309500	4.55836700
H	-1.24887400	1.56094500	3.11845400
C	-1.36803600	-1.60798800	4.59056100
H	-1.31985000	-2.64585200	4.24094400
H	-0.53242800	-1.44511300	5.28242700
H	-2.30134800	-1.48507200	5.15379800
C	0.07956300	-1.25046100	0.30375700
C	-0.40877400	-3.28309200	1.21189700
H	-1.03172000	-4.03007700	1.67812100
C	0.89066000	-3.31192700	0.83616600
H	1.63601400	-4.08801100	0.91045300
C	2.52163300	-1.68520300	-0.10188500
C	2.95139300	-1.91132800	-1.42811000
C	4.27361300	-1.56775300	-1.74297500
H	4.63971100	-1.72252000	-2.75264400
C	5.13359400	-1.04047500	-0.78161600
H	6.15506200	-0.78384300	-1.05091600

C	4.69255700	-0.85653100	0.52582600
H	5.37867800	-0.46646100	1.27177400
C	3.38045600	-1.18001100	0.89896300
C	2.05550000	-2.56727500	-2.47609200
H	1.01611700	-2.35763300	-2.20808300
C	2.25633500	-4.09777200	-2.47487600
H	2.04626100	-4.53886700	-1.49452000
H	1.58715600	-4.56940300	-3.20535900
H	3.28763400	-4.35797100	-2.74529000
C	2.26969500	-2.01089600	-3.89335800
H	3.25037300	-2.28366300	-4.30148700
H	1.51116600	-2.42529800	-4.56750900
H	2.17075200	-0.92127800	-3.91618700
C	2.95794900	-1.04527600	2.36020900
H	1.87425700	-1.17808300	2.41926800
C	3.27524000	0.33913400	2.95033600
H	2.81005100	1.14052800	2.36653100
H	2.89258300	0.40309300	3.97644500
H	4.35348100	0.53269400	2.99013600
C	3.60366600	-2.15808500	3.21029500
H	4.69701800	-2.06868800	3.21578000
H	3.25552200	-2.09533500	4.24871800
H	3.34945500	-3.15397700	2.82942500
Pd	-0.13398700	0.69773000	-0.39098000
Br	-0.82141300	0.12487000	-2.87865200

INT4

C	-2.61960500	-0.74110600	-0.52573300
H	-2.80125000	-1.18475100	-1.50422900
C	-3.57128000	0.40913300	-0.21643800
H	-3.48286600	1.13813000	-1.02879600
H	-3.28718600	0.92176700	0.70843200
C	-5.01583400	-0.06163900	-0.10941900
C	-5.77526700	-0.30155300	-1.26537000
C	-7.09106900	-0.76117500	-1.17632800
C	-7.67290400	-0.98862600	0.07448200
C	-6.92839000	-0.75113000	1.23277100
C	-5.61120300	-0.29303800	1.13936000
H	-5.33275900	-0.12025500	-2.24278000
H	-7.66373200	-0.93732400	-2.08367400
H	-8.69786300	-1.34358200	0.14497700
H	-7.37311600	-0.91836300	2.21082500
H	-5.04088800	-0.10619000	2.04660900

C	-2.18530400	-1.69269300	0.48045900
C	-1.88318700	-1.27531700	1.80889100
C	-1.89469100	-3.04627400	0.12907200
C	-1.40300900	-2.19685800	2.75407000
H	-2.13023200	-0.26870900	2.12791900
C	-1.42924700	-3.94277800	1.07369600
H	-2.09028700	-3.37215500	-0.88937600
C	-1.19511900	-3.52402100	2.39830200
H	-1.21609700	-1.86757700	3.77217500
H	-1.25145000	-4.97808200	0.79532400
H	-0.84893100	-4.24016300	3.13816500
N	1.91311700	1.60835400	0.42588800
C	1.10672900	2.71348000	0.89973600
N	2.59951200	-0.32970700	-0.21387300
C	0.64051100	2.68352400	2.23536900
C	-0.11763800	3.77428400	2.68213800
H	-0.49576500	3.78211400	3.69923900
C	-0.37955000	4.86080700	1.85054900
H	-0.96738900	5.69732800	2.21987800
C	0.12587300	4.88430900	0.55405500
H	-0.06605200	5.74744300	-0.07557000
C	0.88225400	3.81747600	0.04726000
C	1.01268800	1.56658900	3.20982300
H	1.20816600	0.65832300	2.63279100
C	-0.09995800	1.23304100	4.21757200
H	-1.05764200	1.04279900	3.72099200
H	0.17348100	0.33555600	4.78469500
H	-0.25399800	2.03805700	4.94585400
C	2.31267300	1.92863700	3.95950100
H	2.17760000	2.83986100	4.55552900
H	2.59792400	1.11785400	4.64135400
H	3.14618600	2.09790600	3.26910000
C	1.47409900	3.91573200	-1.35710300
H	1.75928600	2.90872500	-1.67397900
C	0.47243000	4.45122800	-2.39488800
H	0.21302300	5.50102200	-2.21284900
H	0.91595600	4.39583400	-3.39658500
H	-0.44474900	3.85533700	-2.40538100
C	2.74188900	4.79650000	-1.35041000
H	3.49845100	4.42784600	-0.64954900
H	3.19241600	4.82126300	-2.35042600
H	2.49867500	5.82830900	-1.06689800
C	1.45088200	0.37327500	0.04751700
C	3.30543000	1.66679900	0.39515300

H	3.84805200	2.55792700	0.66751500
C	3.73641300	0.45126700	-0.01276300
H	4.73133400	0.06391700	-0.16577700
C	2.69048900	-1.74995400	-0.48051900
C	2.99167100	-2.60574700	0.60292300
C	3.09779600	-3.97849500	0.33743100
H	3.32615500	-4.66217200	1.14963500
C	2.92587800	-4.47695800	-0.95076200
H	3.00836300	-5.54503100	-1.13565000
C	2.66896300	-3.60677500	-2.00838600
H	2.56488600	-4.00799900	-3.01112100
C	2.55701000	-2.22427200	-1.80394700
C	3.27862700	-2.09001600	2.01265400
H	2.97501400	-1.04071000	2.06656200
C	4.79176900	-2.14911700	2.30629300
H	5.37118700	-1.57803300	1.57213200
H	5.00278100	-1.73484700	3.29991100
H	5.15509000	-3.18400300	2.28925500
C	2.49509100	-2.84323400	3.10019800
H	2.79623300	-3.89502700	3.17079100
H	2.68046500	-2.38311600	4.07862900
H	1.41794600	-2.81036700	2.90977900
C	2.38372900	-1.27997300	-2.99030700
H	1.86913300	-0.38262800	-2.63726600
C	1.52433100	-1.86760700	-4.12030700
H	0.56006800	-2.22933500	-3.74762300
H	1.31836800	-1.09104200	-4.86511000
H	2.02581000	-2.69359600	-4.63942400
C	3.76375600	-0.85614100	-3.53739800
H	4.32409000	-1.72480800	-3.90611600
H	3.64393000	-0.15325000	-4.37143000
H	4.37276800	-0.36462400	-2.77039000
Pd	-0.59998100	-0.17207900	-0.33480800
Br	-0.83446300	0.87645900	-2.72474800

TS3

C	-0.39468700	2.87519300	-0.74734600
H	0.11855800	3.04926600	-1.68889500
C	0.39772700	2.85943500	0.43346200
H	0.38264500	1.36379000	0.99131600
H	-0.09426100	3.15968500	1.35722300
C	1.85420500	3.18437100	0.40287100
C	2.70295900	2.77517000	-0.63954700

C	4.04524600	3.15197600	-0.64910700
C	4.56595500	3.93915500	0.38393600
C	3.73323800	4.34279200	1.42950100
C	2.38847100	3.96414700	1.44087900
H	2.31162900	2.14764200	-1.43593800
H	4.68864500	2.82395200	-1.46126300
H	5.61335700	4.22861500	0.37534500
H	4.12745600	4.95111400	2.23926700
H	1.74399100	4.28138700	2.25727100
C	-1.83711900	3.18452000	-0.75673000
C	-2.65980400	3.05726500	0.38094400
C	-2.42519600	3.64775100	-1.95000400
C	-4.01512200	3.37990600	0.32274200
H	-2.24124900	2.69773600	1.31610300
C	-3.77905600	3.97635000	-2.00477400
H	-1.80745600	3.75008700	-2.83807000
C	-4.58300100	3.84229000	-0.86878200
H	-4.62969800	3.26998200	1.21267100
H	-4.20721300	4.33780000	-2.93636800
H	-5.63886600	4.09619300	-0.91045500
N	-0.89339100	-2.01874700	0.88129100
C	-2.23238400	-1.58587100	1.21783800
N	1.17828000	-2.06403400	0.28373300
C	-3.30324300	-1.95168100	0.37397000
C	-4.59038300	-1.54023600	0.74796600
H	-5.43756100	-1.80402100	0.12225600
C	-4.80483900	-0.80884500	1.91356300
H	-5.81185100	-0.50020000	2.18255700
C	-3.73442200	-0.49065900	2.74633800
H	-3.91947300	0.05342400	3.66764400
C	-2.42653500	-0.88000900	2.42637000
C	-3.11519000	-2.82271300	-0.86574300
H	-2.05055700	-2.83185800	-1.11525200
C	-3.86426500	-2.28633600	-2.09719900
H	-3.55112600	-1.26519500	-2.33616400
H	-3.63956200	-2.91564100	-2.96728900
H	-4.95175600	-2.30106500	-1.95743700
C	-3.53994800	-4.27519600	-0.56637700
H	-4.60845000	-4.33112100	-0.32368100
H	-3.35962200	-4.91302800	-1.44063000
H	-2.98195600	-4.69558400	0.27803100
C	-1.29459400	-0.61122300	3.41558600
H	-0.34336600	-0.77948900	2.90413100
C	-1.27580400	0.83516600	3.93891500

H	-2.14960300	1.06010600	4.56150200
H	-0.38459000	0.99309500	4.55836700
H	-1.24887400	1.56094500	3.11845400
C	-1.36803600	-1.60798800	4.59056100
H	-1.31985000	-2.64585200	4.24094400
H	-0.53242800	-1.44511300	5.28242700
H	-2.30134800	-1.48507200	5.15379800
C	0.07956300	-1.25046100	0.30375700
C	-0.40877400	-3.28309200	1.21189700
H	-1.03172000	-4.03007700	1.67812100
C	0.89066000	-3.31192700	0.83616600
H	1.63601400	-4.08801100	0.91045300
C	2.52163300	-1.68520300	-0.10188500
C	2.95139300	-1.91132800	-1.42811000
C	4.27361300	-1.56775300	-1.74297500
H	4.63971100	-1.72252000	-2.75264400
C	5.13359400	-1.04047500	-0.78161600
H	6.15506200	-0.78384300	-1.05091600
C	4.69255700	-0.85653100	0.52582600
H	5.37867800	-0.46646100	1.27177400
C	3.38045600	-1.18001100	0.89896300
C	2.05550000	-2.56727500	-2.47609200
H	1.01611700	-2.35763300	-2.20808300
C	2.25633500	-4.09777200	-2.47487600
H	2.04626100	-4.53886700	-1.49452000
H	1.58715600	-4.56940300	-3.20535900
H	3.28763400	-4.35797100	-2.74529000
C	2.26969500	-2.01089600	-3.89335800
H	3.25037300	-2.28366300	-4.30148700
H	1.51116600	-2.42529800	-4.56750900
H	2.17075200	-0.92127800	-3.91618700
C	2.95794900	-1.04527600	2.36020900
H	1.87425700	-1.17808300	2.41926800
C	3.27524000	0.33913400	2.95033600
H	2.81005100	1.14052800	2.36653100
H	2.89258300	0.40309300	3.97644500
H	4.35348100	0.53269400	2.99013600
C	3.60366600	-2.15808500	3.21029500
H	4.69701800	-2.06868800	3.21578000
H	3.25552200	-2.09533500	4.24871800
H	3.34945500	-3.15397700	2.82942500
Pd	-0.13398700	0.69773000	-0.39098000
Br	-0.82141300	0.12487000	-2.87865200

INT5

C	-0.22484800	-3.10730100	0.35755100
H	-0.06052300	-3.17409600	1.42846000
C	0.86616900	-2.95011600	-0.46949100
H	0.71401100	-3.00899200	-1.54350900
C	2.28646500	-2.96866300	-0.07066500
C	2.72245800	-2.99676000	1.26913900
C	4.08105700	-3.06044000	1.57041100
C	5.03557400	-3.09946100	0.54725300
C	4.61781400	-3.07315000	-0.78526300
C	3.25790100	-3.00820600	-1.08936700
H	1.99934000	-2.94808800	2.07628400
H	4.39819500	-3.08129700	2.60991800
H	6.09389100	-3.15104100	0.78880400
H	5.34900600	-3.10529200	-1.58873700
H	2.93713200	-2.99278600	-2.12833700
C	-1.60119300	-3.43363500	-0.06497600
C	-1.96849000	-3.68410400	-1.40249000
C	-2.59157600	-3.55402400	0.92947100
C	-3.27819400	-4.03265600	-1.72731200
H	-1.22721900	-3.62120000	-2.19353600
C	-3.90248700	-3.89990800	0.60224600
H	-2.32368700	-3.37060100	1.96686000
C	-4.25266500	-4.13994000	-0.72878400
H	-3.53850900	-4.22825000	-2.76431900
H	-4.64868300	-3.98609600	1.38781900
H	-5.27193500	-4.41449400	-0.98689300
N	0.90976900	2.11997800	-0.40997000
C	2.30138300	1.76366700	-0.59540600
N	-1.24241800	2.04066800	-0.21619500
C	2.73936200	1.45055000	-1.90047100
C	4.09697200	1.14679600	-2.07180500
H	4.46704700	0.89966700	-3.06239200
C	4.98026800	1.16944500	-0.99583800
H	6.02991300	0.93298900	-1.15051500
C	4.52516700	1.50756100	0.27692400
H	5.23002000	1.53742600	1.10160900
C	3.17793000	1.81678000	0.50993600
C	1.81726300	1.48865000	-3.11644800
H	0.78695900	1.58249100	-2.76324900
C	1.89082300	0.20291700	-3.95943700
H	1.69953800	-0.68694400	-3.34955800
H	1.13587700	0.23602900	-4.75443600

H	2.86898300	0.08211600	-4.43952500
C	2.12078200	2.72508300	-3.98640300
H	3.14428700	2.69079500	-4.37943500
H	1.43379800	2.76929400	-4.84056200
H	2.01061000	3.65524000	-3.41688700
C	2.71781900	2.25436900	1.89783900
H	1.63976100	2.08265400	1.96411300
C	3.36811300	1.44718300	3.03345400
H	4.44412000	1.64238100	3.11565900
H	2.91222900	1.72875400	3.98994600
H	3.21559300	0.37212300	2.89865100
C	2.97703500	3.76219100	2.10023500
H	2.47828900	4.36885700	1.33626500
H	2.60554300	4.08428600	3.08111500
H	4.05077800	3.98510300	2.05814200
C	-0.12502100	1.25903200	-0.19367100
C	0.44275600	3.42419100	-0.56165500
H	1.11320100	4.24913900	-0.74335100
C	-0.90429200	3.37424200	-0.44174600
H	-1.65404200	4.14738600	-0.49597700
C	-2.61725700	1.59063700	-0.13262300
C	-3.24406300	1.13310100	-1.31235100
C	-4.58293500	0.72831600	-1.21865100
H	-5.09425000	0.36871100	-2.10660200
C	-5.27367600	0.79855700	-0.01188900
H	-6.31254900	0.48242200	0.03778500
C	-4.64241500	1.29255300	1.12739500
H	-5.20178000	1.36613400	2.05463600
C	-3.30333400	1.70570700	1.09637100
C	-2.55686500	1.14421200	-2.67568600
H	-1.48806900	1.31254100	-2.52232000
C	-3.08473200	2.31328100	-3.53213200
H	-2.93811300	3.27738500	-3.03123100
H	-2.55625300	2.34885900	-4.49290100
H	-4.15592300	2.20157500	-3.74034800
C	-2.70332700	-0.18853100	-3.42910500
H	-3.74453100	-0.39037000	-3.70678200
H	-2.11713800	-0.16040100	-4.35578500
H	-2.34567200	-1.03120400	-2.82787500
C	-2.66778300	2.32209500	2.33969600
H	-1.58190400	2.27223700	2.22235000
C	-3.01306100	1.56785300	3.63417400
H	-2.73885300	0.51120400	3.56289500
H	-2.45095500	2.00043900	4.47077500

H	-4.07764400	1.64323200	3.88550700
C	-3.07116800	3.80621100	2.46908000
H	-4.15408500	3.90544400	2.61637900
H	-2.57172200	4.26169000	3.33336200
H	-2.80046000	4.38635700	1.57989000
Pd	0.00984400	-0.74559700	0.07066700
H	-0.07289800	-0.72753300	-1.48566300
Br	0.05036000	-0.63208700	2.76316900

In [BMIM]BF$_4$ with Ez = +0.002 a.u. :

Pd(0)

Pd	0.00000000	0.00000000	0.00000000
C	0.00000000	0.00000000	1.96046700
H	5.55423100	0.00000000	3.20688500
N	0.80404600	-0.71922900	2.81758900
C	1.83502000	-1.64051300	2.40591900
N	-0.80345000	0.72067400	2.81701300
C	1.49933100	-2.99844000	2.22353500
C	2.52181600	-3.87604100	1.83871100
H	2.29168300	-4.92582600	1.67978300
C	3.82481099	-3.42334200	1.64511300
H	4.60137899	-4.11877900	1.33743300
C	4.13235299	-2.07878301	1.84024800
H	5.15075999	-1.73473301	1.68276200
C	3.14891000	-1.15863701	2.22765300
C	0.08436699	-3.53276100	2.42447800
H	-0.56327801	-2.69237600	2.68974500
C	-0.47704001	-4.14464000	1.12812800
H	-0.46727101	-3.40889900	0.31585700
H	-1.51388201	-4.46946800	1.27914800
H	0.10178999	-5.01804300	0.80524600
C	0.02648599	-4.54244800	3.58595700
H	0.65048399	-5.42221901	3.38746500
H	-1.00274201	-4.89177600	3.73577400
H	0.37057699	-4.09232200	4.52557400
C	3.52544100	0.30691299	2.42396300
H	2.63714800	0.84364199	2.76922500
C	3.96387700	0.95340100	1.09633599
H	4.86157900	0.47163399	0.69058799
H	4.19234000	2.01582799	1.24705099
H	3.16882099	0.88077200	0.34505099

C	4.61153800	0.47530499	3.50315100
H	4.29950200	0.03684299	4.45929600
H	4.81658400	1.53992300	3.67080600
C	0.50447400	-0.45099700	4.15155499
H	1.03504300	-0.92579300	4.96252300
C	-0.50330800	0.45409900	4.15119000
H	-1.03348400	0.92991400	4.96181299
C	-1.83101800	1.64539300	2.40439800
C	-3.14657899	1.16854600	2.22541400
C	-4.12633699	2.09229900	1.83719100
H	-5.14598799	1.75206700	1.67946800
C	-3.81364099	3.43557199	1.64161000
H	-4.58739799	4.13383099	1.33324900
C	-2.50902299	3.88333399	1.83569200
H	-2.27480999	4.93217799	1.67651300
C	-1.49010999	3.00202300	2.22147400
C	-3.52923299	-0.29523100	2.42282400
H	-2.64293800	-0.83587100	2.76712600
C	-4.61475399	-0.45743800	3.50350200
H	-4.29820599	-0.02224600	4.45966300
H	-4.82702099	-1.52078100	3.67026500
H	-5.55434199	0.02508300	3.20905900
C	-3.97250099	-0.94051300	1.09620100
H	-4.86811300	-0.45445000	0.69097500
H	-4.20626399	-2.00160901	1.24811100
H	-3.17790000	-0.87268500	0.34399100
C	-0.07338099	3.53138300	2.42327500
H	0.57136101	2.68874000	2.68845700
C	0.49082201	4.14219200	1.12764400
H	0.48040501	3.40658700	0.31525800
H	1.52820801	4.46465900	1.27998300
H	-0.08564900	5.01694200	0.80424400
C	-0.01280499	4.54028100	3.58531600
H	-0.63362399	5.42228100	3.38675300
H	1.01752100	4.88595700	3.73608500
H	-0.35915699	4.09093200	4.52447200

INT1

Pd	0.00000000	0.00000000	0.00000000
C	0.00000000	0.00000000	2.05869500
C	0.66204700	0.00000000	4.25253600
N	1.06416900	0.09342500	2.92275300
C	2.43943400	0.28380500	2.52767600

N	-1.07242200	-0.15506400	2.90332400
C	2.92634400	1.59975900	2.37815900
C	4.27255400	1.75721900	2.02261700
H	4.67687400	2.75728500	1.89475700
C	5.10018300	0.65370800	1.82637800
H	6.13979200	0.79862000	1.54412500
C	4.59657300	-0.63463400	1.98935200
H	5.25044000	-1.48830701	1.83496300
C	3.25812600	-0.84966600	2.34551600
C	2.05729300	2.83152900	2.61617100
H	1.02530600	2.49758000	2.75405600
C	2.06395400	3.79098700	1.41314300
H	1.72722300	3.28381900	0.50277901
H	1.38645500	4.63333400	1.59973100
H	3.06117000	4.20515900	1.22382800
C	2.48156900	3.56261600	3.90509600
H	3.50839500	3.94155699	3.83022600
H	1.82103800	4.41840300	4.09252300
H	2.43115600	2.89976700	4.77781300
C	2.74791400	-2.27446000	2.53978600
H	1.67250101	-2.22652700	2.73228400
C	2.94587600	-3.13438300	1.27882600
H	4.00686700	-3.26667500	1.03635200
H	2.51355400	-4.13127400	1.42936800
H	2.45324000	-2.68092600	0.41203101
C	3.40806900	-2.93443300	3.76601500
H	3.23246900	-2.35244200	4.67929400
H	3.00053000	-3.94088101	3.92320000
H	4.49257400	-3.03015600	3.63248500
H	1.36530600	0.05421400	5.06918600
C	-0.68415000	-0.15640600	4.24019100
H	-1.39615300	-0.26489900	5.04375900
C	-2.44637400	-0.28581400	2.48034300
C	-3.21552300	0.88252700	2.29999200
C	-4.55339300	0.72640900	1.91287600
H	-5.16923500	1.60801500	1.75835200
C	-5.10464301	-0.53808100	1.71956100
H	-6.14325400	-0.63659000	1.41463500
C	-4.32518601	-1.67653900	1.91241800
H	-4.76531701	-2.65749600	1.75810900
C	-2.98075900	-1.57815600	2.29532500
C	-2.65406000	2.28365200	2.52214700
H	-1.59318700	2.19078500	2.77041800
C	-3.34647700	2.97587600	3.71192400

H	-3.24653500	2.38764500	4.63260400
H	-2.90036100	3.96210400	3.89113300
H	-4.41660400	3.12399500	3.52243800
C	-2.74878700	3.14658100	1.25069600
H	-3.78941400	3.31404000	0.94862300
H	-2.29138800	4.12833199	1.42545200
H	-2.22420800	2.67338600	0.41313300
C	-2.16211600	-2.84715500	2.51368700
H	-1.12188800	-2.55609500	2.68515800
C	-2.17868500	-3.76676300	1.27964000
H	-1.81675200	-3.24046800	0.38978000
H	-1.52767100	-4.63343300	1.44762300
H	-3.18456901	-4.14530400	1.06398300
C	-2.64143900	-3.60375500	3.76764400
H	-3.68061000	-3.93858100	3.66002500
H	-2.01983600	-4.49173400	3.93728700
H	-2.58275400	-2.97430900	4.66430200
C	-0.13544100	-0.22071300	-2.15044800
C	-1.46370900	0.18450500	-2.43032700
C	0.91957900	0.72224600	-2.10933400
C	-1.71860700	1.52876100	-2.69032500
H	-2.25802000	-0.55188200	-2.49018400
C	0.62094900	2.07730600	-2.37654400
C	-0.67702800	2.47200200	-2.68250200
H	-2.73379499	1.83854699	-2.92267400
H	1.43325001	2.79905200	-2.38515800
Br	0.31946400	-2.16502500	-2.43388500
H	1.95189801	0.39868400	-2.03353400
H	-0.88671900	3.51022101	-2.92325000

TS1

Pd	0.00000000	0.00000000	0.00000000
C	0.00000000	0.00000000	2.09278000
N	1.06763700	0.00000000	2.95524200
C	2.45188000	-0.04834500	2.54967100
N	-1.08167400	0.04717500	2.93632100
C	3.12621000	1.16217100	2.28462500
C	4.47301000	1.08568900	1.90524900
H	5.01883101	1.99963200	1.68848800
C	5.12222300	-0.14208000	1.79605000
H	6.16508600	-0.17900800	1.49203200
C	4.43617200	-1.32215101	2.07364100
H	4.95208900	-2.27400401	1.98523000

C	3.08852701	-1.30357201	2.45803300
C	2.45605300	2.52583300	2.42259700
H	1.39462100	2.36467700	2.63115800
C	2.54367200	3.34886100	1.12500200
H	2.10034100	2.80645000	0.28260401
H	2.00018100	4.29452500	1.24170000
H	3.58015600	3.59328500	0.86431200
C	3.04580900	3.30966600	3.61132000
H	4.11271300	3.51663600	3.46272000
H	2.53153200	4.27186600	3.72679800
H	2.93958400	2.75394700	4.55126700
C	2.37556200	-2.61605401	2.77256400
H	1.32864601	-2.39064301	2.99423500
C	2.39042500	-3.57958501	1.57216000
H	3.40982300	-3.88397901	1.30691700
H	1.82337000	-4.48837801	1.80914000
H	1.93425901	-3.11808201	0.68962800
C	2.97680801	-3.28503501	4.02378300
H	2.92670701	-2.62244301	4.89687400
H	2.42940601	-4.20445401	4.26644200
H	4.02830400	-3.55462701	3.86724300
C	0.65923000	0.04740700	4.28621200
H	1.36509600	0.05423800	5.10254500
C	-0.69568700	0.07642700	4.27418600
H	-1.41456500	0.11192300	5.07831400
C	-2.45844200	0.06914200	2.50379000
C	-3.07860600	1.31518300	2.27313300
C	-4.42054700	1.30821500	1.86986300
H	-4.92531200	2.25140400	1.68082300
C	-5.11588500	0.11266800	1.70154000
H	-6.15420600	0.12939800	1.38074100
C	-4.48116600	-1.10426600	1.93989300
H	-5.03210800	-2.03069599	1.80426700
C	-3.14078600	-1.15508900	2.34595700
C	-2.35337401	2.64353400	2.46724300
H	-1.30116201	2.42916800	2.67494900
C	-2.91738801	3.40553200	3.68210900
H	-2.83811501	2.81255000	4.60163900
H	-2.36647101	4.34194600	3.83483300
H	-3.97454501	3.65970400	3.53732500
C	-2.39812401	3.52068400	1.20289900
H	-3.42074601	3.82807400	0.95441800
H	-1.80681900	4.43159900	1.35697300
H	-1.98478201	2.99166000	0.33710000

C	-2.48320300	-2.50547000	2.61575800
H	-1.42813100	-2.33070500	2.84422400
C	-2.53430600	-3.42787000	1.38440000
H	-2.05835600	-2.95607700	0.51784700
H	-2.00234300	-4.36445400	1.59205200
H	-3.56371500	-3.68506200	1.10844900
C	-3.11381700	-3.19052100	3.84347800
H	-4.17579900	-3.40858800	3.67749500
H	-2.60706500	-4.14067700	4.05364800
H	-3.03612500	-2.56136200	4.73885800
C	-0.07405500	0.25939200	-2.02824100
C	-1.41302900	0.56515900	-2.34254500
C	0.98021100	1.12742000	-2.37558700
C	-1.69469800	1.80096700	-2.93445700
H	-2.20477100	-0.15097700	-2.14914400
C	0.66692200	2.35542800	-2.96739600
C	-0.66156800	2.68945200	-3.25154400
H	-2.72505500	2.05474800	-3.16996300
H	1.47006900	3.03968600	-3.22862300
Br	0.44532500	-1.91611000	-2.04604500
H	2.01234301	0.83868300	-2.20706400
H	-0.88959000	3.63245000	-3.74018100

INT2

Pd	0.00000000	0.00000000	0.00000000
C	0.00000000	0.00000000	2.17388300
N	1.02106100	0.00000000	3.08337500
C	2.42633700	-0.07300500	2.75576700
N	-1.11950700	0.12117100	2.94980100
C	3.07993500	1.09254600	2.30569200
C	4.44277800	0.99307600	1.99250200
H	4.96958300	1.86978200	1.62668701
C	5.12995100	-0.20832001	2.14224101
H	6.18569600	-0.26465101	1.89017201
C	4.46601400	-1.33850900	2.61488800
H	5.01439100	-2.26848500	2.73145200
C	3.10132200	-1.30030400	2.92887400
C	2.37453300	2.43719500	2.15729500
H	1.35016600	2.33108400	2.52617200
C	2.29243700	2.86306000	0.68029800
H	1.77582300	2.10346500	0.07921000
H	1.74496000	3.80789500	0.58123200
H	3.29026000	3.00549900	0.24832100
C	3.05055700	3.52832499	3.00817700

H	4.07662199	3.72581999	2.67671200
H	2.49111200	4.46839600	2.92904300
H	3.08697200	3.24483100	4.06730400
C	2.41093100	-2.54966500	3.47088001
H	1.33102400	-2.37707501	3.44724601
C	2.68740800	-3.79921101	2.61666500
H	3.74723700	-4.07970601	2.62818000
H	2.11889500	-4.65147701	3.00939800
H	2.38149600	-3.64030301	1.57763801
C	2.80910700	-2.79653300	4.94022801
H	2.57079100	-1.93473501	5.57547901
H	2.27530900	-3.66826101	5.33933601
H	3.88519200	-2.99154801	5.03012201
C	0.54656500	0.12750800	4.38689200
H	1.21042900	0.15664900	5.23688500
C	-0.80449899	0.20333000	4.30249400
H	-1.56216799	0.31109000	5.06316000
C	-2.46262299	0.20334700	2.42308200
C	-2.93151799	1.45092500	1.96028700
C	-4.22322299	1.49738500	1.41777700
H	-4.60780999	2.43843900	1.03527100
C	-5.02160699	0.35766600	1.36203100
H	-6.01880299	0.41466100	0.93380500
C	-4.54677799	-0.85419800	1.85909900
H	-5.18368499	-1.73297000	1.81946900
C	-3.25836100	-0.96174900	2.39916500
C	-2.10105699	2.72895000	2.04236000
H	-1.17892499	2.50412600	2.58659200
C	-2.83498499	3.82944000	2.83213400
H	-3.11457899	3.48478700	3.83524200
H	-2.18790399	4.70796500	2.94360300
H	-3.74814899	4.15499800	2.32103900
C	-1.70154799	3.23446100	0.64416300
H	-2.58279099	3.48015800	0.03973100
H	-1.08543199	4.13819600	0.72286600
H	-1.12581699	2.47779900	0.09470600
C	-2.78144900	-2.29765200	2.96373100
H	-1.71494800	-2.21055100	3.19161100
C	-2.93708900	-3.45298900	1.95963700
H	-2.38412700	-3.25190300	1.03607200
H	-2.53962600	-4.37939400	2.39275700
H	-3.98692000	-3.63497900	1.70193300
C	-3.51266600	-2.61803300	4.28289200
H	-4.59135999	-2.73134200	4.11891600

H	-3.13848000	-3.55705200	4.70969700
H	-3.36922300	-1.82596900	5.02823200
C	-0.09495500	0.27234000	-1.96647600
C	-1.36077300	0.42143200	-2.54797000
C	1.05580500	0.54763800	-2.71595400
C	-1.47550100	0.91937400	-3.85240100
H	-2.25959900	0.16031000	-1.99220000
C	0.93464800	1.04601800	-4.01937400
C	-0.32888801	1.23311700	-4.58751700
H	-2.46010500	1.04717400	-4.29637800
H	1.82923600	1.27387700	-4.59437999
Br	0.37128900	-2.47582500	-0.53172800
H	2.04486800	0.38181400	-2.29493800
H	-0.41873701	1.60477800	-5.60476000

INT3

Pd	0.00000000	0.00000000	0.00000000
C	0.00000000	0.00000000	2.23203300
N	1.11996600	0.00000000	3.02919300
C	2.49322300	-0.21535600	2.61225100
N	-1.02563100	0.14226300	3.13199600
C	3.37163700	0.88863100	2.52374900
C	4.69945300	0.63143100	2.15426500
H	5.39565100	1.45863700	2.06226900
C	5.14420600	-0.66222700	1.89844500
H	6.17542100	-0.83384200	1.60095700
C	4.27170401	-1.73763900	2.03743100
H	4.63597500	-2.74432800	1.86011300
C	2.93506101	-1.54334800	2.41078400
C	2.95074400	2.31487100	2.87100400
H	1.86590300	2.38129400	2.75253200
C	3.57099800	3.37178600	1.94128500
H	3.39337900	3.13271300	0.88990401
H	3.11419700	4.34858200	2.14034700
H	4.65049400	3.47992500	2.10206000
C	3.30858000	2.64896800	4.33549301
H	4.39411000	2.60514200	4.49216701
H	2.97614500	3.66516300	4.58294301
H	2.84081300	1.96187701	5.04873800
C	2.04505601	-2.75560000	2.67480000
H	1.00301301	-2.43165700	2.60623600
C	2.24935101	-3.89510100	1.66160100
H	3.22234301	-4.38427700	1.78633099

H	1.48120301	-4.66464400	1.80341900
H	2.18399301	-3.53869900	0.62835599
C	2.26332501	-3.27649000	4.11109700
H	2.05694601	-2.50101000	4.85770999
H	1.59836801	-4.12558099	4.31403800
H	3.29698201	-3.61617300	4.25238100
C	0.79057100	0.13662200	4.37592400
H	1.54198200	0.14186200	5.14875500
C	-0.55637900	0.23305500	4.44126400
H	-1.22295800	0.34952800	5.28091200
C	-2.44398001	0.07598100	2.84546800
C	-3.14062501	1.25752900	2.51167500
C	-4.51773501	1.14836400	2.27031200
H	-5.08362101	2.03342900	1.99867000
C	-5.17710801	-0.07410900	2.37571600
H	-6.24441301	-0.13281400	2.17864000
C	-4.47320701	-1.21843099	2.74161300
H	-5.00226001	-2.16189699	2.83495500
C	-3.09430701	-1.17169099	2.98898500
C	-2.46090001	2.62359201	2.48136000
H	-1.40313101	2.47286401	2.25038900
C	-2.55802801	3.29656801	3.86713400
H	-2.10735801	2.67985201	4.65346600
H	-2.03607001	4.26153600	3.85779200
H	-3.60441801	3.48227501	4.14230400
C	-3.02099401	3.56009000	1.39915300
H	-4.04673501	3.88137200	1.61754200
H	-2.40255801	4.46242500	1.33892000
H	-3.01085900	3.08778300	0.41207500
C	-2.36739501	-2.43264899	3.45807000
H	-1.29201701	-2.27594799	3.32723700
C	-2.74454701	-3.69292099	2.65902900
H	-2.59950601	-3.55393599	1.58344600
H	-2.11577501	-4.53361599	2.97647000
H	-3.78727501	-3.98740799	2.82387000
C	-2.62473802	-2.67000399	4.96084400
H	-3.68980902	-2.85595099	5.14796600
H	-2.06370801	-3.54536299	5.31147000
H	-2.32316101	-1.80897299	5.56774400
C	-1.65463100	-1.69791900	-0.38733300
H	-2.21816400	-1.47132000	0.51312500
C	-0.37850800	-2.21013000	-0.22874000
H	0.18442500	-2.58835300	-1.07607000
H	-0.06108500	-2.55245200	0.74817700

C	0.32542300	0.11165500	-2.00456000
C	1.48672700	-0.49679400	-2.50024700
C	1.80231600	-0.42974600	-3.86508599
C	0.95980600	0.24839400	-4.74791799
C	-0.19660700	0.86261900	-4.25745299
C	-0.50871699	0.79817100	-2.89470100
H	2.15961700	-1.03236400	-1.83217800
H	2.70635800	-0.91036000	-4.23347699
H	1.20085500	0.29913500	-5.80660499
H	-0.85720899	1.39782899	-4.93625099
H	-1.40811099	1.29021500	-2.53405000
C	-2.45064100	-1.63264800	-1.62064200
C	-2.07020200	-2.28169700	-2.81061700
C	-3.67729500	-0.94195000	-1.59278200
C	-2.88327800	-2.22303700	-3.93965100
H	-1.13965600	-2.83893600	-2.85211700
C	-4.48709700	-0.87840200	-2.72546600
H	-3.98832000	-0.44826299	-0.67503900
C	-4.09095500	-1.51754700	-3.90352900
H	-2.57711000	-2.72951400	-4.85059100
H	-5.42693300	-0.33419899	-2.69036100
H	-4.72105000	-1.47288500	-4.78744801
Br	0.51594600	2.55671900	0.04729500

TS2

Pd	0.00000000	0.00000000	0.00000000
C	0.00000000	0.00000000	2.13009800
N	1.16451200	0.00000000	2.86094300
C	2.50168500	-0.27792500	2.36967900
N	-0.97626000	0.18300300	3.07688900
C	3.42734000	0.78074900	2.23121100
C	4.71318800	0.46094400	1.77046700
H	5.44244100	1.25386700	1.63941600
C	5.07498600	-0.84996701	1.47824800
H	6.07376800	-1.06990101	1.11034700
C	4.16324500	-1.88321801	1.67863800
H	4.46761100	-2.90561001	1.48035800
C	2.86684300	-1.62692101	2.14315400
C	3.11851600	2.22140499	2.63200400
H	2.03236900	2.33881199	2.66629600
C	3.65253600	3.25698999	1.62781600
H	3.28631400	3.05531499	0.61830400
H	3.30288100	4.25692199	1.91179700

H	4.74868800	3.28784199	1.61124300
C	3.69271800	2.51962799	4.03398400
H	4.78753700	2.44293599	4.03114100
H	3.42989000	3.53924099	4.34342200
H	3.31452800	1.82928199	4.79622700
C	1.94376100	-2.79556901	2.48213500
H	0.91193100	-2.44046801	2.42339900
C	2.07769200	-3.99094301	1.52289300
H	3.03260200	-4.51424601	1.64960800
H	1.28253100	-4.71823901	1.72460100
H	1.99878100	-3.68784301	0.47347700
C	2.18279900	-3.26008501	3.93480100
H	2.02462700	-2.44692501	4.65220800
H	1.49524200	-4.07602701	4.19141400
H	3.20791400	-3.63003701	4.06305900
C	0.91265900	0.20593800	4.21519100
H	1.70508900	0.23865600	4.94527300
C	-0.42639700	0.32750100	4.35006800
H	-1.04200000	0.49485700	5.21913600
C	-2.40909400	0.02801400	2.90840500
C	-3.20057000	1.14440100	2.56506800
C	-4.58373900	0.94443700	2.44704300
H	-5.22041299	1.77844900	2.17047400
C	-5.15853500	-0.30049100	2.68715600
H	-6.23253499	-0.43188300	2.58295000
C	-4.36102100	-1.37339600	3.07556200
H	-4.82334600	-2.33378500	3.28179800
C	-2.97279900	-1.23534100	3.20412600
C	-2.61335300	2.54343000	2.41599000
H	-1.56164700	2.44649800	2.13592100
C	-2.68071800	3.28958200	3.76520699
H	-2.13800600	2.75470500	4.55392200
H	-2.23482600	4.28766300	3.66992799
H	-3.71952800	3.41538000	4.09786700
C	-3.28676100	3.37687801	1.31442299
H	-4.32002900	3.64650601	1.56573799
H	-2.73092300	4.30954101	1.16978399
H	-3.29419300	2.84612201	0.35698399
C	-2.14793099	-2.41121600	3.72848200
H	-1.09345500	-2.21523400	3.51349000
C	-2.50460799	-3.75412200	3.06950900
H	-2.45116699	-3.69813900	1.97865000
H	-1.80351099	-4.52811801	3.40576900
H	-3.51285599	-4.08990900	3.33948700

C	-2.29652299	-2.52874600	5.26059500
H	-3.33792199	-2.73879000	5.53501900
H	-1.67621300	-3.34986100	5.64179300
H	-1.99277799	-1.61011800	5.77404401
C	-0.56415700	-2.06778800	-0.33118100
H	-0.09324200	-2.58855501	0.49697900
C	0.24709500	-1.82311900	-1.46960800
H	-0.17699100	-1.97535800	-2.45427900
H	1.31435400	-2.01608301	-1.40124900
C	0.31492600	0.20856200	-2.10990700
C	1.61527199	0.48958100	-2.54985600
C	1.82021699	1.17066100	-3.75291000
C	0.72982400	1.56403300	-4.53396900
C	-0.56819700	1.26355100	-4.10923500
C	-0.77589800	0.57345500	-2.91215499
H	2.47283399	0.18488100	-1.95523000
H	2.83343400	1.38911300	-4.08230500
H	0.89005400	2.08514400	-5.47399000
H	-1.42127101	1.55208700	-4.71890200
H	-1.78828900	0.31443600	-2.61446500
C	-2.02057201	-2.36952300	-0.53985100
C	-3.01520501	-1.38326700	-0.59787700
C	-2.39818801	-3.70992900	-0.74259700
C	-4.34573301	-1.72164200	-0.86200300
H	-2.74044601	-0.34519200	-0.43493200
C	-3.72658001	-4.04963300	-1.00603400
H	-1.63951201	-4.48879200	-0.71172800
C	-4.70618401	-3.05451001	-1.06836300
H	-5.09868801	-0.93931600	-0.90863700
H	-3.99341501	-5.09010000	-1.17310900
H	-5.73912901	-3.31617101	-1.28200700
Br	0.34379100	2.64907900	0.14525100

INT4

Pd	0.00000000	0.00000000	0.00000000
C	0.00000000	0.00000000	2.15074900
C	0.12937100	0.00000000	-2.10146100
H	-0.78938200	0.39751500	-2.53149300
C	0.49419500	-1.37211600	-2.65457800
H	-0.35961800	-2.03561600	-2.48286800
H	1.34790000	-1.80008700	-2.11791600
C	0.80805600	-1.31887500	-4.14469200
C	-0.22797300	-1.31179600	-5.09096600

C	0.05124501	-1.24287000	-6.45761400
C	1.37503300	-1.17988100	-6.90168300
C	2.41507500	-1.18766800	-5.96905500
C	2.13185100	-1.25561100	-4.60242101
H	-1.26132499	-1.36884400	-4.75488300
H	-0.76398000	-1.24413200	-7.17654101
H	1.59330800	-1.12996100	-7.96490000
H	3.44789700	-1.14582100	-6.30558100
H	2.94879500	-1.26706600	-3.88395701
C	1.12481000	1.01206600	-1.79849300
C	2.38600300	0.66422200	-1.23502600
C	0.81573700	2.39979599	-1.92776400
C	3.31853700	1.65996900	-0.90621800
H	2.67270800	-0.37936800	-1.16443700
C	1.74903500	3.36916599	-1.60699400
H	-0.15189600	2.68029099	-2.33538600
C	3.01366200	3.00122000	-1.10888900
H	4.29251800	1.37233400	-0.52064000
H	1.51112400	4.41865499	-1.75686600
H	3.75075799	3.76806000	-0.88754700
N	0.26972000	-1.06970700	2.96794600
C	0.86075300	-2.32823400	2.56381400
N	-0.45693200	0.95662000	3.02206300
C	2.25937499	-2.38213200	2.35901700
C	2.81833499	-3.62051399	2.01732700
H	3.88658899	-3.69542099	1.84298500
C	2.02822199	-4.76163100	1.90056600
H	2.48288399	-5.71110800	1.63006000
C	0.65814399	-4.68703400	2.13161900
H	0.05498299	-5.58493600	2.04229900
C	0.04051899	-3.47364599	2.46751200
C	3.16510599	-1.16960800	2.56966400
H	2.57400899	-0.26899800	2.38271300
C	4.36611299	-1.12699100	1.60995600
H	4.05666499	-1.24074700	0.56595900
H	4.88220799	-0.16511500	1.70967300
H	5.10003299	-1.91087900	1.83142700
C	3.65653299	-1.11148200	4.03165100
H	4.25725599	-1.99681500	4.27646400
H	4.28430699	-0.22514800	4.18795600
H	2.82226199	-1.06361500	4.74066600
C	-1.45929401	-3.44496399	2.75412900
H	-1.79659501	-2.40824899	2.66821600
C	-2.27748002	-4.26874799	1.74437100

H	-2.09213502	-5.34534100	1.84224100
H	-3.34752802	-4.10457499	1.91897400
H	-2.06075601	-3.96429899	0.71709200
C	-1.75276701	-3.93636999	4.18747701
H	-1.24354302	-3.33287700	4.94723501
H	-2.83011502	-3.89298599	4.39136601
H	-1.42965202	-4.97731999	4.31691101
C	-0.01205300	-0.78054000	4.30175300
H	0.14659800	-1.50031900	5.08866700
C	-0.47344300	0.49096700	4.33555300
H	-0.80101400	1.10767200	5.15755300
C	-0.72711300	2.34110000	2.69743000
C	0.31390000	3.27777300	2.88091000
C	0.04297500	4.61977500	2.58004400
H	0.82546900	5.36321700	2.70047600
C	-1.21305800	5.01464000	2.12856500
H	-1.40140200	6.05812100	1.88929900
C	-2.23379200	4.07627800	1.99279600
H	-3.21186500	4.40372500	1.65637200
C	-2.02196000	2.72119800	2.28211600
C	1.67719900	2.89513700	3.45447700
H	1.76537800	1.80520100	3.43602400
C	1.78334000	3.34097300	4.92721700
H	0.98585200	2.90465100	5.53985100
H	2.74572500	3.02866700	5.35209000
H	1.71531200	4.43254200	5.01424900
C	2.85193200	3.45557600	2.63742100
H	2.88638900	4.55110599	2.66141700
H	3.80138000	3.08897199	3.04734800
H	2.79223800	3.14166199	1.59125000
C	-3.17760100	1.72682800	2.21884000
H	-2.76201000	0.73765100	2.00996600
C	-4.18506900	2.02729000	1.09830600
H	-3.68942500	2.12714100	0.12795300
H	-4.90001500	1.20114900	1.01860000
H	-4.76131800	2.94116100	1.28892600
C	-3.90333300	1.66363700	3.58000800
H	-4.35217600	2.63304300	3.83297000
H	-4.70779000	0.91819300	3.54684000
H	-3.22343200	1.38609700	4.39389600
Br	-2.37063500	-1.08193400	-0.27493800

TS3

Pd	0.00000000	0.00000000	0.00000000
C	0.00000000	0.00000000	2.07476300
C	0.29896800	0.00000000	-2.19268700
H	-0.55826300	-0.55150500	-2.56919800
C	1.41621400	-0.76110100	-1.74871000
H	1.51562800	-0.55195100	-0.16833900
H	2.39284500	-0.28745900	-1.83274400
C	1.45951500	-2.24440400	-1.90465800
C	0.34337500	-3.06593000	-1.67546400
C	0.41511600	-4.43830000	-1.90952600
C	1.60362600	-5.01594100	-2.36901700
C	2.72242900	-4.20994700	-2.58721900
C	2.65219300	-2.83494400	-2.35149400
H	-0.57835000	-2.62671800	-1.30324500
H	-0.45900400	-5.05881800	-1.73150400
H	1.65493600	-6.08503100	-2.55651400
H	3.64974800	-4.64703500	-2.94752100
H	3.52329600	-2.21047800	-2.53522300
C	0.39544700	1.39580900	-2.65829900
C	1.47865100	2.24098900	-2.34342800
C	-0.62715600	1.90545900	-3.48176400
C	1.53543500	3.54212900	-2.84059700
H	2.28234700	1.88127100	-1.70712900
C	-0.56574800	3.20477300	-3.98200799
H	-1.46951200	1.26785600	-3.73474300
C	0.51589500	4.03082400	-3.66342599
H	2.38285800	4.17538400	-2.59049100
H	-1.36040100	3.57103800	-4.62646399
H	0.56709400	5.04210300	-4.05747499
N	0.22945400	1.06944200	2.89680800
C	0.62456300	2.39432600	2.46797500
N	-0.23474900	-1.03609500	2.93604400
C	-0.35191400	3.41057100	2.38690600
C	0.07581600	4.68578500	1.99298600
H	-0.64855300	5.48958300	1.90925000
C	1.41307100	4.94161600	1.69953500
H	1.71865300	5.93674601	1.38692300
C	2.35940200	3.92650900	1.81000400
H	3.40109100	4.14186600	1.59209100
C	1.99067500	2.63252400	2.20157600
C	-1.81149100	3.17627500	2.76719200
H	-2.01012700	2.10420501	2.68407700
C	-2.80219600	3.88914800	1.83296300
H	-2.63062400	3.61252401	0.78919400

H	-3.82673400	3.59397201	2.08973100
H	-2.74637300	4.98063701	1.92385800
C	-2.06145200	3.60187100	4.22924500
H	-1.87379600	4.67517400	4.36174700
H	-3.10371001	3.40541400	4.51109300
H	-1.41595100	3.06062800	4.93039000
C	3.06616100	1.56616300	2.39342300
H	2.57331600	0.59366400	2.46999900
C	4.05014600	1.48011900	1.21427500
H	4.66424600	2.38336800	1.12178200
H	4.73271500	0.63395300	1.35937000
H	3.52520500	1.33003500	0.26484700
C	3.82487900	1.80499600	3.71486801
H	3.14361401	1.81542700	4.57426100
H	4.56580500	1.01276100	3.88067501
H	4.35704300	2.76423400	3.69626401
C	0.14330500	0.70308000	4.23853200
H	0.30040700	1.41276101	5.03513000
C	-0.15038500	-0.61814600	4.26330500
H	-0.30035000	-1.30015400	5.08512200
C	-0.40816600	-2.42522100	2.56903400
C	-1.71206500	-2.93524600	2.38869600
C	-1.83248200	-4.29403500	2.06644200
H	-2.81802200	-4.72048000	1.91017000
C	-0.71040400	-5.10986100	1.93801800
H	-0.83004700	-6.15862000	1.67870100
C	0.56258100	-4.58597900	2.14496800
H	1.42874900	-5.23408900	2.05134000
C	0.74393200	-3.23537000	2.47156500
C	-2.95867000	-2.07772400	2.59168900
H	-2.68372000	-1.03558900	2.40668500
C	-3.45980100	-2.19406600	4.04667200
H	-2.69337800	-1.89056900	4.76904400
H	-4.33735700	-1.55324500	4.19921100
H	-3.75189400	-3.22609400	4.28028500
C	-4.09404900	-2.41408800	1.61215400
H	-4.52191700	-3.40740300	1.79473600
H	-4.90374100	-1.68400200	1.72540300
H	-3.75184900	-2.36545400	0.57448800
C	2.14700500	-2.70982400	2.76504200
H	2.10280400	-1.61784700	2.79280400
C	3.17177000	-3.09276700	1.68419800
H	2.84837000	-2.76580200	0.69080000
H	4.13626399	-2.61687000	1.90043400

H	3.34302500	-4.17465500	1.64298200
C	2.61985300	-3.18719800	4.15342300
H	2.69381200	-4.28126600	4.19014600
H	3.61080600	-2.77521000	4.38176800
H	1.93088500	-2.87024800	4.94562000
Br	-2.55820000	0.64402200	-0.23016700

INT5

Pd	0.00000000	0.00000000	0.00000000
C	0.00000000	0.00000000	2.02300000
C	0.40003300	0.00000000	-2.35242700
H	0.11128900	1.02013100	-2.58776600
C	-0.59392200	-0.93602900	-2.16445100
H	-0.30679900	-1.97946599	-2.07207500
C	-2.04276000	-0.73426900	-2.35030500
C	-2.63571600	0.52592501	-2.56386600
C	-4.00473900	0.63248101	-2.79553900
C	-4.81268500	-0.51006500	-2.82188200
C	-4.23854500	-1.76528100	-2.61063200
C	-2.86822900	-1.87447100	-2.37537500
H	-2.02710300	1.42377900	-2.53674300
H	-4.44415800	1.61188401	-2.96543700
H	-5.87835201	-0.42083499	-3.01471300
H	-4.85409800	-2.66047300	-2.63869500
H	-2.42207500	-2.85482400	-2.22535700
C	1.83937100	-0.27673800	-2.52546400
C	2.38929000	-1.57426000	-2.52916900
C	2.70049399	0.81161200	-2.76257000
C	3.74940200	-1.76879700	-2.75973300
H	1.75083399	-2.43849701	-2.37183600
C	4.06203699	0.61520700	-2.99145100
H	2.29004699	1.81798600	-2.77460700
C	4.59316999	-0.67655300	-2.99066200
H	4.15125700	-2.77847900	-2.77397000
H	4.70458099	1.47039999	-3.18280600
H	5.65097200	-0.83438700	-3.18244800
N	-1.07160100	-0.19771000	2.84527400
C	-2.42554600	-0.50074100	2.42898800
N	1.06138700	0.15332000	2.86724300
C	-2.76398600	-1.84845500	2.18272000
C	-4.08376500	-2.12451700	1.80116200
H	-4.37596500	-3.14977300	1.59551900
C	-5.02634500	-1.10698500	1.68298400

H	-6.04251900	-1.34185500	1.37724600
C	-4.67166100	0.21089900	1.96090200
H	-5.42007300	0.99187900	1.87401900
C	-3.36652100	0.54751200	2.34408600
C	-1.77924300	-2.99800400	2.38198700
H	-0.77502800	-2.57637700	2.47491700
C	-1.75094900	-3.97555199	1.19470400
H	-1.55459700	-3.45215999	0.25342300
H	-0.95745400	-4.71874699	1.34004300
H	-2.69533600	-4.52172399	1.08765100
C	-2.08883800	-3.74540500	3.69500700
H	-3.08793200	-4.19751200	3.66597400
H	-1.36072199	-4.54970200	3.85897700
H	-2.04976900	-3.07241200	4.56029100
C	-3.02389900	1.98776500	2.71593300
H	-1.94219800	2.11313000	2.61621400
C	-3.68001500	3.02414699	1.78996700
H	-4.77121099	3.04381999	1.89765400
H	-3.30973699	4.02580500	2.03859600
H	-3.43331600	2.83107899	0.74242300
C	-3.40702100	2.26750900	4.18455800
H	-2.90176800	1.58442300	4.87718200
H	-3.13194700	3.29297900	4.46219600
H	-4.48862700	2.15706600	4.33508100
C	-0.68149600	-0.16721600	4.18227400
H	-1.38964200	-0.30684200	4.98329800
C	0.65396000	0.05302100	4.19608100
H	1.35339100	0.14644300	5.01127300
C	2.45344300	0.28888300	2.48981000
C	3.20032100	-0.88691800	2.26116800
C	4.55558800	-0.73989400	1.93669100
H	5.15818800	-1.62291800	1.74651600
C	5.14277900	0.51972300	1.85617600
H	6.19394600	0.61237300	1.59576300
C	4.38758200	1.66174000	2.11180700
H	4.86292700	2.63549300	2.05188800
C	3.02706300	1.57748500	2.43755200
C	2.60934500	-2.28516700	2.42259200
H	1.52111701	-2.19353700	2.46643300
C	3.07351500	-2.91231000	3.75275300
H	2.79059200	-2.29169400	4.61176100
H	2.62138000	-3.90287900	3.88817600
H	4.16355601	-3.03528500	3.77178000
C	2.93652500	-3.21453700	1.24139100

H	4.00541100	-3.45184700	1.18833200
H	2.39596901	-4.16254399	1.35173800
H	2.64317900	-2.76576300	0.28705000
C	2.23966300	2.84110799	2.77355500
H	1.17747100	2.62205599	2.63389200
C	2.57329000	4.02200000	1.84803900
H	2.43448100	3.75095400	0.79801100
H	1.89959000	4.85980900	2.06377400
H	3.59897000	4.38381100	1.98909400
C	2.46183400	3.24060100	4.24736200
H	3.51798899	3.47302499	4.43557700
H	1.87274700	4.13321400	4.49316600
H	2.16724200	2.44259899	4.93872700
H	0.25700200	-1.52308400	0.21238100
Br	-0.36159900	2.65058900	-0.19730900

In [BMIM]BF_4 with Ez = -0.002 a.u. :

Pd(0)

Pd	0.00000000	0.00000000	0.00000000
C	0.00000000	0.00000000	1.96949900
H	5.55173700	0.00000000	3.24576600
N	0.80486600	-0.71732300	2.82192400
C	1.83851700	-1.63629600	2.41293300
N	-0.80526800	0.71720600	2.82174800
C	1.50759100	-2.99684000	2.24300300
C	2.53425900	-3.87412100	1.86856501
H	2.31018300	-4.92840900	1.73210600
C	3.83665800	-3.41869299	1.67704800
H	4.61972700	-4.11669600	1.39168600
C	4.13977400	-2.07192500	1.86414000
H	5.16087400	-1.72818400	1.72415500
C	3.15226200	-1.15185800	2.24153900
C	0.09764300	-3.53655300	2.46326400
H	-0.55301400	-2.69670899	2.72193300
C	-0.47100300	-4.16906200	1.18008600
H	-0.47721400	-3.44272900	0.35811700
H	-1.50260100	-4.50232000	1.34763500
H	0.11281500	-5.04192900	0.86313400
C	0.05520000	-4.52988800	3.63929700
H	0.67974901	-5.41088100	3.44819800
H	-0.97130000	-4.87820500	3.80620500

H	0.40677700	-4.06555600	4.56770700
C	3.52745500	0.31235600	2.45057200
H	2.63756800	0.84680600	2.79431300
C	3.97229300	0.96870600	1.12982601
H	4.87191500	0.48842400	0.72495501
H	4.20305200	2.02890700	1.29218801
H	3.17948000	0.90472600	0.37411101
C	4.60670100	0.47368300	3.53735600
H	4.28789000	0.03022501	4.48753800
H	4.80921900	1.53686201	3.71456300
C	0.50421600	-0.45154200	4.15656400
H	1.03476600	-0.92475600	4.96859700
C	-0.50552200	0.45072000	4.15644700
H	-1.03650000	0.92361100	4.96838900
C	-1.83617000	1.63909900	2.41229800
C	-3.15127400	1.15867500	2.24059400
C	-4.13581500	2.08147200	1.86208600
H	-5.15791000	1.74074500	1.72200200
C	-3.82854200	3.42716900	1.67419200
H	-4.60932000	4.12733499	1.38785400
C	-2.52488200	3.87874299	1.86622000
H	-2.29755700	4.93230599	1.72950400
C	-1.50109700	2.99859499	2.24182400
C	-3.53124100	-0.30404800	2.45112100
H	-2.64253500	-0.84146100	2.79330300
C	-4.60894700	-0.46032900	3.54023499
H	-4.28623800	-0.01816100	4.48969699
H	-4.81595000	-1.52253500	3.71803299
H	-5.55229800	0.01782000	3.25040699
C	-3.98122900	-0.95974800	1.13177699
H	-4.88080500	-0.47748400	0.72924499
H	-4.21394300	-2.01937300	1.29508899
H	-3.19035300	-0.89777800	0.37386500
C	-0.08995100	3.53458799	2.46359400
H	0.55840599	2.69301699	2.72242000
C	0.48187299	4.16661199	1.18163000
H	0.48735999	3.44089799	0.35911100
H	1.51410699	4.49702599	1.35086800
H	-0.09917600	5.04125499	0.86455000
C	-0.04662700	4.52705099	3.64037300
H	-0.66882101	5.40964500	3.44890800
H	0.98048100	4.87269000	3.80907300
H	-0.40072000	4.06295800	4.56793999

INT1

Pd	0.00000000	0.00000000	0.00000000
C	0.00000000	0.00000000	2.09385200
C	0.64177200	0.00000000	4.28948500
N	1.04970700	0.13067900	2.96468200
C	2.42090700	0.37705600	2.58743700
N	-1.07016500	-0.21588200	2.92216300
C	2.86357900	1.71176100	2.47224900
C	4.20957800	1.92222400	2.14354900
H	4.58337700	2.93843500	2.05618700
C	5.07904200	0.85173400	1.94591700
H	6.12168500	1.03799900	1.70092900
C	4.61894000	-0.45622900	2.07943800
H	5.30919200	-1.28367000	1.94192600
C	3.28286800	-0.72438800	2.40703800
C	1.95173600	2.90741700	2.73232900
H	0.93069700	2.53635000	2.85638000
C	1.93434600	3.89131200	1.54909100
H	1.61991300	3.39276700	0.62484399
H	1.23024400	4.70808800	1.74956800
H	2.91955600	4.34113900	1.37708600
C	2.34482400	3.62482100	4.03849500
H	3.35868100	4.03891800	3.97803000
H	1.65462500	4.45261000	4.24135700
H	2.31254000	2.94264700	4.89555500
C	2.82549700	-2.16854200	2.59094100
H	1.74337900	-2.16603700	2.74850000
C	3.10025300	-3.02415100	1.34141999
H	4.17394801	-3.11530100	1.13736899
H	2.70447000	-4.03717401	1.48427199
H	2.62023500	-2.59504500	0.45407399
C	3.47248500	-2.79602900	3.84090300
H	3.24054101	-2.22013700	4.74400300
H	3.10244600	-3.81764701	3.98995600
H	4.56400701	-2.84452100	3.74549500
H	1.33296600	0.07557800	5.11486700
C	-0.69556800	-0.21882000	4.26257500
H	-1.40661801	-0.37599100	5.05905800
C	-2.43042500	-0.40828800	2.48091700
C	-3.27659600	0.71647200	2.38636200
C	-4.59837199	0.50121300	1.97364000
H	-5.27547099	1.34726700	1.89596300
C	-5.06071499	-0.77865900	1.67543300

H	-6.09195999	-0.92418900	1.36356700
C	-4.20858199	-1.87428300	1.79355300
H	-4.58316399	-2.87053600	1.57584200
C	-2.87782500	-1.71680200	2.20468800
C	-2.81525700	2.12884200	2.73356200
H	-1.75105400	2.09033700	2.98200500
C	-3.55723400	2.66700200	3.97200600
H	-3.41521800	2.01300600	4.83987100
H	-3.18249900	3.66299200	4.23693800
H	-4.63528900	2.75264600	3.78949700
C	-2.96725300	3.08923400	1.54033699
H	-4.01769200	3.21275500	1.24981599
H	-2.57812100	4.08034100	1.80421299
H	-2.41218900	2.72791300	0.66673599
C	-1.98653100	-2.94559300	2.35909000
H	-0.99571899	-2.61151500	2.67924100
C	-1.80729300	-3.68900800	1.02265500
H	-1.37658200	-3.02998900	0.25879900
H	-1.13253200	-4.54401000	1.15332600
H	-2.76187300	-4.07288500	0.64229400
C	-2.52474499	-3.89115700	3.44978000
H	-3.50913799	-4.29566499	3.18511900
H	-1.84165899	-4.73779500	3.58878100
H	-2.62166799	-3.37624500	4.41232799
C	-0.11002600	-0.08692700	-2.10138000
C	-1.50502800	-0.11264600	-2.37160000
C	0.57986200	1.14886700	-1.97520500
C	-2.18590100	1.08924800	-2.53513300
H	-2.01897400	-1.06211800	-2.48009200
C	-0.14984800	2.34932300	-2.15131700
C	-1.50759800	2.31996300	-2.44623300
H	-3.25423900	1.06977700	-2.73484900
H	0.37879400	3.29652800	-2.08073300
Br	0.95784100	-1.73843200	-2.65105000
H	1.66297400	1.17676000	-1.92192200
H	-2.05110100	3.24857000	-2.59733900

TS1

Pd	0.00000000	0.00000000	0.00000000
C	0.00000000	0.00000000	2.11352800
N	1.06612000	0.00000000	2.97270400
C	2.45016200	-0.04371200	2.56708700
N	-1.08131700	0.04280500	2.95244900

C	3.12580400	1.17112400	2.32567700
C	4.47339500	1.09978500	1.94793900
H	5.02408700	2.01799300	1.76358900
C	5.12210800	-0.12683900	1.82183100
H	6.17008900	-0.15913800	1.53458300
C	4.43547200	-1.31082800	2.08092100
H	4.95552500	-2.26084500	1.99707900
C	3.08705000	-1.29780000	2.46352900
C	2.46097700	2.53248001	2.50621100
H	1.39815400	2.36952701	2.70545100
C	2.55685400	3.39943701	1.23801900
H	2.11230100	2.89017701	0.37464500
H	2.01930600	4.34373101	1.38826000
H	3.59604500	3.64730701	0.99014900
C	3.05010200	3.27256900	3.72272200
H	4.11805399	3.48127400	3.58529200
H	2.53764600	4.23063601	3.87234000
H	2.93978800	2.68563600	4.64150500
C	2.37932100	-2.61206500	2.78194800
H	1.33277700	-2.39020000	3.00813200
C	2.39171000	-3.57711999	1.58266300
H	3.41179299	-3.87602199	1.31239401
H	1.83335199	-4.48908000	1.82724001
H	1.92585700	-3.12141000	0.70097301
C	2.98809500	-3.27746600	4.03132100
H	2.94347100	-2.61243600	4.90134800
H	2.44012599	-4.19390400	4.28148100
H	4.03837799	-3.54886299	3.87059000
C	0.65755800	0.04091000	4.30417800
H	1.36102300	0.04423200	5.12276100
C	-0.69714300	0.06725200	4.29137200
H	-1.41512100	0.09703300	5.09674700
C	-2.45729900	0.06818700	2.51943400
C	-3.08421600	1.31709400	2.32627700
C	-4.42727801	1.31389100	1.92620600
H	-4.94084300	2.25979100	1.77872000
C	-5.11705201	0.11947099	1.72881400
H	-6.16093701	0.13943699	1.42596100
C	-4.47603601	-1.10030800	1.93399700
H	-5.02695601	-2.02597400	1.79270200
C	-3.13456501	-1.15524200	2.33628600
C	-2.36884600	2.64318600	2.56807900
H	-1.31872800	2.42892900	2.78530200
C	-2.95374100	3.36928600	3.79473300

H	-2.88646400	2.75110800	4.69709200
H	-2.40655100	4.30058800	3.98471100
H	-4.00915100	3.62576800	3.64262700
C	-2.40057100	3.55259100	1.32605900
H	-3.42272000	3.85804900	1.07143700
H	-1.82027100	4.46420100	1.51401100
H	-1.96894800	3.05003800	0.45211301
C	-2.47596501	-2.50762801	2.59194200
H	-1.42338000	-2.33440001	2.83177900
C	-2.51474601	-3.41206101	1.34658700
H	-2.02711801	-2.92990601	0.49064700
H	-1.98866400	-4.35300201	1.54895800
H	-3.54238800	-3.66289401	1.05684800
C	-3.11497600	-3.21167201	3.80427700
H	-4.17431000	-3.43335201	3.62683400
H	-2.60398300	-4.16035201	4.00831100
H	-3.04851900	-2.59419701	4.70722399
C	-0.07947800	0.27953500	-2.00605800
C	-1.43527500	0.58041200	-2.26516800
C	0.94155900	1.21426500	-2.28864700
C	-1.76283900	1.85672800	-2.73502300
H	-2.20360900	-0.17074300	-2.11406000
C	0.58153000	2.48150200	-2.75862300
C	-0.76164100	2.80122700	-2.98891100
H	-2.80661800	2.10643600	-2.90987700
H	1.35893300	3.21704400	-2.95112800
Br	0.48952800	-1.85251700	-2.25550800
H	1.98433400	0.94603200	-2.15379600
H	-1.02774400	3.78474400	-3.36669300

INT2

Pd	0.00000000	0.00000000	0.00000000
C	0.00000000	0.00000000	2.16415100
N	1.01931900	0.00000000	3.07311500
C	2.42578000	-0.07865100	2.75230600
N	-1.12064100	0.12402000	2.93537400
C	3.08950700	1.09136400	2.32923300
C	4.45534200	0.99098800	2.03066200
H	4.99376900	1.87385600	1.69766299
C	5.13552800	-0.21550500	2.17425800
H	6.19764000	-0.26999700	1.94928400
C	4.46193200	-1.34894500	2.62439800
H	5.00860100	-2.27825200	2.75229300

C	3.09342501	-1.31008100	2.92297800
C	2.38979200	2.44142800	2.20404500
H	1.37103800	2.33715900	2.58804099
C	2.28511900	2.87679600	0.73121099
H	1.75330300	2.12289000	0.13447899
H	1.74238100	3.82612001	0.64631699
H	3.27787800	3.01481100	0.28482299
C	3.08159800	3.52440400	3.05198599
H	4.10046899	3.72910300	2.70289900
H	2.51735999	4.46330900	2.99794099
H	3.14015100	3.22827900	4.10534499
C	2.39568601	-2.55620300	3.46196300
H	1.31564501	-2.38795600	3.41449300
C	2.69516401	-3.81359400	2.62706000
H	3.75375801	-4.09508800	2.67344900
H	2.11627201	-4.66079000	3.01424500
H	2.42082101	-3.66706200	1.57650699
C	2.76525601	-2.78608400	4.94157499
H	2.51352601	-1.91843500	5.56181799
H	2.22380801	-3.65267000	5.33949299
H	3.83905800	-2.97895600	5.05557099
C	0.54273800	0.13250800	4.37506099
H	1.20373300	0.16320700	5.22745600
C	-0.80802701	0.20995000	4.28849499
H	-1.56521401	0.32195200	5.04929999
C	-2.46379900	0.19860300	2.41047200
C	-2.94886600	1.45037100	1.97657600
C	-4.24648299	1.49491300	1.44847000
H	-4.64832999	2.44212499	1.10021500
C	-5.03591199	0.34904600	1.38485900
H	-6.04452199	0.40687300	0.98354100
C	-4.54517199	-0.86694300	1.85573400
H	-5.18039300	-1.74715400	1.82452600
C	-3.24935699	-0.97313400	2.37881900
C	-2.12919599	2.73344200	2.08440500
H	-1.21744200	2.51051100	2.64590800
C	-2.88157800	3.82761300	2.86475800
H	-3.18408300	3.47429600	3.85687400
H	-2.23536800	4.70276900	3.00170800
H	-3.78246499	4.16034000	2.33612000
C	-1.70222500	3.24640500	0.69703900
H	-2.57304200	3.48187600	0.07244500
H	-1.09972900	4.15777500	0.79278400
H	-1.10232100	2.49755700	0.16112600

C	-2.75867599	-2.30853100	2.93213900
H	-1.68429799	-2.22575400	3.12142200
C	-2.95397999	-3.46817400	1.94023600
H	-2.44318299	-3.27090600	0.99088299
H	-2.53496499	-4.38983900	2.36178000
H	-4.01362799	-3.65690200	1.73102900
C	-3.44428299	-2.61836400	4.27788300
H	-4.52968399	-2.71909800	4.15589900
H	-3.06381399	-3.55925700	4.69301400
H	-3.26238399	-1.82759800	5.01451600
C	-0.10064800	0.33142800	-1.96757400
C	-1.36503700	0.49934300	-2.55206800
C	1.04432700	0.61670700	-2.72596200
C	-1.48397000	1.00107500	-3.85434800
H	-2.26468600	0.25085100	-1.99169200
C	0.92398500	1.11967000	-4.02740600
C	-0.33959200	1.31364100	-4.59420500
H	-2.47157100	1.14075900	-4.28980600
H	1.82004901	1.35317900	-4.59945700
Br	0.38784100	-2.44918600	-0.62919800
H	2.03450701	0.45534500	-2.30536200
H	-0.43144700	1.69798600	-5.60761699

INT3

Pd	0.00000000	0.00000000	0.00000000
C	0.00000000	0.00000000	2.21429500
N	1.12984400	0.00000000	2.99473800
C	2.50104800	-0.18491000	2.55710200
N	-1.01674800	0.11494700	3.12668900
C	3.32607700	0.94784500	2.37007800
C	4.65185700	0.72291800	1.97321200
H	5.31067300	1.57027600	1.81471500
C	5.14779100	-0.56607300	1.79804500
H	6.18087000	-0.71381800	1.49350400
C	4.33273400	-1.66588900	2.04897000
H	4.74397200	-2.66609200	1.95627800
C	2.99949799	-1.50398300	2.45084700
C	2.85607700	2.36911600	2.67000900
H	1.76990400	2.39634900	2.54837600
C	3.44717500	3.42228401	1.71790800
H	3.29205400	3.15349300	0.66921999
H	2.95388000	4.38606801	1.88998100
H	4.51964901	3.57463201	1.88946000

C	3.19451800	2.75230100	4.12741100
H	4.27908201	2.74312000	4.29291900
H	2.83054700	3.76378500	4.34532800
H	2.74095700	2.07228100	4.85487600
C	2.19027299	-2.73042800	2.86663300
H	1.12794499	-2.46760900	2.84401200
C	2.39556399	-3.94224000	1.94018100
H	3.39566899	-4.37617900	2.05381100
H	1.67377799	-4.72820800	2.19157700
H	2.26174299	-3.68280600	0.88389601
C	2.53011699	-3.11924700	4.32160800
H	2.32260499	-2.30057900	5.01850300
H	1.93649799	-3.98700800	4.63380500
H	3.59020699	-3.38302500	4.41779500
C	0.81675400	0.11752700	4.34702500
H	1.57564100	0.12045200	5.11287600
C	-0.52975599	0.19539900	4.43026600
H	-1.18491999	0.28953300	5.28170300
C	-2.44096000	0.03878800	2.86899400
C	-3.14967700	1.21581900	2.54329800
C	-4.53150099	1.09989300	2.33517800
H	-5.10892100	1.98337100	2.08307800
C	-5.18444700	-0.12316800	2.47165800
H	-6.25832299	-0.18492000	2.31486400
C	-4.46911600	-1.26037800	2.83671300
H	-4.99547199	-2.20012800	2.97173100
C	-3.08466800	-1.20799700	3.05248000
C	-2.47968500	2.58650900	2.50656800
H	-1.42636400	2.44439599	2.25161200
C	-2.55073100	3.24785200	3.89965000
H	-2.07768600	2.62955099	4.66995800
H	-2.03715000	4.21698600	3.88740500
H	-3.59143300	3.42088500	4.20085600
C	-3.07292900	3.52876300	1.44700100
H	-4.09512100	3.83871400	1.69584900
H	-2.46450000	4.43794600	1.38465200
H	-3.08363100	3.06826900	0.45330399
C	-2.35150600	-2.45768800	3.54355900
H	-1.27745900	-2.30692200	3.39196900
C	-2.74560400	-3.73975500	2.78770600
H	-2.62425600	-3.63716400	1.70392800
H	-2.11143500	-4.57023100	3.11971400
H	-3.78398999	-4.02811800	2.98621100
C	-2.58899900	-2.65754900	5.05549700

H	-3.65085700	-2.83879300	5.26118400
H	-2.02330500	-3.52379700	5.41866300
H	-2.28017800	-1.78411600	5.63839600
C	-1.47475000	-1.83063000	-0.18993300
H	-2.00585700	-1.61623200	0.73143300
C	-0.15634500	-2.23831300	-0.09298400
H	0.38792500	-2.60756700	-0.95648200
H	0.25197600	-2.49157300	0.87633300
C	0.26304100	0.03256400	-2.02769600
C	1.46440400	-0.48381800	-2.53909700
C	1.73140400	-0.47502000	-3.91537500
C	0.79631600	0.05292100	-4.80874301
C	-0.40343600	0.57311700	-4.31202801
C	-0.66282100	0.56536800	-2.93703901
H	2.21124300	-0.89964400	-1.86440000
H	2.67217300	-0.88077000	-4.28393300
H	0.99983800	0.06121500	-5.87763101
H	-1.13985100	0.99201800	-4.99614901
H	-1.59782500	0.98646300	-2.57598501
C	-2.34704900	-1.89279800	-1.37576400
C	-2.00114800	-2.60209900	-2.54030100
C	-3.60923000	-1.27326200	-1.31598200
C	-2.88613400	-2.67625700	-3.61411300
H	-1.04412500	-3.11124800	-2.59899400
C	-4.49179899	-1.34296500	-2.39323399
H	-3.89488800	-0.73548500	-0.41569599
C	-4.13220000	-2.04399800	-3.54727999
H	-2.60614600	-3.23554500	-4.50323700
H	-5.46146200	-0.85684300	-2.32740699
H	-4.82260400	-2.10755600	-4.38453300
Br	0.35796500	2.56907100	-0.14228100

TS2

Pd	0.00000000	0.00000000	0.00000000
C	0.00000000	0.00000000	2.11747400
N	1.17030600	0.00000000	2.83497400
C	2.49968500	-0.28962900	2.33263200
N	-0.96850700	0.18810300	3.06774400
C	3.42207301	0.76606300	2.15501800
C	4.70175501	0.43446000	1.68541600
H	5.43232601	1.22290300	1.53561200
C	5.06139101	-0.88480500	1.42841000
H	6.06070601	-1.11558900	1.06780400

C	4.15492500	-1.91340200	1.67278900
H	4.46344100	-2.94232000	1.51828300
C	2.86442101	-1.64499400	2.14781500
C	3.11786001	2.21251700	2.53645800
H	2.03217201	2.33261400	2.57468200
C	3.65188901	3.23298800	1.51700800
H	3.28498301	3.01929300	0.50930300
H	3.30585101	4.23685300	1.79043200
H	4.74825900	3.26179000	1.50115700
C	3.69613801	2.52756400	3.93310800
H	4.79000601	2.44231000	3.93153200
H	3.44010901	3.55272300	4.22731400
H	3.31273201	1.85302600	4.70526500
C	1.95734301	-2.80303600	2.55762900
H	0.92233101	-2.45120300	2.53594899
C	2.05240101	-4.02495700	1.62731399
H	3.01384401	-4.54166200	1.72821599
H	1.27091501	-4.74792000	1.88870499
H	1.92458501	-3.75442400	0.57277500
C	2.26452301	-3.22719900	4.01011700
H	2.14652801	-2.39344400	4.71003600
H	1.58622801	-4.02989100	4.32418999
H	3.29239201	-3.59959600	4.09980100
C	0.92994100	0.20515600	4.19196000
H	1.72627800	0.23181400	4.91841201
C	-0.40730600	0.33110200	4.33612000
H	-1.01407100	0.49593100	5.21200000
C	-2.40502100	0.04967100	2.91268400
C	-3.18165900	1.17109000	2.55053600
C	-4.56817600	0.98938700	2.44687100
H	-5.19659600	1.82950100	2.16949900
C	-5.15994200	-0.23994200	2.72583700
H	-6.23830700	-0.35353100	2.64860800
C	-4.37671200	-1.31343700	3.13944300
H	-4.85364500	-2.25461300	3.39469200
C	-2.98497599	-1.19461900	3.25435400
C	-2.57620600	2.56111599	2.38735000
H	-1.52901501	2.44935199	2.09689500
C	-2.61939500	3.31329599	3.73430400
H	-2.07643001	2.77419600	4.51852000
H	-2.16096401	4.30486899	3.63220400
H	-3.65223201	3.45268199	4.07768700
C	-3.25328200	3.39959699	1.29159700
H	-4.27917400	3.68189999	1.55811700

H	-2.68930000	4.32639799	1.13939900
H	-3.28130200	2.86678899	0.33450500
C	-2.18234499	-2.36100700	3.83178000
H	-1.12041699	-2.18142100	3.63818100
C	-2.53650399	-3.71982100	3.20391400
H	-2.43890399	-3.70376300	2.11397800
H	-1.86383299	-4.49218600	3.59602800
H	-3.55981499	-4.02902500	3.44631700
C	-2.37346499	-2.43147900	5.36239401
H	-3.42054199	-2.63733900	5.61494501
H	-1.76137099	-3.23719700	5.78519500
H	-2.08807399	-1.49783600	5.85761700
C	-0.56389200	-2.06454600	-0.24418500
H	-0.09822700	-2.56381800	0.59956300
C	0.25459000	-1.86455900	-1.39380500
H	-0.16623600	-2.09486400	-2.36566300
H	1.31894401	-2.06791700	-1.30780000
C	0.33580000	0.08012100	-2.13715700
C	1.63887400	0.35460800	-2.57884201
C	1.85721200	0.98716100	-3.80562001
C	0.77531800	1.34638200	-4.61511501
C	-0.52664400	1.05943000	-4.19074101
C	-0.74408600	0.41681800	-2.96964601
H	2.48968400	0.08282900	-1.95908400
H	2.87461300	1.20220500	-4.12600201
H	0.94469200	1.83769600	-5.57050401
H	-1.37621100	1.32860300	-4.81539201
H	-1.75928400	0.17170900	-2.66861101
C	-2.01877300	-2.37367100	-0.45492600
C	-3.01681400	-1.39155801	-0.52574300
C	-2.39336400	-3.71829500	-0.63328800
C	-4.34803200	-1.73879301	-0.77476500
H	-2.74704500	-0.35158801	-0.36794400
C	-3.72224200	-4.06696900	-0.88176400
H	-1.63410800	-4.49513700	-0.57741100
C	-4.70564300	-3.07627101	-0.95472500
H	-5.10573400	-0.96063501	-0.81365600
H	-3.98903900	-5.11278700	-1.01253900
H	-5.74207400	-3.34624601	-1.14023300
Br	0.33020500	2.63954100	-0.01423200

INT4

Pd	0.00000000	0.00000000	0.00000000

C	0.00000000	0.00000000	2.15304700
C	0.25086500	0.00000000	-2.09109400
H	-0.56085400	0.53836800	-2.58010700
C	0.42863100	-1.41231500	-2.64012000
H	-0.51941500	-1.94253000	-2.50307800
H	1.18829600	-1.96187800	-2.07486300
C	0.79896100	-1.41058500	-4.11640300
C	-0.19687100	-1.32500500	-5.10189600
C	0.13405300	-1.29415700	-6.45847800
C	1.47295700	-1.34931800	-6.85611800
C	2.47458600	-1.43561900	-5.88546000
C	2.13868300	-1.46599200	-4.52969200
H	-1.24140600	-1.28810600	-4.79985400
H	-0.65513800	-1.23238500	-7.20443200
H	1.73308600	-1.32828800	-7.91178700
H	3.51976700	-1.48369500	-6.18205600
H	2.92537300	-1.53700400	-3.78216099
C	1.37642600	0.83908500	-1.71867700
C	2.50027100	0.29611700	-1.03138100
C	1.32057600	2.25630500	-1.87747500
C	3.54941500	1.12863600	-0.60838700
H	2.60047500	-0.77844100	-0.92344900
C	2.36689600	3.06091400	-1.46506700
H	0.45059100	2.69058500	-2.36361900
C	3.49716100	2.49706800	-0.84135700
H	4.40950099	0.69011400	-0.11097901
H	2.31881600	4.13482399	-1.62481200
H	4.32090500	3.13537500	-0.53508501
N	0.10857500	-1.11212100	2.94843100
C	0.43243900	-2.44980599	2.50085900
N	-0.25795200	1.00737900	3.04571300
C	1.78876800	-2.77384899	2.26162000
C	2.07779900	-4.07817800	1.83873300
H	3.10700900	-4.36156200	1.64426400
C	1.07024000	-5.02860500	1.68869100
H	1.31956000	-6.03682400	1.36733000
C	-0.24950899	-4.69823500	1.98041800
H	-1.02035099	-5.45766599	1.89454200
C	-0.60086999	-3.40716800	2.40065600
C	2.92618600	-1.79387499	2.54726600
H	2.54585700	-0.77776800	2.40652900
C	4.13681900	-1.96336999	1.61430500
H	3.84586600	-1.96956699	0.55784600
H	4.83953200	-1.13698399	1.77152500

H	4.68522600	-2.89110199	1.81660500
C	3.38258500	-1.92044799	4.01702699
H	3.76778900	-2.92713100	4.22027899
H	4.18425600	-1.20288300	4.23030299
H	2.56373500	-1.72539799	4.71679099
C	-2.04264199	-3.11678400	2.81156700
H	-2.17949299	-2.03181200	2.82811800
C	-3.08015899	-3.69420500	1.83409000
H	-3.08136199	-4.79087300	1.83805800
H	-4.08437899	-3.36936700	2.13242200
H	-2.90452399	-3.34347400	0.81292099
C	-2.31013099	-3.65565900	4.23319300
H	-1.61352299	-3.24430500	4.97089199
H	-3.32760899	-3.39952800	4.55256200
H	-2.21465099	-4.74826800	4.25991500
C	-0.08147400	-0.80136200	4.29333599
H	-0.02669400	-1.54728500	5.07017899
C	-0.31727300	0.52872800	4.35380199
H	-0.50979700	1.17802699	5.19329899
C	-0.31998800	2.42504600	2.75633700
C	0.83079100	3.20347000	3.01437400
C	0.75002700	4.58215100	2.77235000
H	1.61453200	5.20930000	2.96761800
C	-0.42524200	5.16281900	2.30569500
H	-0.46775100	6.23438101	2.12734600
C	-1.55610700	4.37734700	2.09257400
H	-2.47377500	4.85047900	1.75882700
C	-1.53819900	2.99534700	2.32537900
C	2.10919901	2.62048200	3.61477200
H	2.06380001	1.53066600	3.52882400
C	2.20849801	2.96846200	5.11480300
H	1.34117301	2.60764000	5.67728300
H	3.10614201	2.51465300	5.55182500
H	2.27459401	4.05321500	5.26271900
C	3.38186001	3.07948000	2.88409800
H	3.56364601	4.15341700	3.00842900
H	4.25319101	2.55386700	3.29300200
H	3.32832301	2.86504100	1.81235000
C	-2.82056100	2.17592900	2.20417700
H	-2.54626700	1.15123800	1.93998900
C	-3.76648400	2.68090500	1.10335000
H	-3.25348200	2.77222500	0.13982300
H	-4.58967600	1.96960600	0.97426100
H	-4.21050600	3.65234600	1.35260800

C	-3.56038800	2.13888100	3.55864900
H	-3.85522400	3.14752600	3.87446400
H	-4.47039600	1.53138700	3.47843600
H	-2.94062800	1.70745800	4.35178200
Br	-2.48891600	-0.64400400	-0.48677000

TS3

Pd	0.00000000	0.00000000	0.00000000
C	0.00000000	0.00000000	2.07592000
C	0.45220500	0.00000000	-2.17254600
H	-0.43376600	-0.43705300	-2.62445900
C	1.42642200	-0.89798400	-1.65399499
H	1.42337800	-0.76203100	-0.05579299
H	2.45624300	-0.54536300	-1.64030299
C	1.30797000	-2.37364600	-1.84281699
C	0.08852400	-3.06004900	-1.71830799
C	0.02195300	-4.43278900	-1.95229599
C	1.17182800	-5.14591101	-2.30767700
C	2.39090800	-4.47508101	-2.42253400
C	2.45865300	-3.09994000	-2.18746100
H	-0.80212600	-2.51833400	-1.41132299
H	-0.92694600	-4.95002300	-1.83827299
H	1.11899300	-6.21801201	-2.47776200
H	3.29324400	-5.02128301	-2.68517499
H	3.41150300	-2.58264300	-2.27200399
C	0.76035700	1.37553700	-2.60515400
C	1.89092700	2.09066300	-2.16056500
C	-0.10129700	2.00759000	-3.52308799
C	2.14630600	3.38188900	-2.62038399
H	2.56704900	1.64568500	-1.43668299
C	0.15863600	3.29639400	-3.98605399
H	-0.98458800	1.47639600	-3.86719399
C	1.28489300	3.99225800	-3.53725999
H	3.01869500	3.91603800	-2.25287200
H	-0.52384100	3.76110100	-4.69375500
H	1.48560800	5.00052400	-3.89006499
N	0.51436100	0.98571300	2.86925100
C	1.24195600	2.15180200	2.41861900
N	-0.49904100	-0.91554000	2.95815000
C	0.54895000	3.37085200	2.25682300
C	1.29845300	4.49179700	1.87476300
H	0.80090700	5.44806800	1.74829600
C	2.67383500	4.40620300	1.67028100

H	3.23488500	5.29210801	1.38339700
C	3.33473300	3.19413000	1.85286400
H	4.41098000	3.14548200	1.71521400
C	2.63760400	2.04088600	2.23825900
C	-0.94324500	3.50320800	2.54751800
H	-1.38656100	2.50543300	2.48023900
C	-1.68087300	4.38903300	1.53009600
H	-1.54504400	4.02094100	0.50832100
H	-2.75562600	4.37694300	1.74700700
H	-1.35288300	5.43437000	1.57685600
C	-1.16855200	4.03051500	3.97997700
H	-0.75985300	5.04199800	4.09754300
H	-2.24112600	4.07274200	4.20518400
H	-0.69408500	3.38996200	4.73106900
C	3.40538500	0.74982100	2.51066800
H	2.68176700	-0.06229100	2.62147800
C	4.34435100	0.36068000	1.35544300
H	5.15591800	1.08560600	1.22116000
H	4.80599700	-0.61248400	1.56219000
H	3.80010400	0.28104600	0.40681000
C	4.18752300	0.85499000	3.83486001
H	3.52199500	1.07221900	4.67768601
H	4.70523300	-0.08786100	4.04910500
H	4.94127900	1.65049100	3.78914001
C	0.33850900	0.68872400	4.21970800
H	0.68024800	1.34929700	5.00087700
C	-0.29609300	-0.50477700	4.27536700
H	-0.61813400	-1.09925700	5.11569601
C	-1.04443800	-2.21552600	2.62990800
C	-2.44038300	-2.35988200	2.47517500
C	-2.92531500	-3.64443900	2.19346400
H	-3.99266100	-3.79678300	2.07014100
C	-2.06549700	-4.73580800	2.08740000
H	-2.46796100	-5.72336001	1.87616400
C	-0.69652600	-4.56989800	2.27784400
H	-0.04078500	-5.43367600	2.22393400
C	-0.15404400	-3.30894101	2.56191600
C	-3.40833600	-1.19823000	2.68508500
H	-2.87104900	-0.27041400	2.46777900
C	-3.88298300	-1.15088200	4.15347900
H	-3.04743400	-1.05753900	4.85490500
H	-4.54871200	-0.29328200	4.30978000
H	-4.43838300	-2.06002500	4.41576000
C	-4.62528900	-1.24101800	1.74670100

H	-5.29416700	-2.07869000	1.97832800
H	-5.20690500	-0.31942800	1.86478200
H	-4.32297300	-1.30943500	0.69716800
C	1.33628200	-3.17712001	2.86666200
H	1.59406000	-2.11444901	2.86478500
C	2.22890701	-3.85888301	1.81634599
H	2.01444601	-3.49152401	0.80705700
H	3.28348001	-3.65239901	2.03643599
H	2.10436901	-4.94796401	1.81450799
C	1.64505200	-3.72163901	4.27619500
H	1.41787600	-4.79233600	4.34581699
H	2.70729000	-3.58659501	4.51345400
H	1.06061001	-3.20446901	5.04530699
Br	-2.38482300	1.04625500	-0.43949600

INT5

Pd	0.00000000	0.00000000	0.00000000
C	0.00000000	0.00000000	2.02882400
C	0.40505800	0.00000000	-2.34997900
H	0.03236600	0.98617100	-2.60796901
C	-0.50445600	-1.01446900	-2.14365700
H	-0.13257900	-2.02844000	-2.02688100
C	-1.96525400	-0.93780400	-2.34027100
C	-2.67237700	0.27210200	-2.48635500
C	-4.04674100	0.26539100	-2.71273500
C	-4.74812601	-0.94244401	-2.80163200
C	-4.06015900	-2.14881401	-2.65657000
C	-2.68433801	-2.14486301	-2.42626800
H	-2.15090800	1.21867099	-2.38401900
H	-4.57661100	1.20955399	-2.81020700
H	-5.82140601	-0.94049601	-2.97274700
H	-4.59369900	-3.09394201	-2.71547300
H	-2.15405601	-3.08718501	-2.31117700
C	1.86645700	-0.15140500	-2.49177500
C	2.53773500	-1.38783600	-2.41059800
C	2.62865500	1.00072500	-2.76459901
C	3.91724599	-1.46161800	-2.59063700
H	1.98263100	-2.29806800	-2.20516500
C	4.00951000	0.92539500	-2.94348301
H	2.12628900	1.96292900	-2.82060000
C	4.66136800	-0.30694900	-2.85654701
H	4.41557199	-2.42487500	-2.51869100
H	4.57633000	1.83082601	-3.14455201

H	5.73808400	-0.36885100	-2.99031900
N	-1.07039800	-0.17977300	2.85328900
C	-2.42967900	-0.46491100	2.44578300
N	1.06592300	0.14550800	2.86420300
C	-2.78135000	-1.80727800	2.18890200
C	-4.10649100	-2.06748800	1.81476100
H	-4.41221400	-3.08972501	1.61321200
C	-5.04249500	-1.04073700	1.72296400
H	-6.06737000	-1.26526000	1.43839400
C	-4.67635200	0.26958299	2.02145400
H	-5.42452000	1.05460400	1.97582600
C	-3.36473000	0.59086399	2.39663200
C	-1.80961601	-2.96755300	2.39064000
H	-0.80039501	-2.55789500	2.48345400
C	-1.79252001	-3.94854900	1.20617200
H	-1.59027501	-3.43068200	0.26202600
H	-1.00911501	-4.70150200	1.35596500
H	-2.74381701	-4.48376200	1.10236600
C	-2.12810901	-3.70704200	3.70599500
H	-3.13121601	-4.14975001	3.67928000
H	-1.40683101	-4.51588400	3.87497200
H	-2.08328001	-3.03132800	4.56751100
C	-3.01726700	2.01611100	2.81909100
H	-1.93200001	2.13442900	2.75348600
C	-3.64187700	3.08717299	1.91050800
H	-4.73584400	3.10351799	1.98421500
H	-3.28279700	4.07809599	2.21323300
H	-3.35876600	2.93492999	0.86452100
C	-3.43512300	2.25216400	4.28603900
H	-2.96345600	1.53560400	4.96700000
H	-3.14667300	3.26016800	4.60802400
H	-4.52185000	2.15906100	4.40399600
C	-0.67469800	-0.14467700	4.18900000
H	-1.38048500	-0.26894900	4.99509900
C	0.66271900	0.06145500	4.19599100
H	1.36517199	0.15425700	5.00904299
C	2.45694900	0.27487300	2.48479000
C	3.21352100	-0.90413600	2.31458700
C	4.57352999	-0.76034200	2.00813800
H	5.18730999	-1.64665399	1.87695800
C	5.15451599	0.49953300	1.89235700
H	6.21336699	0.58928000	1.66321000
C	4.38759099	1.64600000	2.08786800
H	4.86045699	2.61997800	2.01301200

C	3.02247799	1.56483500	2.39431800
C	2.62687200	-2.29862200	2.51867400
H	1.53892200	-2.20732600	2.57636600
C	3.11101900	-2.90012200	3.85310500
H	2.83861000	-2.26520300	4.70357500
H	2.66145500	-3.88766299	4.01345800
H	4.20103700	-3.02149400	3.86148900
C	2.93774700	-3.24916300	1.34927000
H	4.00796700	-3.47695800	1.27970500
H	2.40937000	-4.19987599	1.49068600
H	2.61943500	-2.82135600	0.39201200
C	2.22081199	2.83190200	2.67953900
H	1.16234799	2.60034800	2.53070700
C	2.56189299	3.98628900	1.72333399
H	2.45205399	3.68232000	0.67779899
H	1.87551699	4.82265400	1.89983999
H	3.57998599	4.36374100	1.87768499
C	2.41326599	3.27856800	4.14399599
H	3.46256999	3.52808500	4.34499799
H	1.80936799	4.17024400	4.35170499
H	2.11363799	2.49976799	4.85337300
H	0.33302000	-1.49558300	0.27266600
Br	-0.46942800	2.63600900	-0.34256200

10. Supplementary References

1 Weisenfeld, R. B. & Miller, M. D. Protection of a 2,6-dialkylaniline: Synthesis of 4-dimethylmethoxysilyl-2,6-diethylaniline. *Synth. Commun.* **16**, 809−817 (1986).

2 Ishiyama, T., Murata, M. & Miyaura, N. Palladium(0)-catalyzed cross-coupling reaction of alkoxydiboron with haloarenes: A direct procedure for arylboronic esters. *J. Org. Chem.* **60**, 7508−7510 (1995).

3 Grimm, S. H. *et al.* Comprehensive structure-activity-relationship of azaindoles as highly potent flt3 inhibitors. *Bioorgan. Med. Chem.* **27**, 692−699 (2019).

4 Ni, S. *et al.* Novel inhibitors of staphyloxanthin virulence factor in comparison with linezolid and vancomycin versus methicillin-resistant, linezolid-resistant, and vancomycin-intermediate staphylococcus aureus infections in vivo. *J. Med. Chem.* **60**, 8145−8159 (2017).

5 Zinser, C. M. *et al.* A simple synthetic entryway into palladium cross-coupling catalysis. *Chem. Commun.* **53**, 7990−7993 (2017).

6 Diéguez, H. R. *et al.* Weakening C−O bonds: Ti(iii), a new reagent for alcohol deoxygenation and carbonyl coupling olefination. *J. Am. Chem. Soc.* **132**, 254−259 (2010).

7 Hruszkewycz, D. P., Balcells, D., Guard, L. M., Hazari, N. & Tilset, M. Insight into the efficiency of cinnamyl-supported precatalysts for the Suzuki-Miyaura reaction: Observation of Pd(i) dimers with bridging allyl ligands during catalysis. *J. Am. Chem. Soc.* **136**, 7300−7316 (2014).

8 Watson, I. D. G., Ritter, S. & Toste, F. D. Asymmetric synthesis of medium-sized rings by intramolecular Au(i)-catalyzed cyclopropanation. *J. Am. Chem. Soc.* **131**, 2056−2057 (2009).

9 Zhou, F., Zhou, F., Su, R., Yang, Y. & You, J. Build-up of double carbohelicenes using nitroarenes: Dual role of the nitro functionality as an activating and leaving group. *Chem. Sci.* **11**, 7424−7428 (2020).

10 Yang, C. *et al.* Unveiling the full reaction path of the Suzuki-Miyaura cross-coupling in a single-molecule junction. *Nat. Nanotechnol.* (2021) doi: https://doi.org/10.1038/s41565-021-00959-4.

11 Becke, A. D. Density-functional exchange-energy approximation with correct asymptotic behavior. *Phys. Rev. A* **38**, 3098−3100 (1988).

12 Lee, C., Yang, W. & Parr, R. G. Development of the colle-salvetti correlation-energy formula into a functional of the electron density. *Phys. Rev. B* **37**, 785−789 (1988).

13 Petersson, G. A. et al. A complete basis set model chemistry. I. The total energies of closed‐shell atoms and hydrides of the first‐row elements. *J. Chem. Phys.* **89**, 2193−2218 (1988).

14 Hay, P. J. & Wadt, W. R. Ab initio effective core potentials for molecular calculations. Potentials for the transition metal atoms Sc to Hg. *J. Chem. Phys.* **82**, 270−283 (1985).

15 Zhao, Y. & Truhlar, D. G. A new local density functional for main-group thermochemistry, transition metal bonding, thermochemical kinetics, and noncovalent interactions. *J. Chem. Phys.* **125**, 194101−194118 (2006).

16 Andrae, D., Häußermann, U., Dolg, M., Stoll, H. & Preuß, H. Energy-adjustedab initio pseudopotentials for the second and third row transition elements. *Theor. Chem. Acc.* **77**, 123−141 (1990).

17 Marenich, A. V., Cramer, C. J. & Truhlar, D. G. Universal solvation model based on solute electron density and on a continuum model of the solvent defined by the bulk dielectric constant and atomic surface tensions. *J. Phys. Chem. B* **113**, 6378−6396 (2009).

18 Gaussian 09, Revision D.01, Frisch, M. J. *et al.* Gaussian, Inc., Wallingford CT, 2013.

19 Bernales, V. S., Marenich, A. V., Contreras, R., Cramer, C. J. & Truhlar, D. G. Quantum mechanical continuum solvation models for ionic liquids. *J. Phys. Chem. B* **116**, 9122−9129 (2012).

20 Wakai, C., Oleinikova, A., Ott, M. & Weingärtner, H. How polar are ionic liquids? Determination of the static dielectric constant of an imidazolium-based ionic liquid by microwave dielectric spectroscopy. *J. Phys. Chem. B* **109**, 17028−17030 (2005).

21 Tariq, M., Forte, P. A. S., Gomes, M. F. C., Lopes, J. N. C. & Rebelo, L. P. N. Densities and refractive indices of imidazolium- and phosphonium-based ionic liquids: Effect of temperature, alkyl chain length, and anion. *J. Chem. Thermodyn.* **41**, 790−798 (2009).

22 Huddleston, J. G. *et al.* Characterization and comparison of hydrophilic and hydrophobic room temperature ionic liquids incorporating the imidazolium cation. *Green Chem.* **3**, 156−164 (2001).

23 Brandbyge, M., Mozos, J.-L., Ordejón, P., Taylor, J. & Stokbro, K. Density-functional method for nonequilibrium electron transport. *Phys. Rev. B* **65**, 165401 (2002).

24 Taylor, J., Guo, H. & Wang, J. Ab initio modeling of quantum transport properties of molecular electronic devices. *Phys. Rev. B* **63**, 245407 (2001).

25 Perdew, J. P., Burke, K. & Ernzerhof, M. Generalized gradient approximation made simple. *Phys. Rev. Lett.* **77**, 3865−3868 (1996).
