# Supplementary material for: Precise electrical gating of the single-molecule Mizoroki-Heck reaction
Source: Nat Commun. 2022 Aug 5;13:4552. doi: 10.1038/s41467-022-32351-8 (PMC9355990; doi:10.1038/s41467-022-32351-8)
Supplement: Supplementary file 2 — Description of Additional Supplementary Information [file 41467_2022_32351_MOESM2_ESM.pdf]

File Name: Supplementary Movie 1

Description: Fluorescent and current signals at the single-molecule catalyst site during the Mizoroki-Heck cross-coupling. The real-time monitoring of the blinking catalyst site shows that the fluorescent and current signals are highly correlated. The electrical current signal (blue line) and the optical fluorescent intensity (flesh pink line) are displayed via 3× slowdown.

File Name: Supplementary Software 1

Description: The Python programs for data analysis. The code can be utilised to analyse the numbers and values of the current levels as well as the current level transformations.
